# Experiences of friendships of young people with first-episode psychosis: A qualitative study

**DOI:** 10.1371/journal.pone.0255469

**Published:** 2021-07-30

**Authors:** Catherine Huckle, Frederike Lemmel, Sonia Johnson

**Affiliations:** 1 School of Psychology, University of Surrey, Guildford, Surrey, United Kingdom; 2 Division of Psychiatry, University College London, London, United Kingdom; Case Western Reserve University, UNITED STATES

## Abstract

**Background:**

First episode psychosis and reduced social networks have been found to go hand in hand, but specific mechanisms are unclear. The manifestation of symptoms and the effect of stigma are two possibilities discussed in the literature but the experiences and views of young people with psychosis have been neglected.

**Aims:**

To explore experiences of friendships of young people with first-episode psychosis, focusing especially on any perceived changes in their friendships or approach to peer relationships as a result of the illness.

**Methods:**

Fourteen participants were interviewed using a semi-structured interview guide, which explored participants’ views and experiences of their friendships during the acute phase of illness and in the path to recovery, the impact of friendships on illness experience and of illness on patterns of social contact, and the potential role of services in supporting people with their friendships. Interviews were transcribed verbatim and analysed thematically.

**Results:**

Identified themes included the loss of social contacts because both young people developing psychosis withdrew *and* because friends withdrew as illness developed. Regarding recovery, a unique role was identified for friends and participants were often making conscious efforts to rebuild social networks. Mental health services were viewed as having a limited direct role in this.

**Conclusions:**

Supporting the development of opportunities and skills needed for social relationships following an episode of psychosis may be a useful focus.

## Introduction

### Psychosis and friendships: Stigma and symptoms

First episode psychosis has been found to be associated with social difficulties such as interpersonal discomfort, loneliness, isolation and poor perceived social support [1–3]. However, the causality and specific mechanisms remain unclear.

Previous literature suggests that individuals experiencing first-episode psychosis have reduced social networks (friends and confidants rather than family) and less access to social support compared to others [4]. It appears that this constricted social support and reduced satisfaction with social contacts precedes onset of a diagnosis of psychosis [2, 4] (however, it is worth noting that fewer friends and greater loneliness than healthy controls are also reported for people with at risk mental states across diagnostic categories [5]). These findings are of clinical importance as frequency of contact with friends in early psychosis has been found to be a predictor of clinical recovery, though direction of causality is uncertain [6].

If social networks are already limited at an early stage, early intervention with a social focus may be important. Two explanations have been put forward for the deterioration of social networks associated with early psychosis. Firstly, both stigma and self-stigma have potentially serious impact on social interactions [7, p. 225], and it seems this may be particularly significant for people living with psychosis (for example, individuals with psychosis report more rejection experiences than individuals with other mental disorders [8]). One potential reason is that there is a common perception in the general population that individuals with symptoms of psychosis may behave violently [9]. Less explicit fears are also known; individuals suffering from psychotic illness can struggle to fit into normal patterns of social interaction, and this can feel uncomfortable and awkward for those around them resulting in reduced social contacts and peer relationships breaking down [10]. However, research has tended to be carried out in relation to hypothetical situations [11]: it is not clear whether *established* peer friendships break down as a result of stigmatising attitudes of the other.

Self-Stigma could also account for a reduction in social contacts, and refers to the internalisation of negative perceptions of oneself: living in a society that endorses stigmatising ideas may lead individuals to believe they are less valued, impacting on self-esteem and confidence [12]. Accordingly, theory in social psychology suggests that reciprocity is key to successful friendships [13], but clients with severe mental illness can feel that they have little social currency; they may have few physical or emotional resources [14]. Individuals report missing friendship reciprocity in the asymmetrical friendships that often characterise the vulnerable state of mental illness [15]. Some may believe their illness to be so repellent that they are reluctant to subject their friends and family to their positive symptoms, and so withdraw from these relationships [14].

It is also possible that individuals with psychosis withdraw because their symptoms make it difficult to cope in social situations. Rofe [16] documented that stress can motivate individuals to seek out the company of others, but only when those others might serve to reduce the stress experienced. It then follows that individuals experiencing psychosis might withdraw from others, as there is the potential for the presence of others to exacerbate stress levels, particularly if symptoms include delusions and hallucinations. So-called positive symptoms may have thematic content directly at odds with friendship (ideas of paranoia, for example) or may involve a process that indirectly interferes with positive social interaction (such as distraction by voices). [17]. Further explicating this relationship, Davidson [15] reports that individuals suffering from psychosis may feel vulnerable to external stimulation and therefore choose to withdraw from peer relationships to protect themselves.

### The role of peer relationships in recovery

Reduction in social relationships has a significant impact on recovery from psychosis, and the size of social network has been found to account for 12.2% of the variance in Quality of Life scores, an established measure of recovery [8]. This effect is specific to friendship rather than family interaction and is significantly predictive of clinical recovery from first-episode psychosis [6].

One of the mechanisms by which social interaction may have a positive effect on recovery is by enabling individuals to ‘step out’ of the illness [18] and to develop a sense of belonging and hope, which leads to a lesser need for paranoid beliefs [15]. Participants have identified the journey from “social exclusion to social inclusion” (p58) as key to their concept of recovery from psychosis [19]. Opportunities to build reciprocal and supportive relationships during hospitalisation and service-led activities may be the first stage in this process of ‘belonging’. However, as with all human relationships, social contact comes with a risk of feelings of rejection, particularly caused by stigma, de-socialisation, exclusion and loss of roles. This dichotomy has been described as the “double-edged” (p56) nature of social factors in recovery [20].

As might be expected as a consequence of reduced social contacts, high levels of loneliness in individuals experiencing psychosis have been identified [21]. There is growing interest in developing interventions to combat loneliness and social isolation among people recovering from mental health problems but substantial evidence is limited [22, 23]. Understanding disruptions to close relationships at the early stages of psychosis has potential to inform interventions to prevent long-term loneliness. This, taken with the clear impact of social relationships on recovery, provide a strong rationale for learning more about the initial mechanisms that lead to reduced social networks. In this paper we aim to explore in depth experiences of friendship and their maintenance or disruption in the early stages of psychosis.

## Methods

The study comes under the heading "Human Subject Research" and was approved by the North West London REC 1 Panel (REC reference approval number: 10/H0722/35). Written, informed consent was obtained from all participants to have their data included in the study and excerpts of interviews included in the final manuscript.

### Aims

The study aimed to answer the following research questions:

1. How do people with first-episode psychosis experience friendships?
2. How do they perceive any changes in their social contacts or their approach to peer relationships?
3. What are their perceptions about what was helpful or unhelpful about friendships in their recovery?
4. Do they perceive a role for services in the establishment or maintenance of friendships?

### Design

We invited potential participants to take part regardless of engagement with services or level of social functioning.

The methodology was qualitative and used thematic analysis, as it has been specifically developed to systematically examine the way in which participants make sense of their experiences [24]. The accounts were collected by interview, recorded and transcribed. The research aimed to identify themes that have emerged across participants.

### Setting

This study took place at an NHS early intervention service for psychosis in London. The service catered for young people between the ages of 18 and 34 who were local residents and had entered the service after experiencing a first identified episode of psychotic illness. The service provided care co-ordination, therapeutic intervention, psychiatric evaluation and medication, support to access education, training or employment, relapse prevention and crisis planning. The service also offered social activities and groups such as badminton, walking groups and cinema trips.

### Participants

#### Recruitment and sample size

For the purposes of this study first-episode psychosis was operationally defined as ‘first treatment contact’ for psychosis [25], in line with the service in which recruitment took place. The limitations of this are acknowledged in terms of the potential for symptoms to be undetected for a period of time and a possible delay in accessing services. For this reason, Duration of Untreated Psychosis (DUP) was collected as reported in clinical notes. This refers to the time between first (detected) onset of psychotic symptoms and the point of accessing treatment. Staff were asked to identify clients on their caseloads who may meet the inclusion criteria:

- Referred to the team at least six months prior to the interview, as it is more likely that these participants would have had time to develop their own narrative of their experience [26].
- Experienced more than one episode of illness, or that they have suffered one episode that has lasted more than one month and has been accompanied by significant and prolonged decline in function.
- Due to limited resources, participants who were not English speaking were not included.

Care co-ordinators approached potential participants and provided information about the study. If participants consented, their contact details were passed to the researcher who contacted them by telephone to allow an opportunity to ask questions, and following this an interview appointment was arranged.

**Fig 1:**
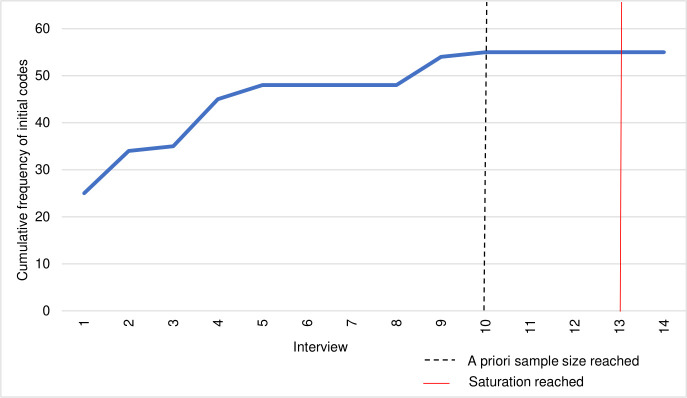
Cumulative frequency of initial codes.

A total of 14 participants were recruited. Sample size was identified using an adaptation of the process described by Francis et al. (2010) [27]: The initial analysis sample size was defined a priori as ten interviews with the stopping criterion defined as three interviews. Purposive sampling was used to include people with a range of characteristics and to reduce the possibility of a ‘false’ saturation that can occur with homogeneous samples. As new codes emerged at interview nine, recruitment was re-started and four further interviews were carried out (see S1 Appendix for more details of evaluation of saturation, and S2 Appendix for summary table of initial codes used to assess whether the stopping criterion had been achieved). At re-evaluation after interview 14 the stopping criterion was reached and code and meaning saturation were assumed (see Fig 1. for pattern of cumulative frequency).

#### Participant characteristics

See Table 1 for characteristics of participants.

**Table 1:** Characteristics of participants.

Participant Number:	Age	Gender	Ethnicity	DUP	Time between referral to service & research interview
				(Months unless otherwise stated)	(Months)
1	26	F	White European	2	8
2	21	F	Black-British	20	15
3	22	M	White British	10	24
4	28	M	White British	12	27
5	31	F	Asian	36 hours	16
6	26	M	White British	2	22
7	19	M	Black African	Not recorded	6
8	27	F	White European	Not recorded	16
9	27	F	Black British	1	10
10	34	F	Asian	6	29
11	23	M	White European	2	34
12	20	M	White European	3 weeks	23
13	24	F	White European	Not recorded	24
14	24	M	White European	1	14

DUP = Duration of Untreated Psychosis.

### Semi-structured interviews

Semi-structured interviews were carried out to explore personal experiences while specifically focusing on perceptions of change in friendships and the possible role of friendships in recovery. Questions were developed to explore mechanisms of change, the “double-edged” nature of social factors in recovery [20] and the mixed literature regarding the helpfulness of services in maintaining social functioning [28, 29]. Interviews were discussed at two service-user forum groups to ensure the validity of the questions and to gather feedback about wording and general approach to the subject matter. It was piloted with the first two participants with particular attention given to the apparent ease of the participant, the impact and accessibility of the wording of the questions, the depth of the answers given and the length of the interviews. Participants in these interviews spoke openly and in detail, and the length of the interviews was within the parameters set (first interview: 38 minutes, second interview: 30 minutes). Initial coding of these transcripts indicated that the questions yielded relevant experiences and ideas (34 codes were initially identified, see S2 Appendix). The interview schedule was considered to be successful in its aims and it was not modified.

The interview had four broad stages characterised by current friendships, pre-morbid friendships, the role of friendships in recovery and gaps in social connections / how services could help (see S3 Appendix for full interview schedule).

The time spent in each section of the interview varied as the researcher was guided by the participant’s responses. Thirteen out of 14 of the interviews lasted between 30 minutes and 45 minutes. One interview lasted just 15 minutes as the participant appeared reluctant to discuss their experiences. The (limited) data was still included in the analysis. All interviews were recorded on a digital voice recorder and transcribed by the researcher verbatim.

### Analysis

Thematic analysis was conducted [30] from an essentialist stance and the intention in this study is to report participants’ experiences, with the assumption that language used by participants directly reflects the meaning intended.

Analysis began with reading and re-reading of the transcripts. Initial ideas were annotated. Initial codes were generated systematically and data was collated under these codes electronically, using Microsoft Word documents. An ‘inductive’ approach was used (whereby we approached the data without preconceived themes and development of codes was driven by the data). Initial codes were labeled using key words or phrases from the data extracts.

The researchers and two colleagues were randomly allocated two transcripts to code to check that salient data had not been excluded or misinterpreted by the lead researcher and to reduce the impact of ordering effects (it is suggested that this is necessary to prevent a false sense of saturation that can emerge when interviews are coded sequentially and not re-visited [31]. Initial codes were reviewed (see S2 Appendix for summary table of initial codes) and reduced from a total of 57 to 27 codes. The team began to group codes into potential themes and a draft thematic map was completed.

The lead researcher returned to the data, reviewed transcripts and identified instances of replication of meaning in the 27 codes and refined codes further, leading to the final number of 17 codes. The final 17 codes were systematically reviewed by the research team by returning to the transcripts of origin to ensure that data under each code had not been extrapolated from its original meaning. Themes were defined by the lead researcher and discussed with the research team to ensure that they represented the coded extracts accurately and that they worked in relation to the data set as a whole without overlap or replication of concepts. These were organised into a final thematic map with three broad domains and seven overall themes. The thematic map was discussed by the team to ensure that it meaningfully reflected the data and that interpretations of the data were explicit and specific. See S1 Appendix for more detail about the process of analysis.

## Results

The analysis highlighted three main domains of experience; ‘friendship losses’, ‘friends during recovery’ and ‘moving on from here’ and were characterised by a sense of change in friendships during the course of their illness. An overview of domains, themes and sub-themes is given in Table 2.

**Table 2:** Overview of themes and sub-themes.

Domains	Themes	Sub-themes
**Friendship losses**	***1*. *Self stigma*: *Participant directed loss of social contact***	1.1 Anxiety “I didn’t want them to see any difference in me”. (Participant 1)
		1.2 Talking about illness “I didn’t want to explain”. (Participant 4)
	***2*. *Symptoms ended friendships as I knew them***	2.1 Symptoms directly affected friendships: “I accused him of doing things which I believed he did, but they weren’t true”. (Participant 10)
		2.2 Rejection by others: “they’re like ‘there’s something wrong with you”. (Participant 9)
		2.3 Friends moved on: “I would have been more similar to them if I hadn’t been in hospital”. (Participant 6)
	***3*. *Friendships incompatible with recovery***	
**Friends during recovery**	***4*. *Getting better*: *What can friends do to help*?**	4.1 Everyday support and distraction: “I think what would have helped was someone who could make you go out more.just get things out of your mind for a while”. (Participant 11)
		4.2 Play a unique role: “it was good to have someone who wasn’t related to me and was just my friend”. (Participant 5)
	***5*. *Assessment of current social situation***	5.1 Absence of romantic relationships: “I have got a gap; I would like a man”. (Participant 9)
		5.2 Absence of shared history: “the place of old friends is always different; you can never replace them”. (Participant 13)
		5.3 Feeling back to normal socially: “I’ve become confident again, talking to people”. (Participant 12)
		5.4 Friendships stronger and closer: “he showed me that he was there for me”. (Participant 2)
**Moving on from here**	***6*. *Making new friends***	6.1 Conscious effort: “I’m practising meeting new people, practising putting my barriers down”. (Participant 9)
		6.2 Ego strengthening and reciprocity: “he stands on my shoulders, I stand on his shoulders”. (Participant 11)
		6.3 Getting life back on track: “First on my list is trying to get a job, second is getting my flat sorted, third is start being a bit more sociable”. (Participant 4)
	***7*. *The role of services***	7.1 Facilitate sharing of experiences: “it sort of relaxes you as well to know you’re not the only one”. (Participant 10)
		7.2 A need to step away from services: “I don’t know if people would really want them interfering in their friendships”. (Participant 6)

Each theme and its’ constituent sub-themes are discussed and illustrated with quotations from interview transcripts. The participant number of the source of each quotation is indicated.

### Friendship losses

#### Theme 1: Self-stigma

Ten out of the fourteen participants indicated that social contacts had been reduced because of their *anticipation* of a negative reaction from others. Participant experiences fall into two sub-themes.

*1*.*1 Anxiety*: *“I didn’t want them to see any difference in me” (Participant 1)*. Seven participants described anxiety that their friends would notice changes in their personality or patterns of interacting. They described feeling different and had lower confidence in their ability to interact than they had before the onset of psychosis.

*“If they wanted to go out I was worried that I wouldn’t be the same person; I wouldn’t be like the joker anymore*.*” (Participant 7)*

There seemed to be a sense that it would not be possible to live up to friends’ expectations of them and a difference in their general abilities would be noticed:

*“I just felt that she’s going to come and my house won’t be tidy enough*. *It’s too many little things; food should be ready*, *and I won’t be able to manage*.*” (Participant 10)*

Participants also indicated a concern that others would treat them differently, and that they would be placed in a different role within the relationship compared to before the psychosis:

*“I feel like it’s my friends used to call me ‘Big [participant name]’*. *I used to like it*, *you know*? *I felt*‥ *not powerful*, *powerful is not the word*. *I felt strong*. *I felt that I played a strong role*. *It would make me feel really different now*. *(Participant 4)*

*1*.*2 Talking about the illness*: *“I didn’t want to explain”*. *(Participant 4)*. Nine participants described a reluctance to see friends because they did not want to give an explanation for their “absence”.

For most participants this was because of a fear of other people’s reactions and the impact on their perception of the participant or their wider family.

*“I’m not speaking exactly what happened because I know that for the people it’s sometimes a bit scary*, *so I don’t want to scare them*.*” (Participant 1)*

For others it was because the process of talking about their experiences would contain an element of re-living a distressing time, which was too upsetting for them to confront.

*“I don’t like talking about it and when I do I get really upset and stuff like that so I don’t like talking to people*, *even friends*, *about what happened*, *what I went through”*. *(Participant 11)*

A small group of participants (four out of fourteen) described a different experience and were keen to talk to others openly about their experiences, to prevent relationships progressing on a ‘false’ basis.

*“I wanted the truth to come out so that in the future if we get married*, *if he finds out he won’t leave me or he won’t get angry at me*, *so I told him from the start” (Participant 13)*

#### Theme two: ‘Symptoms ended friendships as I knew them’

Eleven participants described experiences of reduced social contact as a result of the explicit manifestation of both negative and positive symptoms.

*2*.*1 Symptoms directly affected friendships*: *“I accused him of doing things which I believed he did*, *but they weren’t true”*. *(Participant 10)*. Nine participants described friends becoming integrated into their experience of their symptoms. For some this led to reduced contact as a result of fear:

*“When my mental problems started happening*, *I stopped being sociable with my friends*. *That’s when I used to lock myself away and used to have bad dreams and bad nightmares*, *and my friends would be in some of my dreams and nightmares*.*” (Participant 4)*

For others, their behavioural reaction to their friends during their illness meant that relationships were damaged:

*“I think I lost them before I fell ill*, *or I was already ill but not diagnosed*. *I just* … *I was not nice or polite to them anymore so we lost contact*.*” (Participant 10)*

*2*.*2 Rejection by others*: *“*‥*they’re like ‘there’s something wrong with you’*.*” (Participant 9)*. Five participants interviewed described experiences of friends ‘disowning’ them as a result of “strange” or unusual behaviour during their illness:

*“*.*I went to see him and then over there I was acting really weird as well and I got arrested by the police over there*. *It was like a really small village and it*, *sort of*, *brought shame on them*. *…*‥*they found out here in London*, *then they just stopped talking to me*.*” (Participant 14)*

*2*.*3 Friends moved on*: *“I would have been more similar to them if I hadn’t been in hospital”*. *(Participant 6)*. Six participants described an experience of stagnation during their illness, as symptoms forced a period of absence from ‘normal life’ while peers continued to progress with expected milestones. Participants seemed to indicate a sense of being left behind with less things in common with their friends than before their illness:

*“I feel especially now they*’*ve all got children that their priorities have changed” (Participant 5)*

Talking to old friends seemed to make participants’ lack of progress more painful as it highlighted this period of stagnation:

*“I didn’t want to talk to them because I knew it would be all about going back to uni …it would remind me of all the good times that I’m missing out on*.*” (Participant 7)*

#### Theme three: Friendships incompatible with recovery: ‘I don’t need to miss them’

Nine participants described making a conscious decision to distance themselves from friends in the interests of their recovery from psychosis:

*“I need to stay away from friends that go out all the time*, *every weekend they get drunk and pass out” (Participant 14)*.

For the majority of these participants this was related to drug and alcohol use in their social circles, which was perceived as a contributing factor to the onset of psychosis. Participants prioritised recovery over social contacts as it didn’t seem possible to be involved with the same friends without drug and alcohol use beginning again.

*“When I came out of the hospital the first thing I done was get some drink*, *which I shouldn’t have done*, *and went and sat up in my friend’s house*‥ *And after that I haven’t really seen them that much because I don’t want to go up there and start drinking again*.*” (Participant 4)*

### Friends during recovery

#### Theme four: Getting better: What can friends do to help?

Eight participants described a constructive role for friends during recovery from psychosis. Participant experiences straddle two sub-themes.

*4*.*1 Everyday support and distraction*: *“I think what would have helped was somebody who could make you go out more*‥*just get things out of your mind for a while”*. *(Participant 11)*. Participants emphasised the importance of having friends around to distract from ruminating about the illness rather than focusing on or discussing illness-related experiences:

*“I think it would have helped me mentally*, *with my mind*, *just not thinking about problems*, *thinking about the bad all the time–negative*, *negative*, *negative*. *Instead of thinking about the positive things in life*, *the good things*, *the simple things in life*.*” (Participant 4)*

There was a shared idea that friends could provide support for participants to get back involved with everyday life, and to resume activities which might have been difficult to initiate alone:

*“You need someone to help you go out more*, *like go to the cinema*‥ *like play football*, *like someone who’s a really close friend”*. *(Participant 11)*

*4*.*2 Play a unique role*: *“it was good to have someone who wasn’t*‥ *related to me and was just my friend”*. *(Participant 5)*. Participants experienced friendships differently to family relationships in the recovery process, which seemed to be related to a sense of less pressure or expectation:

*“*‥*my parents and my sister were always trying to tell me what to do …‘you should do this*, *you should do that*, *you should think about talking to this person*, *you should think about doing that’ and my friend was like ‘do what you want*!*’*. *(Participant 5)*

#### Theme five: Assessment of current social situation

Half of the participants felt that there were significant deficiencies in their current social situations, while a further five participants described experiences of social contacts returning to their pre-illness state. Eight participants identified positive impacts of illness and recovery on relationships that had endured.

*5*.*1 Romantic relationships*: *“I have got a gap; I would like a man”*. *(Participant 9)*. Three participants specifically highlighted the wish for a romantic relationship, and the absence of this was directly attributed to experiences of illness:

*“Thoughts of like having a girlfriend have pretty much gone out of the window over the last couple of years since being in hospital*, *so that’s something I definitely think about*.*” (Participant 6)*

There was also a sense that a romantic relationship might be important to moving on and progressing into adulthood, which might have been interrupted by psychotic illness:

*“You know like when you’re young*, *like a teenager in school and you have a best friend you know*, *that best friend*, *do you know what I mean*? *But as you get older it gets replaced with a man*, *kind of thing*. *I haven’t moved on to that bit yet*.*” (Participant 9)*

*5*.*2 Absence of shared history*: *“the place of old friends is always different*, *you can never replace them”*. *(Participant 13)*. Five participants highlighted the importance of a shared history and its contribution to a strong and reliable friendship. There was a sense that new friends (without a shared history) could not be fully trusted until some sort of hardship had been experienced (and weathered) together and so a sense of vulnerability prevailed:

*“You don’t really meet someone and you’re like ‘oh you’re my friend’; it takes a lot of tests to pass before you can say that person is a really good friend*.*” (Participant 2)*

For some participants this included experiencing the participant in an acute phase of illness, and there seemed to be an anxiety about this occurring and the potential impact on new friendships:

*“They are good friends and I like to go out with them and speak and everything but I’m not sure if they would be there if I was ill again*. *…Hopefully I will not have to realise that part of the friendship*.*” (Participant 1)*.

*5*.*3 Feeling back to normal socially*: *“I’ve become confident again*, *talking to people” (Participant 12)*. Five participants described a return to ‘normality’, and after evaluating their current social situation appeared to be satisfied that there were no gaps or absences:

*“I feel much better than before* …*My head’s in the right direction*. *I’ve got a balance now with my social life”*. *(Participant 11)*

*“The friends that I have now are pretty much the same as the ones I had before I had my episode*” *(Participant 5)*

*5*.*4 Friendships stronger and closer*: *“he showed me that he was there for me”*. *(Participant 2)*. Eight participants described experiences of stronger and closer relationships because of the support they received from their friends during their illness and recovery. This was strongly related to a sense that friends who remained in contact proved that they could be relied upon even in the most difficult of circumstances.

*“We’ve become much closer since the breakdown*‥ *She’s just there all the time either to talk or if I felt I didn’t want to stay on my own or something*. *No*, *she was like there for me but not in a way that was judgmental*.*” (Participant 9)*

### Moving on from here

#### Theme six: Making new friends

Participants interviewed had strong views on making new friends and the role this might play in ‘moving on’ from their illness, but there was also a sense of personal responsibility for this process.

*6*.*1 Conscious effort*: *“I’m practising meeting new people*, *practising putting my barriers down”*. *(Participant 9)*. Nine participants described conscious efforts directed at making new social contacts and this seemed to extend beyond the initial phase of meeting new people:

*“*.*To give you a metaphor… It’s like a tray with loads of glasses full of water and another tray on top of that and piling up and then you’ve got to keep like water in all of these trays but you’ve got to walk and run and stuff like that*. *To keep the tray balanced it’s like constant*, *something I’ve got to be constantly concentrating on*.*” (Participant 9)*

Participants described setbacks but emphasised the importance of carrying on despite any distress:

*it is an effort*, *it is effort and every time I get quite hurt*, *you know?” (Participant 8)*

*“[it takes] perseverance really*, *getting up in the morning and putting yourself in that situation*. *The days that are bad*, *going ‘ok*, *it’s a bad day*, *just get on with it; scream and shout and cry and it’ll be better tomorrow*” (Participant 5)

*6*.*2 Ego strengthening and reciprocity*: *“he stands on my shoulders*, *I stand on his shoulders” (Participant 11)*. Ten participants highlighted the importance of friendships being reciprocal, allowing them to contribute something to the relationship and to feel valued:

*“*‥*if he needs any help at all*, *I would be willing to help him as much as he wants*, *as much as I can*.*” (Participant 12)*

For some participants this had been lost in existing friendships and so was particularly important in new social contacts to allow ego strengthening and re-building of confidence:

*“Maybe it would be nice to have somebody that I can sort of look out for as much as they’d look out for me because I think that*, *my old friends*, ‥*They’re probably looking out for me*, *in a way looking after me and sometimes I don’t particularly like that because it makes you feel*‥ *I don’t know*‥ *fragile*.*” (Participant 6)*

*6*.*3 Getting life back on track*: *“First on my list is trying to get a job*, *second is getting my flat sorted*, *third is start being a bit more sociable*.*”*. *(Participant 4)*. Five participants highlighted a desire to ‘get life back on track’, in terms of management of symptoms, housing, employment and/or education. For some participants this was perceived as integral to having the confidence to meet new friends and progress socially:

*“I think I’ll become more independent and more*, *I think I’ll feel more happier you know*, *in myself*, *with myself*. *I don’t need to say I live at home with my parents–I have my own apartment*, *would you like to come round and watch a DVD*‥*” (Participant 4)*

*“I think I need to get my confidence back I think*, *but without having to be in contact with my friends*. *It’s like*, *maybe getting a job; my brother’s telling me like door-to-door salesman job*. *He done it before and he was telling me it increased his confidence*.*” (Participant 7)*

For other participants, it was perceived that ‘getting back on track’ would be a logistical opportunity to meet new people rather than part of the emotional or symptomatic journey of recovery:

*“I’m thinking about applying to university to start next September so I think it all goes well then that’ll be a good opportunity to meet new people*‥*” (Participant 6)*

*“Going to do a course*, *getting my own place*, *you know*, *stuff like that*. *I just wanted to do something to make new friends*.*” (Participant 11)*.

#### Theme seven: The role of services

All of the participants expressed views about the role of services in facilitating social recovery in terms of maintaining existing social contacts and developing new friendships. However, there was discrepancy both between and within participants in terms of the actual and potential helpfulness of organised intervention, represented in the following two sub-themes.

*7*.*1 Facilitate sharing of experiences with peers*: *“It sort of relaxes you as well to know you’re not the only one”*. *(Participant 10)*. Twelve participants described the helpfulness of discussing experiences with others who had a shared experience of psychosis. For some participants this was achieved through contact with other patients during periods of hospitalisation or from attendance at groups provided by the service, and it seemed to be the sense of acceptance and a reduced sense of self-stigma and isolation that was helpful:

*“I think partly you’ve got things in common with them and they’re all kind of*, *trying to get past their problems and I think it’s just nice to know there are other people who are experiencing the same things as you*.*” (Participant 6)*

Some participants felt that their wish to discuss experiences with peers had not been met by the services:

*“Most of them just want to keep themselves to themselves*. *I know how it is*‥ *I don’t like to go and hassle them*.*” (Participant 11)*

As a result of this participants described accessing other resources independently to meet the same need:

*“I spent a lot of time surfing the internet and mental health forums looking about what other people were posting about their experiences*‥ *There was something about hearing about other people’s experiences*, *even if it was just on a computer through forums” (Participant 5)*

*7*.*2 A need to step away from services*: *“I don’t know if people would really want them interfering in their friendships” (Participant 6)*. Five participants explicitly said that they would prefer to avoid service-led activities and rejected the opportunity to engage with other service-users. Interestingly, four of these participants had also identified a shared experience of psychosis helpful, but their rejection of services seemed to be because of wish to avoid re-visiting the distress of their experience:

*“I’ve*, *sort of*, *moved on–I don’t want to be around people that remind me of what happened*.*” (Participant 14)*

*“Coming here is a reminder of what happened before*, *right*? *Psychosis and that*. *Coming here for the social thing reminds me of all the bad stuff that’s happened*.*” (Participant 7)*

Nine participants expressed views that changes had to ultimately be made by the individual and that both formal and informal intervention from others may be helpful at the start but to a limited extent:

*“It’s me now*. *I’ve had all this helping me*, *going badminton*, *museums*, *cinemas*, *playing pool*, *going bowling*, *doing everything*. *I’ve done all that and really enjoyed it*, *but I’ve realised that now it’s time to get on with my life*.*” (Participant 4)*

## Discussion

### Summary of findings

The reported findings demonstrated experiences of loss of social contacts as a result of first episode psychosis, resulting from either participants or friends withdrawing, and highlighted the intense effort and vulnerability involved in building new relationships for this client group. However, the picture was not entirely negative; the data illustrated the strengthening of existing relationships and participants identified a unique role for friends in the process of recovery. In fact, a small number of participants reported that the experience had not adversely affected social relationships over the long term. The data powerfully illustrated the determination of participants interviewed to rebuild relationships and the importance of developing reciprocal relationships. While a role for services was identified in the facilitation of sharing experiences, participants also believed that the role of services was limited.

### Results in the context of the literature

Research suggests that the ‘other’ can feel apprehensive about interacting with individuals following an episode of psychosis [10], but the rate of actual rejections reported by participants is lower than expected from the stigma literature (for example, 9 out of 10 participants receiving secondary mental health care reported experiencing stigma, [32]). There are several possible explanations for this. Participants who had withdrawn from social interactions may have successfully avoided this rejection (albeit with other negative consequences). Participants may have not reported instances of rejection because it felt painful or humiliating to do so. Alternatively, participants taking part in this research were asked to provide tangible examples of rejection from others, while the majority of the existing literature seems to ask stigmatised individuals about their experiences in a general way (and so may absorb the effect of self stigma) OR asks the general public (potential persecutors) about their hypothetical reaction to individuals with psychosis. Research by Phelan and Link [9] found that people who reported more contact perceived people with mental illness as less dangerous than those who had little or no contact. It seems that the effect of stigma may be moderated by an established relationship with the other.

In terms of self-stigma, participants described a sense of anxiety that an altered self-presentation would be noticed by their friends or that they would have nothing to contribute to their friendships. Consequently their preference was to be isolated rather than to risk this happening. This is consistent with existing research that highlighted participants’ beliefs that their illness is so repellent that it was necessary to withdraw [14], with the unfortunate consequence of reducing self-esteem further and increasing social anxiety [33]. The reported success of Compassion Focused Therapy (CFT) (which focuses on reducing shame and increasing self-compassion) in reducing social marginalization [34] further indicates the significant impact of self stigma.

As well as comparing the self pre- and post-morbidly, we found that participants compared themselves to their (mentally healthy) peers and concluded that they had been ‘left behind’. The period of illness seemed to ‘pause’ progress through life stages, including education and training opportunities, romantic relationships, and having children. This is consistent with existing research that suggests a period of stagnation rather than a decline of existing function in the case of early-onset schizophrenia [35]. Several participants identified this as a contributory factor towards reduced social contacts, as meeting with old friends forced acknowledgement of stagnation and (in some cases) missed opportunities. Knight et al. [11] found that participants were unable to consider how their lives might have progressed without the illness because it was too painful.

The interviews raised several more idiosyncratic issues. Participants described peers becoming integrated in their experience of symptoms (for example, in hallucinations and delusions) and the loss of friendships as a result. Some participants became afraid of friends because they perceived them to be persecutory in some way and ended contact for self-preservation purposes, consistent with ideas regarding reducing stress [15, 16]. The consequences of such actions are mixed; while stress might be reduced, isolation is increased and social recovery becomes more difficult. Another mechanism was also apparent: participants were rejected by friends when accusations were made based on these hallucinations and delusions or when behaviour changed dramatically as a result of symptoms. In order to re-build these friendships it would be necessary to explain the behaviour by disclosing the experience of psychosis, which the majority of participants were reluctant to do. An emphasis on non-disclosure is also described by Green et al. [7]; participants with a wide range of mental health problems admitted that they were reluctant to disclose their illness because it might discredit them. McGlashen [36] described the possibilities for managing an experience of psychosis as either ‘sealing over’ the experience by not talking or reflecting upon it and looking forward instead, versus integrating the experience into the life story and making sense of it. While there is no evidence to say which strategy is more effective long term, there is some evidence to suggest that sealing over might help reduce negative emotional states in the early phases of psychosis [37].

Participants also described exiting friendships in order to avoid social drug and alcohol use. It seemed that participants didn’t feel able to return to these social circles without resuming previous habits and so recovery was prioritised. It has been widely documented that cannabis use increases the likelihood of psychotic relapse (for example, [38]). It has also been noted that service-users may be more likely to reduce cannabis use over the early months of treatment as readiness to change is especially high [39]. The relationship between substance use and friendships is complex especially when one party stops misusing substances. Kirke [40] carried out extensive peer network analysis and discovered that individuals made friends with people with similar substance misuse habits, but then the peer influence in the social circle maintained the substance misuse problem. Therefore, it is plausible that in order to break the cycle of misusing, maintaining factors (of which peers may be one) need to be removed.

When describing recovery, participants described a role for friends that was unique and could not be filled by family members. In particular participants highlighted pressure from family members and unsolicited advice regarding recovery. This is consistent with existing research that suggests that family relationships may be more intrusive (for example, [41, 42]) and may be complicated by feelings of guilt, shame and anger [43].

In terms of aiding recovery, participants made sense of their friends’ involvement as providing support and distraction. It seems that doing everyday activities (such as playing football) helped participants to re-build an identity outside of the illness, and the literature suggests that this is essential for recovery [18]. Five participants reported feeling ‘back to normal’ socially, and had re-joined their pre-morbid social groups. Stigma did not seem to impact on these pre-established friendships.

Consistent with other research (for example, [44]) two thirds of participants stressed an active intention to make friends despite the challenges this involved. Friendships with reciprocal roles and ego strengthening qualities were identified as important to ten participants, and this is consistent with literature regarding the formation of meaningful friendships [13]. These qualities were likely to be especially important to this population, as we know that individuals need to gain successes and pleasures in friendships in order to develop a sense of agency, vital for recovery [15], [45]). Participants also identified progress in other areas of their life as a way to improve social contacts, such as getting a job, accessing further education and having their own home. Pitt et al. [19] described a similar process of ‘active participation in life’ and its positive effect on social recovery, and several researchers have identified employment or training as an important step in improving social integration [20, 26, 42]).

The data suggested that service-led activities may play a valuable role in the initial part of social recovery for some individuals, in terms of instilling hope and a sense of belonging, consistent with Davidson’s [15] ideas. Participants made sense of this effect in terms of linking with individuals with shared experience and reducing the perceived sense of stigma. Chadwick [46] commented that service-led activities into the ‘real world’ are useful for widening outlook and making the transition from the patient sub-culture into everyday life.

However, there was some discrepancy both between and within participants’ accounts of the usefulness of service-led activities. This is consistent with findings by Knight et al. [11], who described a struggle between finding somewhere to belong and keeping oneself separate from a group that doesn’t have a positive identity. In addition, it is possible that service-led social activities are useful initially, but as symptoms improve participants need to move away from services to independently build social relationships. This allows them to move outside of the illness, which Deegan [18] suggests is essential for overall recovery. It also prevents psychosis becoming part of their identity, a risk of service-led activities previously identified [42].

### Limitations of the research

Discrepancies in the data were noticed, both between and within participants’ accounts. These were particularly stark in reference to disclosure of experiences to peers, and seem to highlight the difficulty participants themselves had in trying to make sense of their experiences. As the average time between referral to the service and the research interview was 19 months, it is likely that participants were still in this process, if they were attempting it at all. The time delay between referral and research interview was set at six months in the inclusion criteria in an attempt to manage this effect but it may have been unsatisfactory. However, it was necessary to balance this with the effect of the time delay on memory. Asking participants to recall pre-morbid situations, the acute phase of their illness and the subsequent recovery period was open to influence from the clarity of their memory and emotional state both at the recalled point and in the interview and so is a possible source of discrepancy.

The Duration of Untreated Psychosis (DUP) was not known for all participants due to omissions in the case files. Research suggests that the length of DUP is specifically related to social support, particularly at the time of presentation for treatment [47]. Furthermore, this has been proved to be independent of mediating factors such as a quicker approach to services when supported by friends and family [47, 48]. It would therefore be preferable to have gathered this information, possibly by participant-report if not recorded in client files.

### Future research

This study raises questions for future research, including investigation of the effect of stigma on established social relationships where self-stigma has been controlled. In addition, it was clear that participants exited friendships where drug and alcohol habits would be detrimental to recovery and this was in the context of the early stages of treatment and high levels of motivation to change (for example, [39]). Further investigation of this phenomenon would be beneficial; for example, could these decisions be made and followed through later in the course of the illness or is it this initial motivation burst that empowers? There is also scope to examine how the themes identified here are experienced by different groups in society; cultural background, age, gender and DUP may impact on social experiences following a first episode of psychosis and a mixed method approach with a larger sample would help to identify commonalities.

### Clinical and policy implications

This study along with existing research [3] indicates a need for a socially focused early intervention for people experiencing psychosis, because of the powerful impact interpersonal relationships can have on recovery [6]. Our findings suggest that this needs to be person-centered and responsive to the person’s stage in recovery and needs to reflect their understanding of the role of social relationships both within their illness and in their onward recovery. Therapeutic modalities and intervention programs focusing on cognition may be helpful to address self-stigma (for example, through the mechanism of increased self-compassion [34]), perception of strengths (which allow reciprocity in relationships) and social appraisals (for example, social appraisals in the moment have been shown to have a significant impact on loneliness [49]. The offering of service-led activities to share experiences seems to be a helpful and resource-realistic component.

## Conclusion

This study provides insights into the experiences of young people attending an early intervention service and highlights the difficulties moving from social exclusion to inclusion and recovery. Results suggest that individuals who do not manage to maintain established friendships as a result of consequences of psychosis may need extra support to enhance their sense of belonging, hope and agency. Further research would be beneficial to explore how best to achieve this, with development of strategies to support people in maintaining previous friendships and establishing new ones a potential need. Enhancement of peer support and of service provision by people with personal experience of psychosis and of recovery may be valuable. The data also suggests that support accessing training, employment and accommodation may increase opportunities for social successes and improved confidence.

## Supporting information

S1 File Transcripts of interviews. (ZIP)

S1 Appendix Flowchart illustrating the process of analysis and evaluation of saturation. (DOCX)

S2 Appendix Summary tables of initial codes. (DOCX)

S3 Appendix Interview schedule. (DOCX)
