# Supplementary material for: Experiences of friendships of young people with first-episode psychosis: A qualitative study
Source: PLoS One. 2021 Jul 30;16(7):e0255469. doi: 10.1371/journal.pone.0255469 (PMC8323937; doi:10.1371/journal.pone.0255469)
Supplement: S1 Appendix — (DOCX) [file pone.0255469.s002.docx]

**S1 Appendix: Flowchart illustrating the process of analysis and evaluation of saturation.**
