# Supplementary material for: Experiences of friendships of young people with first-episode psychosis: A qualitative study
Source: PLoS One. 2021 Jul 30;16(7):e0255469. doi: 10.1371/journal.pone.0255469 (PMC8323937; doi:10.1371/journal.pone.0255469)
Supplement: S2 Appendix — (DOCX) [file pone.0255469.s003.docx]

**S2 Appendix: Summary Tables of Initial Codes**

|  | **Code** | **Transcripts where it occurred** |
| --- | --- | --- |
| Friendships before illness | Always had few friends | 9,11, 12,14 |
| Description of current social networks | Few friends | 1,2,3,4,5, 9, 10, 11, 13, 14 |
|  | “proper” or “real” friends | 1,2,5, 10, 13 |
|  | Feeling lonely | 1,3,4, 11 |
|  | Family as friends / more important than friends | 2,3,9, 14 |
|  | Professionals described as friends | 4 |
|  | Romantic partner described as friend | 2, 14 |
| Maintenance of friendships | In touch with friends from school | 1,3, 12, 13 |
|  | Lost contact with friends from school | 2,3 |
|  | Life choice to lose contact with friend (eg, “bad influence” / drugs/ alcohol) / to aid recovery | 2,4, 9, 10, 11, 12, 13, 14 |
|  | Lost/reduced contact because of others judgement re: illness | 4, 9, 10, 12, 13, 14 |
|  | Lost (contact with) friends because of the of the symptoms (eg delusions, nightmares etc) | 4,5,9,10, 12, 13, 14 |
|  | Bullied as a result of symptoms | 4,9,10 |
| Social networks after illness | Low confidence socially during and after illness | 1,4, 9, 10, 13, 14 |
|  | Feeling back to normal socially | 1,5, 11 |
|  | Harder to manage conversations | 5, 9, 10, 13, 14 |
|  | Smaller groups easier to manage after illness | 1, 14 |
|  | Made friends as a result of treatment or intervention | 1,2,4, 11 |
|  | Socialising less since illness | 3,4,10 |
|  | Not talking about illness (afraid of others’ reaction) | 1,2,3,4, 9, 10, 11, 14 |
|  | Disclosing mental health but not psychosis | 1,4,5,10,11, 13 |
|  | Talked openly about illness | 5,9, 12, 14 |
|  | Shared understanding of illness helpful (eg groups) | 1,4,5,10,11, 13, 14 |
|  | Prefer to avoid service-led activities | 10, 14 |
|  | Chances to make friends are few | 2,11,9 |
|  | Miss friends that have been lost | 4, 13 |
|  | Social networks / skills better than before illness | 9,11 |
| Friends during recovery | Showed that they could be relied upon | 1,2,9,11, 12 |
|  | Became closer | 9, 12 |
|  | Everyday support | 1, 13 |
|  | Made [her] feel safe | 1,5 |
|  | Friends weren’t around / felt abandoned | 4, 13, 14 |
|  | Nothing friends could have done to help | 2 |
|  | Helped (or could have helped) by distracting from problems | 4, 11, 12, 13 |
|  | Friends helping more than family | 1,5, 12 |
| Changes they would like to make to social networks | Need more close friends | 1,9, 13 |
|  | Would like more friends | 1,2,4,10,11, 13 |
|  | Not interested in making new friends | 2,3,4,10, 12 |
|  | Would like a romantic relationship | 9, 4, 12, 13 |
| Important aspects of friends since illness | Trust | 1,3,5,9,10,11, 12, 13 |
|  | Friends who are a positive influence | 2, 14 |
|  | Friends to help discover new things | 1 |
|  | Friends of friends | 1,9, 12, 13 |
|  | Things in common | 1,3,11, 12 |
|  | Calm people | 1, 12 |
|  | Respectful people | 4,9 |
|  | Same nationality | 1, 13 |
|  | Able to contribute to the friendship | 1,2,4,5,10, 12, 14 |
|  | Intelligent | 2,9 |
|  | Low expectations / no pressure | 10 |
| What would help / helped to make friends / socialise | Feeling more confident | 4 |
|  | Life “back on track” | 4,5,10,11, 14 |
|  | Putting yourself “out there” | 5,9, 13, 14 |
|  | Talking to EIS staff | 1,2,4,9,11, 14 |
| Obstacles | Takes a lot of effort | 9,10, 14 |
|  | Don’t know how to make friends | 9 |
