# Supplementary material for: Experiences of friendships of young people with first-episode psychosis: A qualitative study
Source: PLoS One. 2021 Jul 30;16(7):e0255469. doi: 10.1371/journal.pone.0255469 (PMC8323937; doi:10.1371/journal.pone.0255469)
Supplement: S3 Appendix — (DOCX) [file pone.0255469.s004.docx]

**S3 Appendix: Interview Schedule**

**Interview Schedule**

**Before we start I would like to thank you for agreeing to meet with me. As you are aware, my name is Catherine Huckle and I am a Trainee Clinical Psychologist working in Camden and Islington. Today I would like to spend some time talking to you about your experiences of friendships in the last few years. There are no right or wrong answers – I’m just interested in understanding your personal experience. If you would rather not answer a question please just let me know.**

**During our interview it is important that I listen to you very carefully and so I am planning on recording the interview. Only I will listen to the recording and I will store it securely. It will be destroyed when the research is finished. Do you want to check anything before we begin?**

**I’m interested in hearing about people in your life who you see socially – people that you consider to be friends.**

**Present Friendships**

Do you feel you have friends in your life at the moment? Can you tell me about a friend that you have in your life at the moment?

*Possible prompts:* Where did you meet them? Did you meet them before or after your difficulties began? What sort of things do you do together? What do you like about them? What’s the best thing about having them around? What role do you play in these relationships? Can you describe a recent time that you did that with X? What sort of qualities would you look for in a friend / what sort of person? Are there other friendships in your life? What do you get from that friendship? Would you consider this person a confidante / trust them with sensitive or private information?

**Premorbid Friendships**

Can you tell me about a friendship that you had before the problems that bought you to the Early Intervention Service began?

*Possible prompts:* Have your friendships ever been different to how they are now? Have you lost any friendships as a result of your symptoms? How long ago do you think that was? Where did you meet them? What sort of things did you do together? What did you like about them? What role did you play in these relationships? Can you give me an example of something you enjoyed in these relationships? What happened to that friendship when your difficulties began? Do you think it was different to your current friendship in any way? What sort of qualities would you have looked for then? How would it feel to re-contact that friend? How have you maintained that friendship? What is it about that friendship that has helped it to survive over time?

**Process of change**

Do you think your difficulties have changed the way friendships are for you?

*Possible prompts:* why do you think your friendships are different? Do you have any ideas why that might be? Has the way in which you make friends changed? Do you look for the same or different qualities? Are you looking for the same sort of people to be your friend? Can you tell me about a time when you have noticed this? Are you looking for different kinds of friendships now? Do you do different social activities now?

**Friendships and recovery**

Do you think friendships have impacted on how you have coped with your difficulties?

What sort of support might have helped with regard to friendships? Has anything or anybody helped you with social relationships?

*Possible prompts:* who from? Tell me about the last time that happened.

**What’s missing..**

What would you like to change about your friendships now? Would you like more friends? What would you like to be doing with friends? Have you got any ideas about how that might happen? Do professionals ever talk to you about this? What could services do now for you in this area?

***General prompts:*** If life were better / you were satisfied with this area of your life, what sort of social contacts would you have? Is there something missing that you would like from friendships? Why? How? Can you tell me more about that? Tell me what you were thinking? How did you feel? Can you give me an example of that? What did that mean for you? Can you remember a specific memory of that? Do you get any help with this?
