# Supplementary material for: Experiences of friendships of young people with first-episode psychosis: A qualitative study
Source: PLoS One. 2021 Jul 30;16(7):e0255469. doi: 10.1371/journal.pone.0255469 (PMC8323937; doi:10.1371/journal.pone.0255469)
Supplement: S1 File — (ZIP) [file pone.0255469.s001.zip › Transcripts of interviews/Participant 1.doc]

| CH | Ok. So I’d like to start by hearing about people in your life who you see socially, ok, people you consider to be friends. Can you tell me about a friend that you have in your life at the moment? |  |
| --- | --- | --- |
| 1 | Well I don’t have a lot of friends, because I came here to [city] around 1 year and a half ago, and at this point I came with my girlfriend, who is my friend now. So she’s my best friend and the person who is like a family for me. And then I have a few friends who I met meanwhile. But there was always so busy at work and everything that I didn’t really have time to make a lot of friends. And these friends that I have apart from her are people who I met after my illness, so you can see in the last couple of months. One of them is [friend 1], and I met her at English course, which I attended when I was recovering, so it was nice to meet somebody new, and another person is [friend 2], he’s also [Eastern European], he’s my friend and now he’s also my boyfriend as well. Also, the other important person is [friend 3], she’s my flat mate and we are friends as well. So really there isn’t much people. I don’t count people who I know, like I wouldn’t call them friends, just people that I know, there’s plenty of people that I meet at the party, or somewhere around; they are not my close friends. |  |
| CH | Ok, so there’s 3 or 4 people that you’d consider good, close friends? |  |
| 1 | Yes, yes. |  |
| CH | What sort of things do you like to do with them? |  |
| 1 | With [friend 4] we go out to museums, or to the park. We like to go to [green open space] which is very close to us, we go to see the movies, and we also spend time at home together, because we are sharing a room, so we spend time at home also, and with [friend 1] I go out to see the concerts, music concerts, or just go out for a drink, and we’d, I say most of the time we just meet to speak, or we go out to cycle together. We both like bikes and cycling. |  |
| CH | So you do lots of activities, seeing things, and doing things together? |  |
| 1 | Yes. |  |
| CH | What’s the important bit about those friendships for you? Is it having somebody to do things with, or is it having somebody to talk to or somebody to listen, or giving some support? |  |
| 1 | I think it’s all of those things. Before I think that I.. Before my illness I was feeling a bit lonely because [friend 4] was my girlfriend but my friend. She was the only person I really knew here, so I didn’t have my other people, other people to speak about my relation with her, so I suppose I needed somewhere I could speak about my things, where we have things in common, but also I can support them, and we can, we have things we like to do together, and I don’t feel so lonely now because I have these people. |  |
| CH | Yeah, so they give you company, feeling that you belong, fit in with them and they’re like a family I suppose, because your family are a long way away, is that right? |  |
| 1 | Yes, yes definitely. |  |
| CH | Ok. Thinking back to before you were ill, before it was just you and [friend 4], is that right? |  |
| 1 | Yes |  |
| CH | Thinking back perhaps even before that, before you came to this country, did you have many friends? What were your friendships like then? |  |
| 1 | I used to have quite a lot of friends, most of them were friends from school, so all from high school and some friends I am meeting them still when I go to [Eastern European Country], so they are good friends. We go for.. Without seeing each other for a couple of months but we still meet, and after a while we still have things to speak about, it’s very nice. I’ve got some people from university, I have 2 or 3 really good friends from university so I have, like more than 4 people who are really good friends, 3 or 4 people I have in my life who are really important for me. |  |
| CH | So it’s a small, close group. |  |
| 1 | Yes, exactly. |  |
| CH | And your friends from home, the older friends, if you were to see them now, is that different since you’ve been ill or not? Is it the same as it’s always been, no change? |  |
| 1 | when I was, in January, in [Eastern European Country]… For example, it was just after I was ill, I didn’t want to see them because I didn’t feel confident. I didn’t want them to see any difference in me because at this point in my illness I behaved differently and I was peculiar with them. On the phone they said that I seemed a bit sad, a bit strange, so I didn’t want to see them then. But when I was in [Eastern European Country] about two months ago I met them and I don’t think there was any difference in our contacts because I came back to my old personality and I was the same. |  |
| CH | So in January when you didn’t want to see them it was because you felt that they would see something different about you? |  |
| 1 | Yes, and because I didn’t feel valuable. It was just after I was ill so I didn’t want to show myself in that state. You could say I wasn’t good yet, myself. |  |
| CH | That’s understandable. Thinking about [friend 4], because she was here before and during and after you were ill, is there anything different in that relationship? |  |
| 1 | Well, I think that our friendship became more strong, because she was with me at all times when I was ill in the hospital, everyday and later she was taking care of me so I think that now I know that I can rely on her at anytime, anything could happen, I know that she’s there for me. It’s really nice. |  |
| CH | It’s a very strong friendship? |  |
| 1 | Yes |  |
| CH | Ok, Do you think that now, after your illness, when you’re looking to make friends with people, do you look for the same things that you would have looked for before, or do you think it’s different things now? |  |
| 1 | I think that I’m always looking for the same things, because I always look for people that I can trust, rely on them, we have the same things in common. I’m not a very crazy person, I never look for people who are party people or something like this. I prefer more calm people and I think that this didn’t change to my illness, because the only thing I think that could have changed is that I need more close people, not like people who are just people who I know but I need somebody that I can trust. That is the only thing that might have changed, but then I always wanted people like that, so I think it hasn’t changed for me a lot. |  |
| CH | So trust has become a little bit more important but has always been important for you? |  |
| 1 | Yes |  |
| CH | Do you think having [friend 4] around impacted on how you coped with your illness? Do you think it made a difference to how you saw things? |  |
| 1 | Yes, because I if I didn’t have her, it would have been very difficult for me, I’m sure. First after I was ill I came to [Eastern European Country], I saw my family and they were taking care of me, but I don’t think it would be good if I didn’t have her here and I was staying in [Eastern European Country] so that my family could take care of me. It wouldn’t have been good for me because my environment and my life is here. I wouldn’t come back to my life, normal life so fast as I did when I came back here so I spent for one month in [Eastern European Country] and then I said to my mum that I want to go back to England because my life is here so I want to go back to doing things, start doing things so I came back here and at this point she was the only person with me. She was giving me support everyday and it was really important to me to become well enough. |  |
| CH | She was a constant for you? |  |
| 1 | Yes. |  |
| CH | And then when you came back, is that when you started your English classes? |  |
| 1 | Yes, a few weeks after this |  |
| CH | And how was that experience for you, going into a social environment with lots of people that you didn’t know? |  |
| 1 | Well the class wasn’t big, it was usually… If there are a lot of people around then I will get more quiet, but the group of 3 or 4 people it’s more easy for me to get more friends. The class was just me and three more people, so it was really good for me, yeah. Since the first day I became really good friends with [friend 1], we.. For example, we realized that we are reading the same books, that we like the same music, we like the same films, a lot of the same things, so we started to speak and we started to meet each other after classes so it wasn’t that difficult for me to get new friends in this class, because since the first day I met with [friend 1] who was my friend. |  |
| CH | And the other two people that you mentioned were friends, were they in your English class as well or were they from somewhere else? |  |
| 1 | Yeah they were in my class as well. |  |
| CH | And then as time went on they became friends? It started with [friend 1] as your close friend in the group, and how did the other friendships develop? |  |
| 1 | I don’t know. The rest of the people from the class they didn’t become friends. One girl wasn’t there very often and the other was a lady she was 50 years old so she wasn’t like me. |  |
| CH | There wasn’t much in common with her? |  |
| 1 | Yes exactly. |  |
| CH | And how about the other two friends that you mentioned, where did you meet them? |  |
| 1 | [friend 3], she’s a flat mate, she moved into our house. I don’t remember exactly when, it was about 2 or 3 months after I was ill. She’s [Eastern European] as well, the same nationality as me, just straight away it was much easier for us to get in touch, to speak about friendly things. She’s very talkative and easygoing person so we started to like each other. [friend 2], my [Eastern European] friend, I met him around 2 months ago at the picnic organized by [care coordinator], so it is very nice. There was some more friends [care coordinator] invited for this picnic and I met him there. |  |
| CH | And was it easy for you to start talking to him, somebody that you didn’t know? |  |
| 1 | Well, no it wasn’t that easy, also he’s [Eastern European] as well but I was a bit quiet at this party, this picnic, but because they, the rest of the people they saw that we somehow liked each other, they just left us alone so I was forced to have a talk with him. They went somewhere for a walk for one hour, we were staying alone, it was very nice. In this case we could see if we liked each other or not, and in the end we started to like each other, so it’s nice. |  |
| CH | Do you speak to any of these friends about what happened with your illness, or if you have any worries about what happened or anything like that? |  |
| 1 | I’m not speaking exactly what happened because I know that for the people it’s sometimes a bit scary, so I don’t want to scare them. I’m telling that I was ill and that I had a very hard time, because of that illness I had to leave my last job, but I never give much detail, I would say. |  |
| CH | Do they know that it was mental health problem or do they think it was a physical illness? |  |
| 1 | No, they think it was more like a hard time, they think it was depression or something. They don’t think it was anything physical problem. They know that I had problems and it was stress related; a mental health problem not a physical problem. |  |
| CH | Is talking to them about your illness and what happened, is that important to you? Or do you feel that you’d rather not talk about that with friends? |  |
| 1 | Apart from [friend 4] because she was there always, I prefer not to speak about the illness because I have her always to speak about how I feel and how it was and I can share everything. With other people I prefer not to speak about my illness. |  |
| CH | [friend 4] stuck by you through the illness and your friendship stayed really good from beginning to end, was there anybody that you lost touch with as a result of your illness? |  |
| 1 | No, because I didn’t have friends, anybody from here, there wasn’t anybody who I could. |  |
| CH | You stopped working, is that right? You had a job before. Did you go back to that afterwards? |  |
| 1 | No I didn’t, because my care coordinator we were thinking it was not good for me because my illness was job related, related to the job and the stress of the job so it was a decision we made. I need to find another job that will be less stressful so I didn’t come back to previous work. |  |
| CH | Was there anybody at your previous work who you were friends with or got on well with, or was it strictly a professional and work only situation? |  |
| 1 | It wasn’t a work only situation because we used to go out together on Friday nights or something, but it was like acquaintances not.. I didn’t really have any friends there. We were feeling well with these people, with each other, so it was a good environment. |  |
| CH | When you finished there, at that work, you didn’t see them or speak to them again? |  |
| 1 | No, I just saw them a few times because I had to go there to leave my doctors certificate. Sometimes when I was there we were like chatting, and I was going to visit them, but afterwards, after the hours of work I didn’t see them. |  |
| CH | Was that because you didn’t want to or because it didn’t seem to happen? |  |
| 1 | I don’t know. I didn’t feel like I would like to. |  |
| CH | Ok, that’s fair enough. |  |
| CH | Is there anything you’d like to change about your friendships at the moment? |  |
| 1 | Not really. The only thing I would like to change, I would like to have a bit more friends, because when [friend 4] is busy with someone, [friend 1] will go away somewhere, [friend 2] has to do something, and [friend 3] is busy and then I have a day off and I would like to spend the day with some of my friends but all of them are busy. Sometimes it happens that everybody is busy, so I would like maybe one or two more friends to be with, just to not be lonely. Sometimes I feel lonely. |  |
| CH | What sort of things would do you like to do with those two friends? Or if there was more people around? |  |
| 1 | If there was more people I would like to go to museums’, to the park, cycling, sometimes just to meet and speak would be nice. It doesn’t have to be exactly doing something, but I like to discover something about the other person, so if somebody likes, I don’t know, flowers, and would like to go to Kew gardens, I’m happy with it. It’s always nice to discover new people, very nice. |  |
| CH | Have you got any ideas about how you might make some new friends? |  |
| 1 | Last time I was in BBQ parties at the weekend, it’s a good way to find new people. If somebody invites me for some party, I should go there because I don’t like to meet people.. To go especially to the pub or to the club to make friends, its not a good idea. I like to look in the circle of friends that I have already to look for new friends is better. |  |
| CH | Why is that do you think? |  |
| 1 | I don’t know, I think that I’m not so open and confident and brave just to go out alone to look for some new people. If your phone rings and someone introduces you to someone, this is Kate, this is Maria, and then it’s easier for me to meet this person I think. |  |
| CH | So the support of your friends being there should make it easier? |  |
| 1 | Yes. |  |
| CH | Is there anything about meeting people who are already friends of a friend that makes you think they would be a good friend rather than a stranger perhaps? |  |
| 1 | Umm |  |
| CH | Does that make sense? |  |
| 1 | I’m not sure what you’re saying. |  |
| CH | Let me say it again. |  |
| CH | I guess I’m wondering if somebody is a friend of a friend if there’s something good about them that will mean that you’ll like them as well? |  |
| 1 | I know what you mean. Somebody who is in my circle of friends who is not my friend yet but I would like him to be my friend, why.. I don’t know, I think that I used to look at people if we have things in common, or of course if this person is nice usually the person is nice too. But I’m looking at how I speak to this person, what we speak about, if I like the way the person is then, yeah it’s easier to become friends. But then I have to see something I like in this person to become friends. I think that sometimes its things in common but sometimes it will be somebody who likes really opposite things because it’s something different, so I don’t have something in particular that I think should be the case. |  |
| CH | Ok. Does anybody at the EIS ever talk to you about friendships? |  |
| 1 | Yes, I mean I speak with [care coordinator] about the things about my friendships. |  |
| CH | If you had any worries about your friends or friendships, would you talk to [care coordinator]? |  |
| 1 | Yes, last time was yesterday |  |
| CH | Is there anything that services here could do to help with friendships, do you think? Anything to help you with it? |  |
| 1 | I think that, yeah, [care coordinator] is the person that I can speak with about everything. I am honest with her about my problems so my worries and everything. Its helpful that I can speak to her if I have some worries related to my friends as well. I can speak all of it to her, so I think its very important because its, she’s not like from the circle of my friends or my family but she’s someone who’s outside who can say or who can just listen to me. It’s very important to have someone to speak to about important things. I think it’s just helpful. |  |
| CH | So aside from [care coordinator] listening and talking, is there anything that you’d want the service to do or organize or provide for you with regard to friendship? |  |
| 1 | Maybe some more… It would be nice if there were some more groups to meet people from EIS or the people we can meet here. For example, I go badminton group, its someplace where I can meet people. Yesterday there was only Marjorie and me but usually there’s more people, but if there’s some more groups then we can meet and we can go on it would be nice. But anyway now I’m working so I don’t have loads of time for this but for other people who have more time, for other clients of the EIS I think that it would be nice. |  |
| CH | Some people want to make friends who have also been here, and some people don’t. What’s your position on that? |  |
| 1 | I don’t mind, its nice because we were almost in the same situation so I don’t have to hide anything so like with my friends I don’t like to speak about this but with people form EIS I can ask or say something about how did I feel or how did they feel so its nice to have people who were ill with the same thing like me. |  |
| CH | The same experience? |  |
| 1 | The same experience, yes. |  |
| CH | Thinking about [friend 4] being there for you when you were unwell, can you give me an example of when having a friend around really helped you? |  |
| 1 | I mean.. For example, when I was in the hospital I was feeling a bit bad, unsafe and in the beginning especially. It was important that she was there everyday to see me, so someone to speak and to hug. |  |
| CH | Someone who you could trust. [ptp crying] It seems difficult for you to remember that time. It was a hard time for you. Talking about it now shows how far you’ve come doesn’t it? |  |
| 1 | Yes, yes. |  |
| CH | Let me pass you a tissue |  |
| 1 | Thank you. |  |
| CH | Which bit is making you feel upset? Remembering the hospital or thinking about [friend 4] helping you? |  |
| 1 | Both. Its sad that it was so bad, it was very strong emotions, and even now when I remember it makes me feel a lot, and I feel a lot for her, that she did for me all these things, it was very important for me. |  |
| CH | What she gave to you? |  |
| 1 | Yes. As a friend and as a part of my family. She’s like a family for me. |  |
| CH | And you’ve been through a very difficult experience together.  Do you think there’s anything that’s missing in your friendships that you have at the moment or are you happy that everything that you need is provided for in your friendships? |  |
| 1 | Well firstly with [friend 4] there is nothing missing for sure. But with the other people I don’t know, they are my friends but I’m not sure if I could speak about everything with them or if I could rely on them always like with [friend 4]. They are good friends and I like to go out with them and speak and everything but I’m not sure if they would be there if I was ill again. I’m not sure about this. I don’t know if they are such good friends. There may be something missing, because if somebody is a really good friend they would be there for you and I don’t know what would happen. Maybe I know them for too short time to know that. Hopefully I will not have to realize that part of the friendship. |  |
| CH | To be able to talk about what happened to you and the difficult time that you had, do you think you would need to know somebody for a long time and know them very, very well? |  |
| 1 | I think so. In my case to open myself to another person I need to know them much better, yes. I can go for walks and have fun, but I need to feel that I can trust this person and I can tell her or him everything. If I don’t feel like this I will not speak about this. |  |
| CH | Have you always been like that since you were a child or is that new? |  |
| 1 | I think that somehow I was always like that because in the childhood I was ill for epilepsy and none of my friends knew about this, so I think I was like that before as well. |  |
| CH | So you’d keep some of that to yourself rather than talking about it with anybody around you? |  |
| 1 | Yeah. |  |
| CH | Another question about [friend 4] really; I’m wondering what it is about the friendship that helped it to be such a good and strong friendship? |  |
| 1 | What was so good? |  |
| CH | Yes, what was it about you and [friend 4] and the two of you together that has meant it has survived? |  |
| 1 | I don’t know, I think its because, firstly, we were in love with each other so we even though for now we are not in love, we love each other like a family. This is very important thing. If you love someone you can be there for him or for her and also the other thing is that at the beginning in London we didn’t have anybody else so I just had her and she just had me. Now it has changed because I have some friends and she has someone important; friends in her life as well. Because we were so attached to each other our friendship was, from the beginning, very strong. And also they must make this stronger, the friendships. |  |
| CH | Thinking about the people that you lost contact with who you were more acquaintances with, what would it feel like to get back in touch with them now? Would that be hard to do or would it be that you didn’t want to do it? |  |
| 1 | I don’t really want to do it, I think. We were acquaintances but I didn’t feel like that I would like to spend much out of work time with these people even when I was feeling well, when I wasn’t sick, so they were nice people to drink a drink on Friday evening after work but nothing more. I wouldn’t like to get back with them now. |  |
| CH | So they weren’t really people that you would pursue more time with? |  |
| 1 | No, no. |  |
|  | And with your friendships that you have at the moment, the friendships that you’ve described with [friend 4], [friend 1].. I’ve forgotten the names of the two other friends? |  |
| 1 | [friend 3] and [friend 2] |  |
| CH | What sort of role do you think play in these friendships? What’s your part of the friendships? |  |
| 1 | I don’t know, what kind of roles can there be? |  |
| CH | For example, some people say that they are the one who listens, or they are the one who speaks, or they are the one who organizes things, or they’re the one who looks after somebody.. Do any of those roles resonate for you? |  |
| 1 | For [friend 4] its like we both look after each other. I look after her about everyday things like a meal or some housework or something, but she’s very, not well organized person so very often I have to organize something for her. For example, she lost something last week, her bank card, so because her English is not good like mine so I called for the bank for her to cancel the card. For her its really difficult and she really scared of it, but for me is it nothing to do this, but for her it was really important, so I am the organizer as well. With [friend 1] she is more like the organizer because she likes to organize parties or to go out with people somewhere and I just ask her what we can do and then she says ‘come on lets go there’ or lets go here’. And also playing the part to listen as well as to speak with her. With [friend 3] it’s more like we don’t speak about deep things I would say, we just like to speak about everything and nothing I would say. It’s a part that is enjoying speaking and listening to the other person. With [friend 2] sometimes I’m organizer as well, so I’m giving ideas what we can do, where we can go and also if he needs me to help with something I can give him help in some kinds of things. |  |
| CH | Is there anything that he helps you with? |  |
| 1 | That’s a good question. He didn’t help actually; he hasn’t had a chance yet if he can help me with something. I think so. |  |
| CH | It’s a new friendship. |  |
| 1 | Yes, I think I haven’t realized about that before. |  |
| CH | Is there anything else you can think of that you would like to say about your friendships; how they are now or how they were or how they might have been different or changed? |  |
|  | I don’t know. I think I have been happy with my friendships for now, but I think that I would like to have 1 or 2 more friends, that’s the only thing. But these people that I have now I am happy with them. |  |
| CH | Ok, thank you. |  |
