# Supplementary material for: Experiences of friendships of young people with first-episode psychosis: A qualitative study
Source: PLoS One. 2021 Jul 30;16(7):e0255469. doi: 10.1371/journal.pone.0255469 (PMC8323937; doi:10.1371/journal.pone.0255469)
Supplement: S1 File — (ZIP) [file pone.0255469.s001.zip › Transcripts of interviews/Participant 10.doc]

| CH | I’m interested in hearing about people in your life who you see socially, people you consider to be friends. Is there anybody who comes to mind for you at the moment? |  |
| --- | --- | --- |
| 10 | I used to have a friend before I fell ill, a few friends, but I don’t see most of them now. I just speak to neighbours sometimes and family. |  |
| CH | These friends that you used to have, what sort of things did you use to do with them? |  |
| 10 | They used to visit me, we used to go out, exchange presents, talk about families and things. |  |
| CH | How did you meet those friends? |  |
| 10 | I had known one since I was a teenager, she used to be.. We met.. Living with me and my mum, so she went back home and then came here and got married, and then we met again. For a long time we’ve known each other. |  |
| CH | Was that the same for the other friends as well? |  |
| 10 | I think lost them before I fell ill, or I was already ill but not diagnosed. I just.. I was not nice or polite to them anymore so we lost contact. I changed my number. |  |
| CH | At what part did you think those friendships fizzled out? |  |
| 10 | I think I was feeling under too much pressure with things, and then I couldn’t cope with having to live up to these friendships. |  |
| CH | So there was something about not feeling able to do something with them? |  |
| 10 | Yeah. Or thinking that I don’t have time to do things with them. I felt under pressure. |  |
| CH | And then when you started to feel better, did you start to see them again? |  |
| 10 | I called one of them, she said that she was going to come to see me and then I said ok, I’ll call you again to arrange a date, but then I didn’t get back to her because I don’t want her to come. |  |
| CH | Why’s that? |  |
| 10 | Because she’s going to ask me questions and I’m just going to be under pressure. She was going to bring her little girl, and I just felt under pressure. |  |
| CH | What was it that she might ask questions about? |  |
| 10 | About my health. |  |
| CH | Would you want to talk about that to her? |  |
| 10 | No. She’s a qualified nurse though. I told her a little bit on the phone, and she already knew that my brother had it so she was alright with it, but I just don’t want to talk more about it. |  |
| CH | How about the other friends that you had before? Did you have any contact with them after your symptoms? |  |
| 10 | No. One of them had a baby and I was going to approach her, but then I changed my mind, so I didn’t talk to her. |  |
| CH | What made you change your mind? |  |
| 10 | I thought I was being bullied by her, but I probably was, because I was very weak when I was ill. Then I thought that she may start coming round to my house again and that could start again so I didn’t. |  |
| CH | When you say being bullied by her, what sort of things was she doing? |  |
| 10 | She sort of took over my home, and was staying there very long hours, all day and all night; bringing friends in and I had to go upstairs with my daughter. She took the downstairs part. Drinking, doing drugs and things like that. |  |
| CH | You decided not to contact her because you didn’t want this situation to come back. Was there anybody who was around for the whole time that you were feeling unwell, or around still when you started to feel better? |  |
| 10 | No. There were lots of people that I was avoiding, so no one was basically around. A few neighbours, but they were also avoiding. |  |
| CH | It sounds like when you became ill, people were avoiding you – friends and neighbours? |  |
| 10 | Yeah. |  |
| CH | Was there anybody that you talked to during that time? |  |
| 10 | Just my family. |  |
| CH | Are you close to your family? |  |
| 10 | Yes. |  |
| CH | If you were going to talk to somebody about what you’d been through, if you were feeling worried or frightened… |  |
| 10 | I would talk to my family. |  |
| CH | I’m just thinking about this friend who you thought you might contact but then you decided not to because of the pressure. Can you tell me a little bit about that pressure? |  |
| 10 | About the one who was going to come to my house? |  |
| CH | The other friend, the one who you thought you might call; that you were going to call back with a date. |  |
| 10 | I just felt that she’s going to come and my house won’t be tidy enough. It’s too many little things. Food should be ready, and I won’t be able to manage, and she will ask me too many questions, whys and things and I might not be able to cope with it. So I didn’t want her to come. But I will maybe. |  |
| CH | What would have to happen to make it easier to call her? |  |
| 10 | I think, for a while, we should just speak on the phone and she shouldn’t say that she will be coming. |  |
| CH | So it’s nice to talk to her, but the thought of her coming to your house is difficult? |  |
| 10 | Yes. |  |
| CH | How would you feel about going to her house? |  |
| 10 | I won’t like it. It’s too far. It’s in Brighton and I will have a problem travelling over to her, and then I don’t think I would feel comfortable. |  |
| CH | Have you done that journey to Brighton before? |  |
| 10 | Once, when she had a baby. I went with my brother to see the baby. |  |
| CH | I’m just wondering if travelling is harder since you’ve been unwell? |  |
| 10 | Yes, it’s really hard, and if the environment.. If it’s different then it’s quite uncomfortable. |  |
| CH | So you prefer to be at home with your own things? |  |
| 10 | Yes. |  |
| CH | Do you think it would have helped if there had been a friend around when you were having your symptoms and feeling unwell? |  |
| 10 | I think the symptoms were quite.. Only a doctor would understand; not a normal person would understand it. Even I couldn’t understand it when I was ill. The doctors kept on saying that you’re sick, but I didn’t believe them, but then when they gave me medicine and things started to getting together after a few weeks, and I thought ‘oh, I must have been’. So the symptoms were only a doctor or nurse would understand, not normal people. |  |
| CH | So if there had been somebody around they might not have understood anyway? |  |
| 10 | No. My husband was saying that she’s ill, to my family, but my family didn’t believe him. I looked really bad. I lost a lot of weight but they thought it was good because I was big before. |  |
| CH | Your husband was somebody who was around who was quite supportive during this time? |  |
| 10 | No, because I used to tell him to go away because I was frightened of him, so we separated and he moved. I accused him of doing things which I believed he did, but they weren’t true, I was just feeling that. And then he kept saying that she’s not well, what’s she saying, and my family didn’t.. Some parts they probably did think but not all. |  |
| CH | When you started feeling better, were you able to speak with your husband about what happened? |  |
| 10 | Yeah, I spoke with him but I explained to him.. But not everything what I said because it’s a bit painful. He said it’s ok, don’t worry about it, but still it’s quite difficult being together after all the allegations I put on him. It’s quite difficult at the moment. He’s ok, he understands; some things he understands but not all of it. |  |
| CH | The two of you are working to see if you can put things right? |  |
| 10 | Yes. |  |
| CH | Which bits do you think he doesn’t understand? |  |
| 10 | My sleeping pattern’s a bit weird. He doesn’t understand it, and sometimes he’s nice and some days he expects more than I can do. I don’t like the idea, because it’s very difficult for me to manage. Some days he’s quite helpful towards my daughter and some days he’s not helpful at all. He says ‘lets go on holiday’ or something, but then when we go on holiday I don’t have enough energy to hang around a lot, and he doesn’t understand that. |  |
| CH | It sounds like the pressure that you described when people come to your house - people expecting you to do more than you’re feeling up to, is that right? |  |
| 10 | Yeah. I feel pressure with him as well. He’s not with me at the moment, so he visits me regularly. I feel the pressure but if were living with me I would feel loads of pressure. He would expect his clothes to be washed, ironed, the house should be tidy and clean like a normal house, like I used to do everything. But now I can’t do everything. I can see and understand that this needs to be done but motivation is quite low, and concentration as well. You know why I was late today? Because I fell asleep. I took painkillers. I have toothache, and I fell asleep. |  |
| CH | I’m sorry to hear that. Is it still sore now? |  |
| 10 | Yes, I have to go to dentist after this. |  |
| CH | Let know if you need to take a break. |  |
| 10 | No, I’m okay. |  |
| CH | I’m just thinking about having some new friends in your life. How do you feel about the idea of making new friends? |  |
| 10 | I wouldn’t like the idea because it’s very hard to trust people now. I’ve made one new friend, she’s from [Asian country] and not a very close friend, but she comes to my house sometimes and we’ll talk a little bit. Sometimes we tidy up together, not a lot, but a little bit. She’s ok. That’s the only person I’ve made friends with, sort of. She actually rang me yesterday saying that her father passed away, so she was quite distressed, so I think that the friendship with her is quite good, but not very close. She’s welcome to come to my house and sit, but she doesn’t expect too much from me because I told her that I’m not too well. So she’s ok. |  |
| CH | This friendship feels a bit more manageable because she hasn’t got too high expectations? |  |
| 10 | Yes. |  |
| CH | How did you meet this friend? |  |
| 10 | I went to buy some clothes from her shop. Her shop is just round the corner from my house and she sees me dropping my daughter every morning, and picking her up every afternoon, so she’s been seeing me for a couple of years. And then I went to buy some clothes from her shop, a few times, and then we started talking and I said if you like you can come to my house. |  |
| CH | You made an approach to her to become your friend and she took you up on it? |  |
| 10 | Yes. |  |
| CH | Was that easy or was that hard? |  |
| 10 | It was very hard. It took me about six months to think about it and then I really wanted to speak to her, but it took me six months. |  |
| CH | It was very brave. I’m just thinking about your new friend; did you look for different qualities in your new friend to what you might have wanted before? |  |
| 10 | My mum was looking for help with her home, personal help, and I thought.. That’s how I approached her. I said ‘look, Mum’s looking for help, so if you know someone, I see you in the shop all the time so if you want to come to my house, it’s good’. And then she came and we spoke a bit more about my mum, and then she sometimes - once or twice a month - she goes to my mum’s in [local area] and spends some time with her as well. She’s a nice girl. She’s 26. Sometimes she comes to my house as well. We were meant to meet on Sunday. She doesn’t smoke or anything like that, so she’s quite different to what I am but she is quite okay with everything. We were meant to meet on Sunday but my uncle died so I had to go away, so I phoned her and said I can’t meet with you this week, maybe next week. |  |
| CH | I wonder what it is about her that makes her a good person to be around, or easier to be around than other people? |  |
| 10 | I think I feel safe. Even my money and my jewellery’s open and I don’t feel worried. Some relatives are coming from [Asian country] to stay with me in December and I’m not happy about them, I’m very tense. Every time I go in the bathroom I think I have to move my things from here, and from my living room and things. I have to move my things because if something goes missing I can’t say anything because they are relatives, so.. It’s lots of burden and I hate it. I lay in bed thinking about how I’m going to manage because its four adults and three children, it’s quite a lot. The children are small. My house has hazards as well for smaller children, not for adult children but like cupboards and pushing things and falling and things like that. I have to sort things out before they come. It’s too much pressure, and arranging the space for them to sleep, and get a new mattress. |  |
| CH | How will that be, being around other people so much? |  |
| 10 | Yeah, and my house is upstairs and downstairs but there won’t be any privacy, and that’s why I spend most of my time in the kitchen and they can be there. I don’t know how I’ll cope. I spoke to my husband’s sister about I’m not happy with this. I’m not too well to cope with such a big pressure, and she was not understanding at all. She said ‘look, you married into our family and you haven’t done anything for ten years’. I said ‘I can’t really’; she said ‘look its only 4 or 5 days, you should do this’. But I don’t think it’s going to be 4 or 5 days, I think it’s going to be longer. |  |
| CH | How about making conversation, talking and being around people socially, what’s that like for you at the moment? |  |
| 10 | It’s really hard. I don’t mind speaking to professionals like you or the dentist, because they understand me and support me, but I can’t speak to everyone about what’s going on in my life or how I’m doing. I’m quite.. Hiding things. |  |
| CH | Do you manage to talk to people about other things? To distract yourself or is that hard? |  |
| 10 | I don’t approach them, but if somebody approaches me; ‘yeah it’s a nice day’ or something general, basic things, but not much. |  |
| CH | Thinking about your new friend, the lady from the shop, is the sort of things that you would do with her different to the friends that you had before? |  |
| 10 | Yes. She’s quite down to earth but my other friends were very posh-posh. It’s quite hard to cope with that. |  |
| CH | Which bit was hard – the down to earth bit or the posh-posh bit? |  |
| 10 | The posh bit, because I used to be like them before but not anymore. I’ve changed quite a lot so it’s very hard to fit in with their expectations. There’s a lot of difference between my friends and her. |  |
| CH | If you were looking to make some more new friends, do you have any ideas how you would do that? |  |
| 10 | No, because I’m not going out anywhere and it’s very hard to trust people, especially if they’re not in the field of a professional. |  |
| CH | Are there any particular qualities that you would want in a new friend? What would you want them to be like? |  |
| 10 | Polite and understanding, and not expecting a lot from me. But everybody sort of expects quite a lot so I feel a bit of pressure. |  |
| CH | I’m just thinking about your current friendships.. |  |
| 10 | I talk to neighbours, and they will sometimes come to my house but not for long, just for a few minutes, sometimes to drop a child to play with my daughter, and when I had a party for my daughter. But I didn’t speak to them then, I was serving food out in the kitchen mostly. |  |
| CH | What would need to be different for you to call those neighbours friends rather than neighbours? |  |
| 10 | Because they’re… They’re not my friendships, they’re mostly my daughter’s. We only speak because the children speak and go to each others’ house. Adults don’t come much, just children. I try not to have too many children in my house, just one at a time. |  |
| CH | Would you want any of these neighbours to become closer friends? |  |
| 10 | I think no, because where I live is like the green in the middle and then there’s flats, flats, flats and then so much gossip. Everybody knows everybody’s business. |  |
| CH | You would be worried that the neighbours would talk about your business? |  |
| 10 | Yeah. One of my neighbours recently approached my mum and said ‘how’s your daughter, she’s been really depressed for the past few months’. My Mum said ‘she’s fine’. I think that the crisis team was coming and they saw the NHS car so they found out. |  |
| CH | How do you feel about that? People knowing what happened to you? |  |
| 10 | Not happy, not nice, because there’s quite a lot of rumours and things like that. |  |
| CH | What would you be worried that people are thinking or saying? |  |
| 10 | Like I’m mad and things like that. |  |
| CH | Is that the same reason that you don’t talk about your symptoms with anyone but your family? |  |
| 10 | Yeah. |  |
| CH | In case people think you’re mad? |  |
| 10 | Yeah, or that I have a behaviour problem or something like that, because that affects my daughter as well then. People will keep a distance from her as well, and their children, so it’s not fair. |  |
| CH | Some of keeping yourself to yourself is to protect your daughter? |  |
| 10 | Yeah. |  |
| CH | I’m just thinking about your friend who you were talking about, who you said you would ring back.. What’s going to need to change before you feel ok to make that phone call? |  |
| 10 | The pressure has to reduce. I’m really pressured about the family coming from back home, so that has to go. I have to be a bit more free in my daily responsibilities. My sleeping habits should be a bit down. It’s quite a lot to manage at the moment. |  |
| CH | So there will be less pressure when the family visit is over with, when you’re feeling a bit more rested perhaps, sleep may be more back to normal, everyday chores that have got to be done will feel more manageable? |  |
| 10 | It’s getting there slowly. Before, my house was very dirty and messy, but slowly, slowly it’s getting there. It looks clean but the cupboards are very untidy. |  |
| CH | I remember you saying that your friend from the shop had helped you tidy round sometimes. |  |
| 10 | Yes, she helped me tidy sometimes. She likes my cat, and she likes my daughter as well. Not too much, I don’t like her to do too much, but if she likes to do something I tell her. If we are cooking together she does the washing up while I am cooking, or if we eat together then she cleans the table and helps me take the dishes back, just little things. She also helps my mum but she gets paid for that. |  |
| CH | But she doesn’t get paid to help you? |  |
| 10 | No. |  |
| CH | It sounds like your new friend is more supportive and helping you to take care of things that old friends would be? |  |
| 10 | Yeah. We used to be like eat, make mess, and that’s it. |  |
| CH | By having someone who helps out that reduces the pressure a bit in what they expect when they come? |  |
| 10 | Yeah. She won’t come very often, once or twice a month for an hour or maybe two - that’s good enough. If I really want help and I want to pay her maybe she would accept it but I didn’t talk about it. |  |
| CH | In a friendship, what sort of thing you would like to do for your friend? Do you like to be somebody who is the joker, makes them laugh, or is the listener, or is the one with advice or..? |  |
| 10 | I think I would be a good listener, I wouldn’t advise because I don’t want them to think that I’m judging. I would help with physical chores. When my uncle died, when I went to my sister we all had to do chores and things because they had all the guests to come. It was mostly her family but I helped her to arrange it and food for 25 people; pouring food into big bowls and I just served it. |  |
| CH | So you’re very good at helping with practical jobs? |  |
| 10 | Yeah. Especially when my mum tells me. |  |
| CH | How about friends for you – which bits do you like in them for you? |  |
| 10 | Just support actually. I don’t expect them to help me a lot unless they created the mess, but is supportive and polite. |  |
| CH | Is there anything you’d like to change about the friendships that you’ve got at the moment? |  |
| 10 | Not with friendships, but with my family I would like for them to come to my house a bit less, because everyday somebody’s there, and that’s quite.. Not good, but my daughter likes it so I can’t really complain much. When they are not there she is more lonely and she keeps on saying that she wants to go to her friend’s house, and then sometimes I call her friend over, and sometimes I send her. |  |
| CH | So one thing you’d like is less family members popping in all the time? |  |
| 10 | Yeah. All of them come together; it’s extremely difficult. I physically feel so tired, and mentally as well, but it starts with mental tiredness and then start getting aches and pains and then I’m sleeping very deeply for 14 hours or something. |  |
| CH | Would you like to have more friends about, or does that not feel manageable at the moment? |  |
| 10 | I think I would like to have more friends to go out with, shopping. But then I do my shopping online since I fell ill, and so it’s not that I need to buy things, but sometimes I go to [shopping area], like once a year or something, but if I had somebody to go with I would probably go a bit more often. |  |
| CH | Shopping is a good social activity isn’t it? |  |
| 10 | Yes. I’m doing it online for weekly things, for my daughter mostly. She always needs clothes because she grows up in size. |  |
| CH | Ok, so you’d like friends to be around to do more things like shopping. Is there anything else you’d like to be doing with friends? |  |
| 10 | I’d like to go to [supermarket], for doing my home shopping, but my brother takes me, so that’s quite good. If he wouldn’t take me I wouldn’t go, but before I wasn’t going at all, they were bringing me the shopping in, but now he takes me. |  |
| CH | What is about [supermarket] that makes it difficult without your brother? |  |
| 10 | To carry the things, and to watch my daughter because she’s going to run around everywhere and I’m so scared of losing her. |  |
| CH | Keeping your mind on all these things at once is tricky. Does anybody here ever talk to you about friendships? |  |
| 10 | No, no one’s ever spoken to me. |  |
| CH | Is there anything you would like the service here to do to help with social things? |  |
| 10 | They offered me about some groups, activities and things like that, but I don’t want to because then everybody knows your condition. I didn’t like, want to go to it. |  |
| CH | Tell me about that a little bit more. The groups would be everybody who comes here, they’d have a similar condition. What is it that would worry you about them knowing? |  |
| 10 | Even if they have a similar condition I feel like.. Not too good for them to know I have the same condition as them. In a way it sort of relaxes you as well to know you’re not the only one. |  |
| CH | So it’s a sort of a pro and a con from coming to the groups here; a good thing might be that you wouldn’t be the only one, but a con might be that people would know that about you. |  |
| 10 | Yes. I would be really worried about someone seeing me who was from my area and then they would gossip, and then I don’t want people to keep away from my daughter. |  |
| CH | I can understand why you would worry about that. |  |
| 10 | And if life was really good, exactly as you wanted it, would you be seeing more friends, or the same? |  |
| 10 | Maybe more. If I was healthy I would probably still be with the friends who I’ve been avoiding. I won’t be like smoking cannabis again but I did sometimes smoke before. I think I shouldn’t be doing that, it’s not a good idea, I realise that. It’s quite bad in the long run, even financially and being around for the children. |  |
| CH | Is that one of the reasons that you don’t see them, because you don’t want to smoke anymore? |  |
| 10 | Yeah. |  |
| CH | So if you saw them there would be a chance that you would end up smoking? |  |
| 10 | Yeah, if it’s in front of you, you can just be very intimidated. |  |
| CH | So one of the reasons is that you choose to avoid that situation? |  |
| 10 | Yeah, they are encouraging you so.. Before this I wasn’t smoking that before I met them, for many years. |  |
| CH | Is there anything else that you’d want to talk about in relation to friendships or socialising that we haven’t touched on? |  |
| 10 | Not really, no. |  |
| CH | Thank you. |  |
