# Supplementary material for: Experiences of friendships of young people with first-episode psychosis: A qualitative study
Source: PLoS One. 2021 Jul 30;16(7):e0255469. doi: 10.1371/journal.pone.0255469 (PMC8323937; doi:10.1371/journal.pone.0255469)
Supplement: S1 File — (ZIP) [file pone.0255469.s001.zip › Transcripts of interviews/Participant 11.doc]

| CH | I’m interested in hearing about people in your life who you consider to be friends, or who you do social stuff with. Is there anybody like that at the moment? |  |
| --- | --- | --- |
| 11 | Yes, there is. |  |
| CH | Can you tell me about them? |  |
| 11 | Basically, I met them in college. I met a couple of people at the EIS groups. I’ve got to know a couple of them, got their numbers and met up and stuff like that, socialised basically. I usually pick my friends. I’m a person who’s.. Not too picky, but one or two rather than having five or six. I like to explain things to them, like what I done or anything like that. |  |
| CH | Lets talk about the friends from college a little bit first then. How long have you known them? |  |
| 11 | Probably like four or five weeks, four weeks now. |  |
| CH | Quite new friends? |  |
| 11 | Yeah. I’m actually going out with one girl after this. Yeah, I’ve got to know her really well. |  |
| CH | These people from college, are they in your class or..? |  |
| 11 | Yes, they’re mainly in my class. Other departments.. I haven’t met people from other departments, so I’ve not… Personally, I’ve not interacted with other people. The other people are not from my course, so if there was someone from biology I probably wouldn’t relate to them and stuff like that. |  |
| CH | These people that you’ve recently met from college, what sort of things would you do with them? |  |
| 11 | We’re thinking of going paint-balling as a group; with the girl, we’re going to go out today, and usually we talk, go out for lunch and stuff like that, go for a drink, or for a coffee, whatever. Stuff like play football or play badminton together. |  |
| CH | Are there any friends in your life at the moment who were around before you became unwell? |  |
| 11 | Yes, there’s one. There’s one. I used to go to university, and I’ve got a university friend. We went on holiday to Barcelona a couple of days back for four days. And he’s a really close friend. Basically nobody knew what I had apart from my family: my mum and dad. Everyone else I hadn’t told, and I’d like to keep it that way. I don’t like talking to other people about my illness. I think people get the wrong idea, they just walk away, they don’t listen, they don’t.. You know, they think there’s something wrong with you and you’re mentally unwell and stuff like that. The thing is, they think that I might be harmful in society and stuff like that, but I’m not. I’m not a violent person. I’ve never acted that way or talked that way to anyone before. It’s not, it’s not.. People just think differently – what you did wrong, or are you taking drugs, but it’s nothing like that. You can’t… You can’t talk to people about this sort of stuff with people. It’s really confidential for me, really difficult.. I don’t like talking about it and when I do I get really upset and stuff like that so I don’t like talking to people, even friends, about what happened, what I went through. So it’s secret. The easiest way is that I had depression, and you know.. Apart from that, nobody else knows what happened. Its just depression. I wasn’t saying psychosis or anything. |  |
| CH | This friend, what do you think it was about him that meant you guys have stayed in touch, stayed good friends? |  |
| 11 | He’s a good friend, a good mate. He’s like.. He’s not like penny counting, those kind of people. He doesn’t slap on your face a joke that’s really bad. Someone you can talk to. He can be funny sometimes, jokes, but he doesn’t joke over the top. He doesn’t show off or anything, and his family background is really good as well. He’s the only child within the family, and he’s got a very good mum and dad who really support him and care about him. He’s like, similar to me; my mum and dad are like that as well. It’s like, some parents break up, and the kids act ruthlessly, they go out clubbing all night. He’s really responsible and he’s a really good mate. I can rely upon him, you know? He stands on my shoulders, I stand on his shoulders. For three years I’ve known him.. For nearly three years. I used to have a mate in college; I only had one friend in college, like really close as well. And that friend turned out to be a stupid.. I don’t want to swear, but you know.. I fell out with him because every time I was going out, I felt he was kind of using me? Every time we went out I paid for everything and he’s not giving me anything. And even if I asked, he’s like I’ll pay next and you pay for this for me. He shouldn’t do that, you know? He should say just one time you pay and I’ll pay the next time. He shouldn’t think of it as something that he’s really grateful that I’ve done. So, we fell out because of that. I don’t like.. So far, my friend [friend 1], he’s been great. |  |
| CH | Is there anybody who, like [friend 1] perhaps, that you’ve been friends with for a long time and you’ve lost contact with when you became unwell? |  |
| 11 | No, I’ve always been independent. I think with me, there was one guy, one boy in my school, who I lost contact with, but most of the friends I’ve had so far from my past, cos the school I used to go to.. Most of them are sleeping; when I’m revising they’re doing no work and stuff like that. So I used to - in class - I used to be serious and do my work, cos we used to be in different classes. They used to be in lower and I used to be higher up, so I used to do my work in the class and then outside of the class I used to hang about with them and it was much more fun. It’s much.. If you hang around all the time with the study type friends, then it’s boring. I’m not that kind of person. I like to keep my studying in my class, and with my friendships on the side. Friends are different to studying. Keep them separate. |  |
| CH | So when you left school, did you gradually lose touch with these people, when you went to university? |  |
| 11 | I became more independent. In college I was on my own most of the time. I didn’t have any friends that I can say, that were really close, that I could speak to or anything. I was really stressed. I was doing my A-Levels and it was two years of hard work on my own. I didn’t have any insecurity with myself or my studies and stuff; I didn’t have any problems and stuff. It was easy; work and just do it as it comes along. I didn’t have time for going out and stuff like that. I probably just played football and apart from that, nothing else. I put on a lot of weight as well, and then it just gradually built up. And when I came to university I had practically no friends who were really close or I could speak to properly. And then I met [friend 1], but [friend 1] wasn’t that close. After we left university we kept in touch through facebook and talking on the phone, and messaging each other, and eventually we met up and now we’re.. Not too close but close enough to become pretty close. But apart from that, at university I had no proper friends. It was like ‘hello’, ‘hello’ and that was it. |  |
| CH | So more like acquaintances? |  |
| 11 | Yeah. |  |
| CH | When you were unwell, and the time when you were starting to get better as well, do you think that would have been different if there had been more friends around? |  |
| 11 | No, I think it would have been… Even.. Because I didn’t have any friends when I went to university - even if I did have someone to turn to, they probably wouldn’t understand. They would not understand a single thing, and the only person who would understand is my family. My mum and dad. They’re the ones who.. I would look for help and actually supported me. Even if I had friends they probably just spread rumours or talk about it with other people, you know? It’s like that. I didn’t feel like I could have talked to any friends and stuff. I can’t now. I don’t want to tell anyone. Never. |  |
| CH | So you’re new friends that you’ve met at college, how did you.. How did these friendships start? You’re in class with them… |  |
| 11 | The only reason I went to college, was not just to do the [professional accreditation] course to become a [profession], it was to meet new people as well because I didn’t have anyone to hang about with and stuff. So I just went in with a smiley face and talking to everyone, and chatting, and my dad used to say be more aggressive when you’re with people. Not really aggressive but like, get into stuff, get out there and show what you are and who you are, and try and meet new people. When I was in the first week, this girl who I’m dating now, she said.. I told her I can cook, right? And she said ‘really’, I said yeah, but she don’t believe me, so I cooked for the whole class. She asked me to cook for the whole class so I did. I brought [traditional] food, and then people… It was a good introduction to the rest of the [professional accreditation] course, and they all had the food, and they were like ‘did you really make this’ and stuff and I was like ‘yeah, I can cook’. And then afterwards she started sitting next to me, and I got to know her more, and I met a couple of other people as well, and I got their numbers and we might go paint-balling a couple of weeks later. Its like.. Because I went into college with two aims; one was to make friends, and maybe get a girlfriend, and another was to get my career, to become a [profession]. It’s just been really good. |  |
| CH | It sounds really good. How was that for you? It’s scary on the first day, to make new friends, get your face known with these people and stuff – did that happen naturally for you or was it quite hard work? |  |
| 11 | When I aim for something, and I aim.. I make myself work hard, I actually do it. If I’m too optimistic I probably won’t do very well, but if I’m negative I know what I’m expecting. They might not have the same attitude as me, in getting to know you - some of them might be really quiet, and some might have their own problems and stuff. So I wasn’t sure what I was expecting. I was a bit like, cautious of people. So I went in there – it wasn’t too easy – but all I found was that when I was speaking to one person, speak out a bit more loud than normal, rather than speak normally so that other people can hear a little bit. So that way, it kind of got their attention, and turning and asking questions and stuff. That was a way of attracting people to talk. And also, in class the first question was introduce yourself, put your hand up and talk. I was like.. My name is [participant name], I’m this, I’m that, so.. Another thing was when she asked me to cook for the whole class, I was like.. ‘Alright, this is a good way’, because I’ll meet a lot more.. I’ll know a lot more what people are like, where they’re from, what their names are, know them even more. What I done was, rather than know everyone, I’ve forgotten some of the names now, rather than know all of them, take three or four and then remember, focus on them, and that’s what I do. |  |
| CH | It sounds like a pretty good strategy that worked. When you were in your first few weeks, was there anything that you looked for in your new friends? Any particular qualities that you would like your new friends to be like? |  |
| 11 | I don’t really look for qualities, but I do look for a couple of things, like not being rude or arrogant, friendly, smiling.. What else.. That’s about it really, just polite to one another and listening to someone, not going over the top and talking about themselves all the time, or about what they’ve been doing at the weekend, like drinking.. There’s one girl who does that. She just talks about constantly ‘I drank this’, ‘I done that’, you know? Stuff that’s her own personal problems. I don’t like it. It’s just too many. I can’t really explain it. It’s like they know what kind of person I am and then they show what kind of person they are. Not just about themselves. There are some people who talk too much, like blah blah blah, they just talk about themselves all the time and what they’ve achieved. I don’t like that. I don’t like that kind of person. |  |
| CH | And do you think that now, since you’ve been unwell, you look for different things in friends than before, or do you think it’s about the same? |  |
| 11 | When I was unwell I wasn’t looking at friends. I was lost. I was completely lost. I was insecure. I was frightened. I was even paranoid with my parents that they hated me and stuff like that. When I was unwell I couldn’t.. There was no one. I didn’t trust anyone. And then when I got better, gradually, I wanted to make new friends, but I couldn’t find new friends because I wasn’t at college or studying or I wasn’t out and about working. I wasn’t doing anything, practically. I was practically in all day. So rather than look for new friends I thought.. At that time I was thinking.. I went and talked to my cousins, hanging about with them more. And then one of them pushed me away, he didn’t have any time because of working and stuff like that, then I was looking at my parents. And that’s what pulled me out, because of my parents. I don’t know who else would go through that and help me. And then after when I was well and I had.. My head was clear and I didn’t have a lot of problems and I was having the support every week with my coordinator and stuff like that and doing the social activities at the EIS, and then there was just getting my head straight, making one or two plans. Getting, going to do a course, getting my own place, you know, stuff like that. I just wanted to do something to make new friends. I went to [coastal town] the other time, a couple of weeks back, it was pretty good. I met a couple of people there and it was really fun as well. |  |
| CH | Ok. I’m going to ask you about your friends from here in a minute, but I just wanted to also check with you: do you think that you’re now back to how you were before in terms of socialising, being around people? |  |
| 11 | I feel more better, much better than before, because I’ve got less stress in the sense that.. Rather than just focus on uni, college, uni, coursework, I feel.. My head’s in the right direction. I’ve got a balance now with my social life. Because the thing is, I know for a fact because of depression, studying and stuff like that just built up. I know for a fact that my health is worth more than studying and grades and whatever now so I’m just thinking there are people out there who don’t go to uni, don’t go to college, and they end up working in the bank or whatever, doing good things with their life and they’re really happy. They’ve got families now and kids and stuff like that. I’m just thinking, I feel its time where I’ve picked my career. I just follow that path, go to accountancy with not a lot of stress and on the side have a relationship at the same time, or go out with friends and stuff like that, general stuff. I’m young so I’m thinking – before I was like an old man, thinking work, work, work, money, money, money – now it’s like I want to go abroad more. Last time - a couple of days ago when I went to Barcelona with my friend - before that I hadn’t been on holiday for nearly.. 2008.. So that’s two years now, and that was with parents. That was when I was unwell, just to get me away from here, so it wasn’t a holiday to be honest. So apart from that I haven’t been on holiday for six, seven years. So I am thinking every single year I’m going to go on two or three holidays. I don’t care where the money comes from. |  |
| CH | So it’s shifted your priorities a little bit? |  |
| 11 | Yes. Its like, go out socialising more, and when you’re at college just do your work, but don’t go over the top with the study like before. I was addicted. |  |
| CH | It sounds good. Thinking about your friends from the EIS groups – you went to [coastal town] – have you done any of the other activities? |  |
| 11 | Yeah – badminton, been out to the cinema, we went to.. I missed the Christmas dinner.. But I’ve gone to a couple of other places. We’ve gone to the zoo and stuff like that. It’s been really good. Before, when I first started like going along to the activities, I wasn’t making a lot of friends. The very last trip, the [coastal town] trip, that sort of woken me up a bit, cos it was seaside, away from fumes, all the stress and all the traffic. When I went to [coastal town] I was like.. Peace. There was me and some other people, just joking around. I saw a guy, a friend, I can’t remember his name but I remember him, and he was like ‘check out these girls’ and asking questions and talking and stuff. I met another girl in the group, I got her number and stuff but I text her once but didn’t phone her. I thought, you know what, I don’t know if she’s alright to do this. Anyway. |  |
| CH | When you were going to the groups, was it the same sort of idea that you had for college? I’m going to go here and meet friends, or was it something different? |  |
| 11 | It was different. It was to see different things, and the thing is in college, cos you’re in college twice a week I see the same people every day. But because I go to social groups like one off, like something might happen once a month usually. The [coastal town] trip happened once a year. Its not like, very frequent. There aren’t like.. Some of the people in the groups, they don’t talk. I don’t know who I talk to but there are some that are alright. But most of them they just want to keep themselves to themselves. I know how it is. When I was like it I was like that as well. I didn’t want to. I don’t like to go hassle them as well because I know they’ve experienced similar experiences. I know their situation so it’s completely different. College is like, you can speak to everyone and don’t tell them stuff like that. In the EIS groups it’s not like that. It’s just like you can go out and enjoy it and stuff, but don’t get too close. That’s how I felt. |  |
| CH | In the EIS groups, is there anything that’s good about the fact that you’ve all had similar experiences? |  |
| 11 | Yeah, it is good, but I don’t like talking about it. I don’t think others in the group; they don’t like talking about it as well. It’s difficult. The thing about it is that you enjoy yourself. You meet new people, and you can talk normally, but you can’t get too personal and tell someone everything, or most of the stuff; it’s like 25%. But at college you can tell up to 50-75%. |  |
| CH | Why is there that difference? |  |
| 11 | I think the difference is because, number 1 it’s difficult to explain, and number 2, people get.. I get emotional when I have to explain. I feel its difficult because when you’re talking personally about the experiences you’ve had, its really difficult to explain, even in the groups, even if they’ve gone through it, they might not have the extremely same experiences. In college you don’t have to say anything to anyone about it. You just talk like normal people. But when you talk about your own experiences and stuff like that it’s a personal thing. Its really difficult in the social groups. |  |
| CH | So in the social groups, because people have had similar experiences they are expecting you to talk about that? |  |
| 11 | Yeah. Its like even if you did explain it, even if they had a similar experience, its just.. It’s because when you explain it I get really emotional; I can’t explain it. It’s difficult to talk to people when you’re the person. |  |
| CH | I’m thinking about when you were unwell and the people around you. Is there anything that would have helped in terms of friends, having someone to talk to? |  |
| 11 | Difficult question. I think what would have helped was someone who could make you go out more, to get well. Just going to the cinema, just get things out of your mind rather than.. The thing is that the care coordinator already.. [care coordinator] already talks to me and that helps, and medication and all of this. That’s already there. It’s just.. You need someone to help you go out more, like go to the cinema. A friend who you can go out with, not a health professional. Like a normal friend - an ordinary person. You don’t have to tell them your personal experiences but someone who’s just there and you can just get support to go out and do stuff, like play football, like someone who’s a really close friend. |  |
| CH | Who can perhaps take your mind off things? |  |
| 11 | Yeah, take your mind off things. That’s what it is. |  |
| CH | That’s a useful point. So you’ve started college, you’ve got a new group of friends, you’ve still got [friend 1]l from before. Is there anything you think is missing in terms of friendships? |  |
| 11 | What do you mean, like..? |  |
| CH | In an ideal world, and if things were exactly as you want them to be, would it be different to how it is now? |  |
| 11 | Not having too many friends is not good. But having the wrong friend is even worse. Like, I know a guy who’s.. You know, he’s alright but he tries to act too clever. He’s thinking I’m not seeing what he’s seeing. Like he’s.. I’ll give you an example. He’s going out spending lots of money on cars and stuff like that, probably gambling as well at the same time, and he’s taking other people’s money to do that. He’s a student, you see? He’s not working or anything. And, you know, those types of friends you should stay away from. Or if you’re friends with them, if you’ve got any money with them or stuff just get out as quick as you can. And there’s other friends who try and take your money, like try and make you spend, spend, spend when you’re with them and they never do anything about it. There are other friends who lie to you in your face, and later on you find out. I’ve experienced quite a few friends like that actually. I think in an ideal world what you would want from your friend is the same characteristics as you. Similar characteristics as you. I don’t mean like a relationship or anything. Even like a mate has similar characteristics, similar background to you, like working class or someone you can get along with, someone who doesn’t joke too much, someone who doesn’t go over the top, someone who’s not in to themselves, like a bad boy or too nice.. Someone who’s genuine and not someone who’s taking advantage of you. There are a lot of people out there who do that. I think that’s the most ideal friendship that you can have with someone. Someone who really cares for someone. Doesn’t play with your emotions. |  |
| CH | These “bad” friends, these unhelpful people that you describe, have there been many of them in the last year or two years? |  |
| 11 | Basically because I pick my friends, I’m really careful about making friends with someone. I only make one or two. I have had the experience of that but its been my mistake in the sense that I shouldn’t have helped them out or.. It’s been my mistake. I should have thought of that, or got advice from someone else before I actually helped them out. I should have found out more about them; what kind of person they were, rather than just taking their word and stuff like that, so there have been one or two. |  |
| CH | Are those people before you were unwell or after you were unwell? |  |
| 11 | Before. |  |
| CH | Do you think you’re more cautious since you were unwell? |  |
| 11 | I think.. After I’ve been unwell - because I’ve had two targets: one to get accountancy job, and another to make new friends - because I’m focussing on making new friends, I feel I’m more cautious deep down than before because before I was more into my studying and I had my mind on work. I was like, more stressed then and less stressed with friends. Now it’s like 50-50, so I actually see what’s going on. |  |
| CH | So rather than being more cautious, you can pay more attention and put more time into it? |  |
| 11 | Yes |  |
| CH | Do you feel that you have enough new friends now or are you still looking to add to that? |  |
| 11 | I don’t mind. I probably want to add more, but if I had too many, maybe more than 7 friends.. 7-8 in a week is too much. |  |
| CH | Is there anything I haven’t asked you to do with friends that you’d like to say? |  |
| 11 | No. |  |
| CH | I suppose I’m curious about starting college and your decision to use this opportunity to make friends; was there anybody who suggested that to you or talked to you about that? |  |
| 11 | No, I realised that I needed to. I don’t want to be on my own for the rest of my life. I didn’t take the opportunity at school or in college. I actually moved from my school… My college is really far in [area of the city], my school was in [area of the city] so I actually moved out of the [area of the city] crowd just to get out of the area because of the atmosphere and the school was absolutely.. It was alright but I used to get bullied in that school and stuff like that, so I just felt that I needed to move out of the area and go as far as I could to a good college and meet new friends, but I kind of isolated myself. I already had a couple of friends from school but I isolated myself – why didn’t I go to the same college as them and stuff like that? A nearby college. I made the right choice for me because it freed up my mind and focussed on my study even more. |  |
| CH | Thank you for taking part. |  |
