# Supplementary material for: Experiences of friendships of young people with first-episode psychosis: A qualitative study
Source: PLoS One. 2021 Jul 30;16(7):e0255469. doi: 10.1371/journal.pone.0255469 (PMC8323937; doi:10.1371/journal.pone.0255469)
Supplement: S1 File — (ZIP) [file pone.0255469.s001.zip › Transcripts of interviews/Participant 12.doc]

| CH | Okay. So, what I’m interested in hearing about is people in your life who you see socially or who you consider to be friends. Have you got anyone in your life like that at the moment? |  |
| --- | --- | --- |
| 12 | I do. |  |
| CH | Okay. Can you tell me about them? |  |
| 12 | Someone I see as the most memorable, the most close with this, is someone called [Friend 1]. I’ve known him for about four years I think. |  |
| CH | Okay. |  |
| 12 | And he’s a very close friend of mine. |  |
| CH | How did you and [Friend 1] meet? |  |
| 12 | We met through a friend but when.. I used to do GCSEs with someone who became a friend and then we moved onto ‘A’ Levels and we separated. I mean, in terms of ‘A’ Levels, we went to different colleges. But then we sort of stayed friends and at the college that he went to, my friend from GCSEs, he met [Friend 1] and we got introduced. |  |
| CH | Okay. And what sort of things do you and [Friend 1] do together? |  |
| 12 | We used to actually go to parties and stuff together but he stopped that. Without him having any problems, he just felt like he didn’t – he was taking drugs a lot and I was as well and he just decided that wasn’t for him anymore and he just stopped without any experiencing any negative phase. |  |
| CH | Okay. |  |
| 12 | Not any strong ones, maybe he did have some kind of mild negative, very slight depression or something like that. And then he stopped but I had to stop because I had other problems. But now – when I had – you’re probably aware – psychosis, I still did them. He was one of the very few ones who.. I used go gardening with him every week and that was quite helpful, I think. And now we just meet up and go to a bar, something like that. |  |
| CH | Okay, so when you were feeling quite unwell, perhaps worse than you do now, [Friend 1] was around to do stuff with you? |  |
| 12 | Yes. I mean, [Friend 1] was quite helpful and I got a lot of support. |  |
| CH | What was it that he did that helped? |  |
| 12 | We just generally preferred to.. We did something together. Ourselves, alone, we spoke and sometimes brought it round to my problems and we were discussing it a bit. That helps as well. He would try to help me in that way. |  |
| CH | Okay. Are there any friends who you’ve lost touch with or are not in contact with anymore as a result of being unwell? |  |
| 12 | There are, yes. It’s mainly those who are – even though [Friend 1] – actually, [Friend 1] was the one who took us first to this psychedelic trance club sort of thing where we started to – before we were taking drugs as well – and that’s when we moved onto psychedelics and stuff.  Then for some reason I didn’t have this kind of fear of anything like that. There were other people who I used to see which was after [Friend 1] had stopped taking drugs but I was still taking them. Those were the ones who I took most drugs with and when, for example, when I had a bad trip – which happened twice to me – there were the people who I saw first and it was the first time I’d taken acid in a negative kind of light. So after that…  When that happened the first time, I was fine. I had some weird thoughts at first but then kind of got over it, but when it happened the second time and it was full on psychosis, like I fear them a bit and still I sometimes think about how I wouldn’t necessarily want to see them. |  |
| CH | Okay, so, because they became associated with that time they became frightening; is that too strong a word? |  |
| 12 | It did at the time, yes. And also, especially one of the girls, I used to be in this sort of.. I guess kind of psychosis but she would... For example, when I lay in bed to go to sleep, she would - there were mental images in my head where she would be playing games with me and stuff and they were with me for quite a while, so I just didn’t want to be associated with her. |  |
| CH | Okay, okay. Thinking about the difference between [Friend 1] and those people and why your friendship with [Friend 1] has survived, what is it about [Friend 1]? What qualities does he have that makes him such a good friend do you think? |  |
| 12 | I guess once you get close to him and get to know him, he’s very warm-hearted and he’s just sort of a very warm person and nice to be around I guess. He tends to be quite positive and our conversations are quite free and natural. When we’re together, we feel that we can talk about all sorts of stuff together and he’s quite open in the relationship we have. I guess it’s kind of his character and my character that are kind of – or maybe I’m not just like that with other people and he’s not like that with other people, but we kind of connected somehow. |  |
| CH | Okay. Were there any other friends who were around beforehand who are still your friend? |  |
| 12 | No. It’s not – there are – I wouldn’t call them friends so much now because I don’t see them that much, but they will appear every time – when I was at my most ill, I kind of stopped seeing most people but then as I’m feeling better, I keep some kind of contact with them, with those people. But we don’t see each other that much. Not necessarily because I don’t want to, I just think it’s.. Obviously I.. A few new people have appeared and it’s kind of steps, the concept with a group of friends kind of thing, so, yes.  But there is one other guy and my ex-girlfriend as well who I knew before it happened to me. |  |
| CH | Okay. And were they around like [Friend 1] was when you were finding things really difficult and starting to get back on your feet? |  |
| 12 | Well, my ex-girlfriend was kind of most involved because we met about two months before anything happened. |  |
| CH | Right. |  |
| 12 | Or the second time it happened. And then after a month of our meetings, so the month before anything happened, we got together and she was with me through the whole thing, so she was very important. |  |
| CH | And was that helpful or unhelpful or what? |  |
| 12 | Yes, it was helpful mostly, yes. Yes. I mean, mostly I say because I guess she would talk to me but at the same she would – because she would get quite depressed as well and every so often we would argue as well, which I guess was not helpful in some ways, but… Yes, in general I think… |  |
| CH | Okay. And how about that other friend? Because you said your ex-girlfriend and somebody else. Who was that? |  |
| 12 | Yes. Who introduced me to my ex-girlfriend. |  |
| CH | Oh, okay. |  |
| 12 | This guy was called [Friend 2] and my ex-girlfriend was called [Friend 3]. So [Friend 2], he’s less of a serious guy, I guess. He’s not very serious. But he was still not far away from me and we still kept in contact and we spoke sometimes. He would sometimes - the best place for me to be was my house, so they would sometimes come to my house and stayed. I think I was seeing him less than [Friend 1]. |  |
| CH | Okay. And are the sorts of things you do now with friends in general, that you do for fun, is that different? |  |
| 12 | Yes. I mean, before I would be taking drugs and now I don’t do that. |  |
| CH | Okay. So does that mean going to different places as well? |  |
| 12 | Not necessarily, no. |  |
| CH | Okay. |  |
| 12 | The actual psychedelic trance parties, as they’re called, which are a big kind of – building of raves, sort of thing, they were sometimes in clubs but now they prefer to put them on in warehouses. I went maybe two or three times over the past two years, so I don’t do that anymore. Maybe people…  I mean, I’ve kind of managed to bond or talk – I think I have to be less self-conscious – and I’m able to talk to different people once I find something in common with different people. Usually I go to this place in [local area], which the people who go there usually tend to go to these psychedelic trance parties so that part didn’t change that much. But obviously they go to other places as well. But most end up going to these.  Because it’s like a kind of home for me. It’s nicely made and everyone kind of knows each other and they’ve shown good… |  |
| CH | Okay, so it has to be sort of somewhere familiar, that kind of thing. |  |
| 12 | Yes. |  |
| CH | Okay. In terms of talking about your symptoms and the time when you were unwell and everything, who would you do that with? Are you quite open about that? Would that be with most people? |  |
| 12  CH | I’m quite open, yes. |  |
|  | Okay. Who’s the most – who would be the first person you’d talk to if you wanted to? |  |
| 12 | It would be my ex-girlfriend and my mum. My ex-girlfriend not so much now because she’s got a new boyfriend, which I don’t mind at all. But my mum, if only very slightly something that feels a little bit strange, I’d kind of – because it’s nothing that bothers me that much, it’s not making me feel really, really depressed all the time as it used to, but things can happen sometimes but it’s not having a huge effect on me. She thinks that – it’s true that I feel much better – and she’s very happy about that, so I don’t want to put – because of these little things that can happen sometimes – I don’t want to put extra burden on her. |  |
| CH | Okay. |  |
| 12 | With some other friends maybe I can… [Friend 1], for example, or my twin brother. |  |
| CH | Were you worried about that burden on [Friend 1] or do you think he’s better able to manage it? |  |
| 12 | No, I think he’s better able to… |  |
| CH | Okay. And thinking then about new friends; have there been  any new friends since you were unwell? |  |
| 12 | Yes, there is a very pleasant girl from my university who I’m friends with. Not so long, but she seems like a really nice person and I’m available at the moment, so if there’s anything there… I quite like talking to her in general and also if there’s anything about this I talk about as well. |  |
| CH | Okay. Is there anybody else? |  |
| 12 | It depends. Like I said, I’m quite open so I would tell that to most people and try and get maybe some answers from most people for me so that – not everyone, but most people. |  |
| CH | Okay. And if you’re looking for friends now compared to when you were unwell, do you think you try to be friends with different sorts of people now or is it the same? Are different qualities important to you now? |  |
| 12 | I don’t think about it that much. In general, I think usually I don’t have a very strong opinions about this or, “I like that.” It’s just I feel rather thinking about – maybe in the moment I might think about it but after that it’s not kind of the words that maybe describe it, it’s just a very nice person and warm.  Rather than judging me – not judging me. |  |
| CH | Okay. And if you wanted to make new friends now, if that was something you wanted to do, where would you do that or how would you go about that? |  |
| 12 | I wouldn’t think too much about that. I would just – maybe not thinking about new friends at all, or even if I was, I wouldn’t necessarily go about where I would maybe take an approach… just letting it come as it comes kind of naturally. Not going to a place to make friends there or there or there. Just make as much friends, that’s all. |  |
| CH | Okay. Do you think being unwell has changed the way friendships are for you? I mean, for example with [Friend 1], do you think that your friendship has experienced change to how it was before or is it the same? |  |
| 12 | With [Friend 1] it’s different. Also the psychosis brought us closer together and then I feel it’s more positive, you know. We’re just a lot more closer and able to talk about loads of stuff. |  |
| CH | So how about [Friend 2] and [Friend 3]? Do you think those friendships have changed? I suppose [Friend 3] has in a way because now she’s your ex-girlfriend and she was your girlfriend. |  |
| 12 | Yes. I mean, after we broke up, we were sort of friends. I think it was harder for her than it was for me. Yes, now it’s kind of… I used to call her a lot to ask – not because I was chancing on her, but because I was so dependent on her before. Maybe I was attached in some way. Anyway, she’s got a boyfriend. She doesn’t want that so much. She tried to be a little bit distant, but we’re still friends.  And [Friend 2], after I got better I continued to see him, but now somehow, I guess he’s busy and doesn’t come out as much and he lives quite far out so, he travels less. |  |
| CH | Okay. We talked a little bit about how friendships might have been affecting you when you were starting to get better. Do you think that there was anything that you’d have wanted friends to do more for you when you were trying to get better? |  |
| 12 | Maybe – I don’t know. Maybe sort of more talking about this because – actually, I can’t remember exactly but it was…  Maybe, like my mum would be very kind of strict. Strict in a sense that she would for a long time explain why I was having these experiences and say, “You have taken these drugs and that’s why you’re feeling like this. Don’t be silly now. You’ve heard of so many other people telling you about how it is and that’s why you’re experiencing this stuff,” so reassuring, she was always very supportive in that sense. Always saying I’m going to get better, which I didn’t get as much from my friends. |  |
| CH | What sort of messages did your friends give you? |  |
| 12 | I don’t know. Again, it wasn’t – I guess the same goes. I wouldn’t find they’d explain things – I mean not so much. Because it used to happen with my mum and my ex-girlfriend as well, “you’re going to get better”. |  |
| CH | Okay. But do you think they were reluctant to talk about your symptoms with you? Do you think they were a little bit unsure? |  |
| 12 | Yes, sometimes. Not all. |  |
| CH | Okay. If you were going to change something about the friendships that you’ve got in your life at the moment, whether that’s to get some more or to have less or different sorts of friends, what would it be for you? |  |
| 12 | I don’t know. I don’t know. |  |
| CH | Okay. Do you feel there’s anything at the moment that’s missing from your friendships? |  |
| 12 | Yes, I guess – I mean, I don’t think so... |  |
| CH | Okay, so it feels quite kind of content with how you.. |  |
| 12 | Yes, it’s fine, yes. |  |
| CH | Okay. Good. Does anyone here ever talk to you about friendships and social stuff? Anyone at the intervention service? |  |
| 12 | Yes. Mostly [care co-ordinator’s name]. Mostly about the relationship with my ex-girlfriend because I’d talked about her before. Not so much, but a little maybe. |  |
| CH | Okay. And did you ever come to any of the social things that they ran here? Any of the groups or activities or that? Were you offered them? |  |
| 12 | Like badminton and stuff? |  |
| CH | Yes. |  |
| 12 | Yes. At the time I wasn’t heavy about getting involved with all that with other people. I think that’s fine. Now I’m very open with other people and usually welcome people. But I was told by some girl I spoke to that I was too open again. I should be more cautious about people. |  |
| CH | Right. Do you think you’ve always been like that? Sort of quite open, or is that something new? |  |
| 12 | It’s something new for me. |  |
| CH | Okay. And what did you think when this girl said that? Did that ring true for you or was it a surprise? |  |
| 12 | I would maybe like to see – I kind of accepted how I was. I thought if I was with [Friend 2], it might be too much. I don’t know. But I always like to think that she told me that in a sense that – most people would talk about it in that you can’t always trust people or something like that. It’s kind of much more the thoughts people might have about other people that you can’t always trust.  But whereas more psychotic thoughts connected to my fears might be hinting something that actually, be careful, because… You know, I haven’t actually about it, I think, so much. I was just trying to be open, so she could also be saying, “Okay, you’re very open, but be careful,” because people could be going against me and stuff. |  |
| CH | Okay. I’m just wondering what it is about being unwell that’s made you be more open and sort of welcoming to people? Do you know? It’s hard to say, I know. |  |
| 12 | Well, I don’t know, because again, I don’t think about this because like… I mean, maybe it’s just this fighting element talk, usually we have this thought that we have business to fight things in our life. When we’ve got problems, maybe we’re working to try harder, stronger, you know, subconsciously to overcome these problems. And where you’re open, maybe – like when you feel everyone is nice to you and stuff like that, it’s another way of looking at it which I haven’t, as I said, consciously – or maybe sometimes I have and I’ve realised how much effect it’s had.  I’ve told myself over the times, “No don’t be silly, people are fine and everything,” which at the time didn’t seem to make a difference in my moods. And not much anyway. I was still thinking the things that I thought about but maybe it had some kind of difference.. |  |
| CH | Okay. So to compensate for the power of the frightening thoughts and kind of compensating by forcing yourself, as you say, not necessarily consciously but by being very welcoming and open with people? |  |
| 12 | Yes. And also.. That was at the time – but I don’t – things that happen now I think have come naturally kind of thing. And also maybe because I’ve aged and stuff. I’ve become confident again, talking to people. |  |
| CH | Did you notice after you were unwell that you had a drop in your confidence? Some people tell me this, but not everybody. |  |
| 12 | A drop in my confidence… |  |
| CH | In terms of being around other people socially? |  |
| 12 | I don’t know whether it’s confidence or not, but it was just more of a kind of fear of what they want. It’s not necessarily how I look or anything. I don’t know. I don’t really do much thinking about that stuff. |  |
| CH | Okay. Is there anybody that has reacted badly when you’ve talked about your symptoms and talked openly about your illness? Is there anyone who’s sort of reacted negatively to that? |  |
| 12 | Not really, no.  Everyone’s been really supportive |  |
| CH | Okay. Let me just check if there’s anything I’ve missed. Do you think there’s anything else services could be doing to support you in friendships? Is there any need that you’ve got that’s not met? |  |
| 12 | I can’t think of anything. |  |
| CH | Let’s think about perhaps the friendship with [Friend 1], the sort of role that you play in that friendship. |  |
| 12 | I guess – I’m always there to talk to him about whatever he may like to talk about. And I would like to be able to give advice whenever I can, if I’m able to give advice. I do sometimes, maybe. And I would – if he needs any help at all, I would be willing to help him as much as he wants, as much as I can. Or just in general, going out with him and having a good night out or – yes... |  |
| CH | Is that the same for [Friend 2] and [Friend 3] do you think? |  |
| 12 | I think, yes. I mean, again, help is something that I’d be willing to give both of them very much. Talking with [Friend 2]’s perhaps less easy now, but… Yes. |  |
| CH | Okay. In terms of the roles that they play as friends to you, I mean you’ve said that you are kind there as somebody who listens, talks, supports, that sort of thing, do you have the same need from them or do they give you something different? |  |
| 12 | They do give me that, yes. |  |
| CH | And do you think your role or any of those roles changed since you were unwell or have they always been the same as that? |  |
| 12 | In the period that I was unwell or now? Is that – |  |
| CH | I’m just wondering; some people find that after they’ve been unwell, things that they need from their friends are a little bit different or the things that they give to their friends are a little bit different, and again, not everybody. I don’t know if that’s the case for you? |  |
| 12 | I’m not sure. |  |
| CH | Okay. Is there anything else that you can think of? Any comments about friendships in relation to your illness before or after? |  |
| 12 | No. |  |
| CH | No? Okay. |  |
