# Supplementary material for: Experiences of friendships of young people with first-episode psychosis: A qualitative study
Source: PLoS One. 2021 Jul 30;16(7):e0255469. doi: 10.1371/journal.pone.0255469 (PMC8323937; doi:10.1371/journal.pone.0255469)
Supplement: S1 File — (ZIP) [file pone.0255469.s001.zip › Transcripts of interviews/Participant 13.doc]

| CH | Okay so do you feel that you've got any friends in your life at the moment? |  |
| --- | --- | --- |
| 13 | Yes. Just on Facebook. I'm in touch with my friends and cousins, I write to them and they write to me but I don’t see them day to day, I only see one of my friends from college once every two or three weeks because she works at her dad's shop so she's not always free. So apart from her I'll see - I was going out with a boy but I ended it with him, I was just seeing him and that's all. |  |
| CH | Okay. |  |
| 13 | I'm not seeing many people at the moment. |  |
| CH | Why's that? |  |
| 13 | Because I write to them on Facebook, they write to me but they never say, "Let's meet up" some - most of them have got married, some have kids, some don't, but they're working and they set up a new life after they got married. And they say - one says, "I'm busy, my life changed after I got married, I'm busy all the time" and then she didn’t give me her phone number on Facebook when I asked her for it, because she changed it after she came back from holiday and then she didn’t give it to me. And I've got a step-mum, and my step-mum who knows the girls' mum, we're distant relatives of the girl, and my step-mum might have influenced the mother saying, "Maybe your daughter shouldn’t see [Participant Name]" or I don’t know what possibly could have gone wrong, but something went wrong, my instincts tell me my step-mum might have something to do with it. |  |
| CH | Okay, so was it… |  |
| 13 | My step-mum and me didn’t get on so she might not want my friends to be around me. |  |
| CH | I see, okay, okay. |  |
| 13 | She might want to leave me alone. |  |
| CH | And did you lose touch with this friend - has that happened since you were ill or was that before you were ill? |  |
| 13 | After I was ill, about a year ago, before she got married, six months before that I stopped going to the house because she would never call me, she would say, "I'm busy, I'm getting prepared for my wedding" and she wouldn’t call me, she would say, "I'm busy, I'm not running away from you but I'm just busy." And after that she said she got married and then this time she says she's busy, she's got guests, visitors coming and her life has changed and she hasn’t got much time to meet me. She says I just - I speak to her - "I write to my friends on Facebook, I don’t see my other friends either" that’s what she says. |  |
| CH | Okay, okay. So you feel like… |  |
| 13 | Maybe she's lying. |  |
| CH | Okay I was going to ask you do you think she's being truthful or not, it's hard to know isn’t it? |  |
| 13 | Yes. |  |
| CH | Okay and how about your… |  |
| 13 | And her husband also could have maybe not wanted her to be - maybe her husband thought I was a bad influence and didn’t want me to be friends with her. Maybe her husband said, "Don't meet with her" but he knew me before and we would speak as friends, he's a good person but he may just not want me to be friends with her. |  |
| CH | Okay, what would make you think you're a bad influence do you think? |  |
| 13 | Well I went to [Country of origin] and I got engaged to someone without telling my dad, I met the boy on MSN, I spoke to him for three and a half years and then I got engaged, I went there and got engaged with him and then my aunts got involved and said, "You are using [Participant Name] to go to [city], if you wanted to - if you were serious about this marriage they said that to his mum and dad, "You would go and see [Participant Name]'s - you would come and see [Participant Name]'s uncles in [City of Origin]. If you were serious about this marriage." Because it's a cultural thing like you have to ask permission from the parents or the uncles.  And so then the boy didn’t want me, and then it was that girl, my friend, who had bought my ticket to go to [City of Origin] but she knew I went to another part of [Country of origin] to meet him but I never told my - I never told my dad and my step-mum. I said she didn’t know that I was going to go there, she only bought me the ticket to [City of Origin] and from there I went myself. So then my dad and my cousin said, "That girl bought the ticket, whoever bought the ticket is the one that is wrong", and I think my dad was upset with her mum and so then this could have affected my friendship with my friend because she got the blame for buying me the ticket. |  |
| CH | I see, okay, and that's why her husband might not be happy. |  |
| 13 | Possibly and her mum as well. |  |
| CH | Right, okay. Tell me about your friend from college who you see every few weeks. |  |
| 13 | She's a very good person, we get on, we enjoy our time together. We go and eat together, sometimes go to the cinema but not so often, we just meet up and talk. I tell her about my relationship with my boyfriend, when I split up with him and when I got back together with him, I told her about it. And then she listens and she tries to give a little bit of advice, she says, "I'm not really going to give you advice because you take it the wrong way, you don't want to listen, so from now on I'm not going to give you advice on what's going on in your relationship" but she just listens mainly. |  |
| CH | Right okay. What is it about your friend that you like? What are her good qualities? |  |
| 13 | She listens, she's a good listener, she cares about me, she wants the best for me, she thinks about me, she wants the best. |  |
| CH | And how long have you known her? |  |
| 13 | Since college, 2002. |  |
| CH | Okay a long time. |  |
| 13 | Eight years. |  |
| CH | Was she around when you were ill, when you were unwell? |  |
| 13 | Not right next to me but I was still seeing her, but there were times that I think I wasn’t seeing her. I was living in the hostel and I wasn’t seeing her that often, then I started seeing her more often. She knows my illness as depression, I didn’t tell her it was psychosis, she doesn’t know that I hear voice - I hear people calling me names in the street when I walk past them. I don’t hear voices when I'm on my own at home or anywhere, I only hear voices in the street when I go past someone, people on the street, just strangers, and then I think they're saying - they're putting down my appearance.  I'm insecure about my appearance, in the hostel they were calling me monkey, chimpanzee and then since then I haven’t got much confidence in myself. |  |
| CH | Right okay, is there anybody; is there any friends who you've told about what happened to you? |  |
| 13 | Sorry? |  |
| CH | Are there any friends that you've told about psychosis? |  |
| 13 | I told my boyfriend about it and he was supportive. |  |
| CH | Right. |  |
| 13 | But who else have I told? I told the boy in [Country of origin] when I got engaged, I told his mum and dad and they told - I told his dad and his dad told him, but I told them I had schizophrenia so then they didn’t want to know me anymore.  He said I don’t want to - he didn’t say, "I don’t want to know you" he said, "Don't think I never loved you, I loved you but it's not working, things are not working, your aunts got involved" and then he got upset that my aunts got involved and said that he's only using me to come over to [city], he lives in [Country of origin], that's my ex-boyfriend and then I had - basically I got through a lot of hard time, a lot of heartbreak. |  |
| CH | Yes it sounds like it. |  |
| 13 | I said it myself, I wanted the truth to come out so that in the future if we get married, if he finds out he won't leave me or he won't get angry at me, so I told him from the start and then it didn’t lead to marriage, we ended up not getting married. I spoke to him for three and half years on MSN and on the phone and then 10 days I saw him, in 10 days I got engaged. The first time after three and a half years I went and saw him, I never saw him before that, and then that was after he came back from the army in [Country of origin].  And after that I went for seven days where we were going to go, basically in 17 days seeing him face to face I was ready to marry him and now when I look back I think it was a stupid idea, if we got married it would have ended up in divorce, but it was because I was so much in love with him and he said he had a lot of feelings for me as well. |  |
| CH | Okay, so do you think that the relationship split up because you told him you were not well? |  |
| 13 | Yes. |  |
| CH | Okay. |  |
| 13 | No that wasn’t the whole reason, that was part of the reason and then he said, "Your aunts got involved and said that I'm using you to go to [city]" that was another reason, and another reason was after I came back from [Country of origin] the first time and then I was all jealous and paranoid of him and saying I saw in my dream that he was seeing another girl, he was seeing girls and I want it to end. He said, "I can't take your jealousy and your paranoia anymore, you're too much." And then after that I went out with someone else here in [city] and then after I went to [Country of origin] - before I went to [Country of origin] the second time he made me admit if I went out with him or not and if I slept with him or not.  And then I told him the truth and then he said, when I went to [Country of origin] he said to me, "Your aunt is saying that I'm using to go to [city], it's just an excuse" what he means is that you were unfaithful to me after we just split up for just one month you went out with someone else. And then after I went out with someone else he still called me back to [Country of origin] for the marriage. He said, "I forgive you." And I don’t know whether he had really forgiven me or he just thought, "Let me get my own back on her, let me go and get married with her and then leave her when I get my pass, my leave to remain in [city]."  I don’t know what he thought, he just called me back and I went back because I cared about him so much. Obviously I didn’t show him that I cared about him too well by going out with someone else. |  |
| CH | Okay, and when you were… |  |
| 13 | I've basically made mistakes in my life. |  |
| CH | Yes everyone makes mistakes don't they, and things that they regret perhaps. When you were feeing really unwell it was at its worst… |  |
| 13 | Yes they were still calling me monkey, chimpanzee, I had arguments with about 20 or 30 girls living in the hostel, it was an all girls hostel, in the mixed hostel I hadn’t gone through psychosis. I was only diagnosed with psychosis last year, 2009 in January or February, that was - but I was meanwhile before that going through psychosis and not aware of it. Only when my manager at the hostel referred me to the GP and then the GP referred me to [North [city]] mental health team and that's when they diagnosed me with psychosis, that was in my second hostel, which was an all girls hostel.  Then I was in the mixed hostel, I moved from one hostel to another to get a flat and then basically I didn’t have psychosis in my first hostel, so I didn’t hear it when it was a mixed hostel but when it was an all girls I felt that they would gang up on me. They were all calling me names, then I was calling them names and then the hostel gave a report to the council that [Participant Name] was the one who started calling them names, they never called her names, so I don’t know. |  |
| CH | Okay so at that very worst time, when the psychosis was really strong, were there any friends about for you then? Was there anybody who…? |  |
| 13 | I wasn’t seeing my friend from college, I was seeing my friend from - the one who got married, and she didn’t give me her number, I was seeing her but I never told her my illness was psychosis, I said it was depression. And I didn’t used to tell her that - I think I did tell her a little bit that they were calling me names and I was calling them names but I don’t remember very well if I told her or not. I think I did tell her, yes I said, "I've got problems with girls in the hostel, I'm arguing with them too much", that's what I said. |  |
| CH | And was there anything that she did that helped? |  |
| 13 | She was just there for me, she was a good friend while I was in her company. She was good fun, good laughter, she's really good at making jokes and imitating the way people speak, she takes the fun out of them; she mocks them, not in an evil way, in a nice way, just by…  Just teasing but some of them like her aunts she makes fun of them and other people she hasn’t imitated them in front of them, some are not aware, but the rest are, and she's a really funny charming girl. |  |
| CH | Okay, so she helped you by taking your mind off things. |  |
| 13 | Yes I was good in her - we were good friends since the age of 12/13 until now, we're good friends. |  |
| CH | And the friend from college you said at that time you weren’t seeing her much, why was that? Why weren’t you seeing her? |  |
| 13 | I don’t remember, I don’t think - I was seeing her maybe sometimes but I think I was more occupied with the people in there because there were some people in there that I liked, so I was just spending time in them in the girls hostel. So I think there were times when my mind was already occupied and I would go into that and listen to music and watch comedy films, [Participant’s Nationality] films, old [Participant’s Nationality] films and I was occupied there and I was occupied with arguing with the girls, my mind was too occupied with them. |  |
| CH | Okay and when you started to get better and things, maybe since then when things have been improving but they're still hard work to do things… |  |
| 13 | Yes I'm still hearing voices sometimes but I'm not hearing the monkey or chimpanzee anymore, I just hear them say, "Look at her, look at her figure, just look at her, oh no" whatever, things like that. |  |
| CH | Is there anything that friends do now to help you with that? |  |
| 13 | No.  My friend doesn’t know, and my boyfriend knows, he was supportive but I'm not with him now, I ended it with him. The last two weeks I've been ending it, I wasn't with him and then I saw him yesterday but I ended it again today. But he was supportive of me, while I believe that some things were wrong I realised that he was on the internet to his friend in [Country of origin], his friends were in [Country of origin], he's put up this love song on his Facebook site and his friend has written, "Don't listen to such heavy sad songs, everyone lives through what they do, they live with what they do."  And he's written, "Yes I know, I did something wrong myself and I'm having to live with it, don't worry about it." So then I realised that I felt that this means that covered up they're talking about a relationship that he's gone through in [Country of origin] because he's only been here eight months, he came in March this year. So I felt that - so I say to him that - yesterday and today on text and yesterday face to face I said that, "You never loved me, you still love one of your exes and you can't forget them and you only feel sorry for me and that's why you went out with me, you didn’t love me. You might have - you cared for me but you didn’t love me" that's what I think. |  |
| CH | Why do you think somebody would feel sorry for you? |  |
| 13 | Because I said to him that my mum and dad got divorced, I didn’t grow up with my mum I grew up with my step-mum; my grandma looked after me and then my step-mum, my cousin as well. So then I feel like he's seen I haven’t had a stable good life, until this stage I've had a hard life, so I just think he just - he likes me as a person, he cares about me but underneath that he doesn’t love me, even though he says, "I love you and I care about you a lot" and his eyes full of tears when I mention separation, when I say, "I'm leaving you", I still don't trust him, I've lost my trust in people. |  |
| CH | When did you lose your trust do you think? Is that from having the psychosis? |  |
| 13 | That could be part of it or all of it I don’t know, but it's not all of it, there's some more, is that the boy I went out with before this one, he came to me and said, "When I was going out with you I was going out with other girls and now I'm next to you she doesn’t know I'm next to you, I've got a girlfriend now." And then after that this boyfriend, this last one I went out with said, spoke to a girl on the phone next to me in my house saying, "You're the only one, you're different from everyone else, you're the only one in my life" and he said that and then he said he never saw the girl, that it was a girl his cousin randomly gave his number to.  And the girl was in [abroad] or somewhere else and that he never saw her or met up with her, so he says, " when he came to your house you walked outside so I should also not be trusting you if that's the case, why don’t you trust me? Because I spoke to a girl on the phone once, I never ever met up with her; I never lived nothing with her so why don't you trust me?" And I say, "I can't trust you because what that boy said to me before you and after what you did, you spoke on the phone next to me." Now I lost my trust in people, in men. |  |
| CH | Do you think that if you were looking for friends now, if you wanted to make friends with people now you'd be looking for something different in your friends to what you used to look for? Do you want to be friends with different sort of people now or the same as always? |  |
| 13 | I like my friends that I had from secondary school and college but unfortunately they're all working or they're married and they're working at the same time, so I would like to have people in my life that have - that I have a good time with, that make me laugh, that joke about, that make me forget my worries. So I wouldn’t mind making - I would be happy to make new friends but the place of old friends is always different, you can never replace them. |  |
| CH | Yes. |  |
| 13 | If you have memories of them since childhood. |  |
| CH | Yes definitely, it's nice to talk about all the old - when things happened that made you both laugh. |  |
| 13 | Yes and laughter, sadness, tears, joy, all the mixed feelings that I went through as a teenager, that my friend who's married now and didn’t give me her number, she lived through it all with me like I will tell her everything, everything. I would let it all come out I wouldn’t keep nothing inside so it wouldn’t hurt me, I would share with her, I would get advice from her about how to talk with boys, or I would advise her how to be, what we should do, maybe she was the one that listened to me because she only went out with one person and that was the person she ended up getting married with. |  |
| CH | So you really like to be really open with your friends and talk about anything and everything. |  |
| 13 | Yes. |  |
| CH | But that's been harder lately because your friends have been working or married or you find difficult to get hold of and not about? |  |
| 13 | Yes it seems like they're not - they're avoiding me, they like to know that I'm okay, I'm well, but they don’t want to be part of my life, and I don’t know - I can't sit - I can't be able to blame every single one of them for my illness, that can't be the reason they're staying away, I don’t think so. I think they all have their own circle of friends until this age, like the ones who I only used to see at weddings at stuff or at college, now they have their own circle of friends from university and so now they don't feel the need to be close to me. |  |
| CH | Okay, and have there been any more recent friends from sort of - after your friend from college, are there any friends who've come after her? Because that was actually 2002 I think you said. |  |
| 13 | No until now I was friends with her but… |  |
| CH | Anyone you've met since then, any newer friends I suppose? |  |
| 13 | No. At the hostel - at the mixed hostel and the girls hostel there were nice girls, I would talk with them, but then after that I just leave them there, I never swapped numbers with them, I just saw them while we were living in the hostel, I wasn’t hanging out with them outside the hostel. So there were - I saw a lot of - I saw people from all backgrounds, Ethiopian, Somalian, black, white, mixed race, [Participant’s Nationality], mainly that's all. |  |
| CH | All different nationalities? |  |
| 13 | Yes. |  |
| CH | Okay and is there anything you’d like to… |  |
| 13 | So living in that hostel opened up my vision of the world, it made me see different peoples culture, different peoples way of life, so I'm quite happy in that sense that I did move to the hostel. |  |
| CH | Okay and thinking about your… |  |
| 13 | Especially when I was at school I was only hanging mostly around with the [Participant’s Nationality] girls, I wasn’t mixing with the other races much, we were closer with the [Participant’s Nationality] ones because we've got a similar background, same background, so the ones after that I never mix much with, I would talk with them in class but I wouldn’t hang around with them; the white ones, the black ones, the mixed race ones, mainly I was with the [Participant’s Nationality] ones. So when I moved to the hostel - mixed hostel girls and boys and then another hostel, I saw different people, but I never kept in touch with any of them after that. |  |
| CH | Does that mean that your friend who lent you the money for the ticket and who got married, that your step-mum might know her mum? |  |
| 13 | Yes she does. |  |
| CH | Does that mean that your step-mum knows the parents of lots of your friends from school, those [Participant’s Nationality] girls? |  |
| 13 | No. She knows the names of them; she knows the ones on my Facebook who are from the same village as me. |  |
| CH | Right okay. I'm just thinking if she had any impact on how well you've managed to stay in touch with the other people but it's hard to know isn’t it? |  |
| 13 | Yes, I wouldn’t really know. |  |
| CH | Okay. |  |
| 13 | They write to me on Facebook, they say, "I hope you're well, it's good to know you're well", the ones from secondary school, the ones from my village, but I wasn’t close with them I would just see them at weddings, the ones from my village. I never formed friendship with them, they're like acquaintances, I just see them, say, "Hello, how are you?" Talked to them at the wedding and then that's it, I never swapped numbers with them. It's only this girl who bought me the ticket who's my friend who's now married that I was really close with, and the one from college now that I'm close with. |  |
| CH | Okay, and if things were - if you wanted something to be different about your friends at the moment would you like to have more friends or less friends or different types of friends? What would be different for you? |  |
| 13 | Different types of friends and different - more friends. I wouldn’t say less friends because I always have a little… |  |
| CH | Okay so you'd like to have more people around? |  |
| 13 | Yes, I have little ones that I'm in touch with day to day. |  |
| CH | And if you had more friends what sort of thing would you want to do with them? |  |
| 13 | Go to the cinemas, go to nice places and eat together, and watch TV at home together, introduce the person I will be with in my life to them, the person in my life to them, a boy when there is one in the future and make them become good friends, make them have a bond, bond with them, and that's it. |  |
| CH | Okay and would it be friends that you would want - would you want to be able to talk to them about everything or would it be more just for fun times? |  |
| 13 | I would definitely want friends that I would be able to talk with about all my sadness, my joy, my worries, everything. And then I would want, at the same time, to be able to have fun with them, which is what I was able to do with my friend that got married. We could have fun and I could cry next to her and I could tell her about the boys that I've got feelings for. And then when they have feelings for me I'm telling them, I'm pushing them away, I did that with two boys, that was at college, so all those mixed feelings of not knowing what to do, all my teenage worries, I was telling it all to her. |  |
| CH | That's what you'd like again? |  |
| 13 | Yes. |  |
| CH | And would you like to be able to tell your new friends about the voices or is that something you’d keep to yourself? |  |
| 13 | No I would want to keep that to myself because there was a girl that - oh yes I forgot to tell you that, there was a girl from the job centre when we went there many years ago, on the job centre you go on New Deal programme, I saw her and I was friends with her. And then I saw her twice, once I bought her to my house and we drank a little bit of alcohol together and then after that when I told her my boyfriend got angry with me and called me schizophrenic and then she got - she after that didn’t call me. |  |
| CH | Okay, okay, so you think that that was because of… |  |
| 13 | Yes she thought there's maybe some - like this girl is not quite okay. |  |
| CH | Okay, okay, do you think - I mean it sounds like when you've been kind of working hard on your illness and getting better and the work that you're still doing, is there anything friends could have helped with, with that? Do you think friends could have made that easier or…? |  |
| 13 | If I had explained it to them, they couldn’t make it easier, they couldn’t make the voices go away but they could have just been there for me. I never told them about it. |  |
| 13 | I told my friend that got married that I went through depression and that's why the boy in [Country of origin] also possibly didn’t want to know me and she said, "It was your aunts' fault, your aunts shouldn’t have got involved, they just messed it up for you, he really cared about you, he really loved you" that's what she said to me. Because she got to know him, they became friends on MSN, they would see each other, speak, I made her - he's all the way in [Country of origin], my friend's in [city] and they became good friends, they bonded well as well because she was my friend and his friend.  Those days were beautiful, I wish I could go back to the first times when I was - because I never - I only saw him 17 days, but when I - but when I saw him I knew he liked me a lot, he had a lot of feelings for me. But his aunt said the second time I went, she said - his name is [Friend 1], she said, "[friend 1] has got a heart that…" I listened to her from inside, she goes, "He's got a heart that will jump from one girl to another girl, like he will fall in love easily and then he will fall out of love and he will fall in love with someone else" and I never forget that, but I believe he had deep feelings for me, he really cared about me.  But I just want to go back to them days when - you know because I was a bit younger, a bit more innocent and I knew - and I just want to go back to those days and I wish I could make it all okay with him, that's something I'm longing for. |  |
| CH | Okay, okay. Do you think there's anything that people here at the Early Intervention Service could do to help you make friends, to get more people in your life? |  |
| 13 | Yes that would be really good for me actually, I would want to come to the groups - they have here groups once a month, they take them to cinemas or bowling, but I went once to bowling and I didn’t know how to do that, I've never played bowling in my life so I didn’t know how to play it, so then I didn’t take part in it, I just sat with them.  But then another time I went and we played football at the park here with three or four people, I liked that a little bit but we never bonded with the people, we just played the match and then we came home, we left and we went home, so I don’t know, possibly I would like to see new people. |  |
| CH | But you'd like to be able to have some time to talk to them? |  |
| 13 | Yes. I wouldn’t want to give them my phone number and see them all the time, I would want to get to know them and trust them as friends while I'm bonding in here or outside here and then after that I would want to give them my phone number. Only the ones I want to stay as my true friends for all my life, those ones, or even if not all my life, like as long the time we're together they’re good to me, I would want to give them my phone number after I get to know them inside here. |  |
| CH | Okay, and do you think it would be easier to talk about the voices and things to people who also come here, or do you think you would still prefer not to talk about it? |  |
| 13 | I think I would like to talk because they're not my friends already; they're not people in my life so it would be okay to speak with them if they've gone through similar experiences, and I think they have. |  |
| CH | Yes I think you're right; okay let me check my list see if there's anything left I haven’t asked you already. One other thing, what do you think it is about you that your friends like? What you said, you like your friends because they listen to you, give you advice, help to distract you from the voices you hear, all of these things, what is it about you that your friends like? |  |
| 13 | Sorry say again. |  |
| CH | What are your qualities that your friends like about you? |  |
| 13 | I'm funny, I'm charming, I like to joke a lot, I know how to have a good time. I don’t know if I can - I'm not so good at that now because I went through depression as well, now I think I'm quieter and I tell the doctor who writes my medication that I believe that I'm still depressed as well. So now I don’t know how to enjoy life as much as I used to. I used to be able to have a lot of fun and make a lot of jokes and laugh, now I can't do all that because of the bad experiences that have gone on in my life, it made my wish to live - my enthusiasm to live, die down. |  |
| CH | So you think you're a bit of a different person with your friends now to how you were before? |  |
| 13 | Yes it changed me and I wouldn’t - I couldn’t joke, I became more just sitting down serious and then I couldn’t laugh, I couldn’t laugh much at jokes, that's how I've become. |  |
| CH | And do you think that's one of the reasons that you see friends less as well or…? |  |
| 13 | But I'm not like that with my boyfriend who I was with, I joke with him, he jokes with me, we joke, we laugh, we have fun together, but at the same time I believed he didn’t love me. I mean afterwards just recently - that's been a week since he wrote that message on Facebook and I've been believing that he didn’t love me. |  |
| CH | Is there anything else that you would add, you'd want to say about your friendships and how they've been and how they've changed? |  |
| 13 | There was a girl, again from New Deal, [Country of origin] girl, I became friends with her while I was living in the mixed hostel, the first hostel I went to, I knew her from New Deal from job centre. And then afterwards she said to me, "You're rude and you're sarcastic or rude, you're quite rude to me so I think our friendship is not working, it's best we don't see each other anymore." And then she never called me after that, never wondered how I was, never got worried how I was, and then I saw her, this was last summer or summer before and I saw her walking towards the bus stop and I was at the bus stop, she was walking with her mum.  And then I walked away from the bus stop to the next bus stop, so she saw me, because when I went on the bus they came, they were already on the bus when I was on the bus, so then they said - her mum said, "Go and talk to her" and she said, "No, she doesn’t want to talk to me, why should I talk with her, she's walking away from me." And then I bumped into her and then I saw her on Facebook and I wrote a message and then I saw her face to face on the bus again just about a month ago and then she said - two months ago and she said - I said, "Why didn’t you call me after you said our friendship should be over? You never worried about me."  She said, "I was too busy occupied with university and stuff" so then I said, "Okay I want to be good friends with you because I'm not rude to you and it hurt the way you speak" you know when you, what's the word? You answer back in angry way. |  |
| CH | Okay, aggressive? |  |
| 13 | Not physically. So then she said, "Okay" I said, "Add me on Facebook, I'm not the person I used to be, I've become very sensitive to people's feelings and stuff, I've really changed a lot" and then she didn’t add me on Facebook and I'd gave my number for her as well but she never called me. And I went to her house before that and her mum and dad said she's sleeping and I left my phone number with her mum and I don’t know if her mum gave it to her or not but the girl basically wasn’t interested anymore. |  |
| CH | Okay so you tried quite hard to be friends with her and she didn’t make a response to you? |  |
| 13 | Yes. |  |
| CH | Okay, well thank you very much [Participant Name], you've told me loads of really useful information. |  |
