# Supplementary material for: Experiences of friendships of young people with first-episode psychosis: A qualitative study
Source: PLoS One. 2021 Jul 30;16(7):e0255469. doi: 10.1371/journal.pone.0255469 (PMC8323937; doi:10.1371/journal.pone.0255469)
Supplement: S1 File — (ZIP) [file pone.0255469.s001.zip › Transcripts of interviews/Participant 14.doc]

| CH | Okay. Do you have anyone in your life who you see socially at the moment? Have you got friends about? |  |
| --- | --- | --- |
| 14 | Yes. |  |
| CH | Okay, where are those friends from? Can you tell me about them? |  |
| 14 | What, where I met them or… |  |
| CH | Yes, Where you met them, what sort of things you do together, how often do you see them.. |  |
| 14 | Well, I sort of met – I’ve got one friend who’s actually living with us now and I’ve known him since, sort of, 2007, like a year before my episode. We – well we live together, so we sort of hang out together and go out every so often for a drink, kind of thing.  Then I’ve got – I made a really good friend in June, July and she’s, sort of, she’s a single mum, so I don’t see her that much. We, sort of, try and do things together, like go out and eat or have a night in. Then I’ve got some old friends which I had ages before my episode, like five years ago, and I see, sort of, one of them more than the others. I see him every so often, not as often as I used too.  That’s basically it, I don’t have that many – I don’t have a massive group of friends that I - I’ve never really been like that anyway, I’ve never been one to have loads and loads of friends. |  |
| CH | Yes, lots of people are like that. Prefer to have, kind of, a few friends rather than a group of friends. Okay, so the first person, the person you’re living with at the moment, you’ve known him – you said 2007, did you say? Yes. How did you meet him? |  |
| 14 | Well, I used to go out, sort of, to a bar a lot and we, sort of, knew each other through a friend. We used to, sort of, say hi to each other and then I don’t remember really, we just ended up starting talking more. Then we just, sort of, started hanging out together and then we became really good friends. |  |
| CH | Okay, and then the other friends that are older friends, the five or so - perhaps the one that you are friends with, was that the same sort of thing or did you meet them somewhere different? |  |
| 14 | I met – I was going out with someone and, for a couple of weeks, and he lived in like a house with loads of people because he was from [Country of Origin]. I just made friends with about two girls that were living with him and then after they, sort of, separated and they moved into another flat, sort of, with two other friends and then I, sort of, made friends with them.  So, they’re always together, so I was always with them. Then one of their friends moved from [Country of Origin] over, a guy, and I made friends with him very well, really like closely. Then it just - then they, sort of, lived separately and we all still met up and I still visited them all and we all went out. So yes, through, sort of, yes through someone else as well. |  |
| CH | Yes, knowing friends through friends, that’s the most common way I think, isn’t it? How about the newer friends? |  |
| 14 | Through the guy – through my friend that lives with us. Yes, he sort of knew this girl, she’s half [European], half English. I mean, just like, “You should meet her, she’s really nice.” We all met up one day and then we just clicked and we just continue talking. |  |
| CH | Okay. Which of these friends, or if any of them, were around when you were having your episode? |  |
| 14 | The old one, the older ones which was mainly a guy and girl. They ended up – there were two girls and two boys and then one girl moved out and lived on her own with some guy and then the guy, the other guy, moved in with the girl. So, then like a girl and guy and they ended up living together for like two, three years from place to place.  So, I had more, sort of, closer to them two. Then the other girl had a baby, so she was a bit more – like not going out a lot. So, it was them two that were around when I had my episode. |  |
| CH | Did you tell them what was happening to you? Did you talk about it with them? |  |
| 14 | At the time? |  |
| CH | Yes. |  |
| 14 | To be honest I didn’t really know what was happening myself, like I just thought it was normal. So, I didn’t talk to anyone, even my family, about it. I think they knew that something was wrong, that I wasn’t myself and yes.. |  |
| CH | How bad had things reached at the point where it was becoming clear to you that something was wrong? and then maybe you started having treatment from here at the centre and things like that? |  |
| 14 | Well, I got sectioned. So, after about a week or two of being sectioned, being inside, I, sort of, started looking at - like acknowledging and understanding what actually happened. So, it was in, sort of, the mental health centre that I was starting to understand, sort of like, what happened wasn’t normal.  They would come and visit me every so often but I was still a bit, sort of, midway. Like a bit – didn’t really kick into it properly. Then after I came out I, sort of, lost – I don’t know, I sort of – yes, I didn’t - they, sort of – they didn’t – they weren’t my life anymore, sort of thing. Weird. |  |
| CH | Why do you think that? |  |
| 14 | Well, when I came out and I then I, sort of, came, sort of, from one day to the other, like I still had a bit of my episode and I went to [Country of Origin], where they’re from, their village. They’ve got a friend there who now lives here and I asked him if I could come and see him. So, I went to see him and then over there I was acting really weird as well and just - I got arrested by the police over there.  It was like a really small village and it, sort of, brought shame on them. Everyone knew what was happening, all the families know each other, it’s like that in [Country of Origin]. So, then they relayed – they found out here in [city], then they just stopped talking to me. |  |
| CH | Right, okay. Was there anybody else with whom the same sort of thing happened or not? Not the exactly same circumstances but that you lost touch or the friendship ended as a result of your episode? |  |
| 14 | Well, my partner at the time. That ended when I went in to the mental health centre. |  |
| CH | Which bit of it caused that to end, was it the fact that you’d been sectioned or was it the way that you behaved or… |  |
| 14 | Well, when I was in and he came to visit me and I went to, sort of, kiss him and they, sort of, said, “No, you can't. You’re not allowed to.” You, sort of, can't have contact like that with people. Then he never came back to see me again and I think – what he told me was, that after he saw me in there he went to the hospital for two days because he was hallucinating as well, so I don’t know if that’s true.  Then it could have also been his mum was very like preoccupied as well and she might have said something to him like, “Don’t see him ever again.” I didn’t want him to come. I, sort of, before I went in I, sort of, broke up with him as well.  I didn’t want to be with him anymore, so after I came out in February he was just, sort of, trying to get back to me and I was like, “I can't cut it.” So, that was, sort of, my side. It wasn’t like – I had control over it, if you know what I mean. I was the one that, sort of, decided to not – to lose touch. |  |
| CH | What was it that made you just like that? |  |
| 14 | I think that’s part of what led me up to what I suffered, like the main episode. |  |
| CH | Okay, so part of your decision to lose touch is something that might’ve happened anyway. I wondered if you hadn’t been unwell whether you and him, the relationship might have survived? |  |
| 14 | It’s hard to know because sometimes I think maybe he, sort of, brought it out in me. Made me so that I wouldn’t depend on anyone. So, maybe in a way he, sort of, brought this out and, sort of, it came to the surface and then I realised, sort of, what happened.  In a way like maybe it was him doing – me getting better as well. If I’d stayed with him after maybe nothing would have changed, if you know what I mean. |  |
| CH | What was it about him that makes you think that he might’ve - it might have stopped you getting better? Is there something about the way he was or what he did? |  |
| 14 | Yes, just, sort of, I feel like he used me a lot. Like moneywise and just, I don’t know, it just didn’t feel right. It just didn’t feel - like an instinct, it was just an instinct, I don’t think there was any concrete evidence. He wasn’t hitting me or – do you know what I mean? Just little things that make you think, “This doesn’t feel right.” |  |
| CH | So, it sounds like it was a decision that maybe this guy wasn’t going to be very good for you. As you said, he didn’t do wrong or anything concrete but he wasn’t going to be helping you to get better. |  |
| 14 | He used to grow and smoke a lot of cannabis at the time as well. I’m not blaming anyone because obviously you, sort of, you do it, do you know what I mean, you can't be offended. Like he used to smoke every day and I was nearly all the time at his and he would like, “Oh, let’s buy some,” and I would smoke it.  I would be just like – I felt like I had to smoke it because he was. So, yes, I ended up smoking it all the time. So, yes, maybe he wasn’t good for me. |  |
| CH | Was there anybody around who helped you at that time? Either helped you to find support or talked to you or helped you when you were starting to get back on your feet? |  |
| 14 | After I came out from section? |  |
| CH | Yes. |  |
| 14 | You know it’s still quite blurry to me when I came out because I was still quite, like I was trying to fit back into society a little bit, so I wasn’t really going out anyway. No, just my family. |  |
| CH | Right, so you chose to talk to your family? |  |
| 14 | Yes. |  |
| CH | Okay. |  |
| 14 | I don’t think I had – I had the guy who I have now but that was it because then I had lost touch with those friends that I thought were really like close friends. They were really the only people that I used to hang around with. |  |
| CH | You were relying on your family? Are your family here in [city]? |  |
| 14 | Yes, they are. Yes, because obviously I live with them as well and they, sort of, saw me, what happened to me. Mum came to visit me every day I was in the mental health centre. So yes, just basically them really. |  |
| CH | Okay. When you started to, kind of, get life back on track, back to normal, started going out again and things like that, what was that like for you? After you’d been in the mental health centre, started to become, maybe not quite back to normal, back to how you wanted to be, that little bit in the middle… |  |
| 14 | Like the transitioning? |  |
| CH | Yes |  |
| 14 | I used to – yes, it was quite hard but I would try and, sort of, see if anyone wanted to go out, not friends or people I knew, sort of, because I used to know people that I used to just go out – like go out with. I wouldn’t like go to the market or just go out for a drink with them or whatever, sort of, dance like that.  So, I wouldn’t – I used to, I tried to, sort of, find people like that and just to say if you want to go out or if people asked me, “Are you coming out on Friday?” Then I, sort of, I started going out a bit. It was a bit weird being out again, sort of, around people but in the end just slowly, slowly. Just, sort of, slowly, slowly, I just got used to it again. |  |
| CH | Which bit was the weird bit about being around people? |  |
| 14 | I think just grasping the fact that everyone was doing their own thing because, sort of, when I had the episode I was hearing loads of voices and everyone – it seemed they were looking at me all the time and stuff.  So, just looking around when I was out and seeing that people were not looking at me. Not feeling that I was, sort of, being looked at was a bit – because obviously I hadn’t experienced that from before it happened. I think maybe that was the, sort of, weirdest bit. |  |
| CH | So, when you did start going out again? When the part of the illness that was making you feel paranoid had been resolved, so you didn’t have those thoughts? |  |
| 14 | No, because I just felt that that was only when I was, sort of, hallucinating and, sort of, hearing things. It wasn’t like hearing things like, “I’m going to like kill you,” or attacking me, it was just normal people speaking, hearing a conversation. Yes, thinking like everyone knew me, sort of, the grandeur sort of part of it.  Yes, that had been resolved when I was in the mental health centre because obviously they medicated. Then when I came out it was just – yes, just getting back into realising that you’re not, sort of, someone that everyone knows, if you know what I mean. Because that’s what I was thinking. |  |
| CH | Okay, and do you think if there had been friends around when you were under the section and when you came out that had been close to you; do you think that would have made any difference to how things were? Did you miss that at the time or was that – was your mind on different things? |  |
| 14 | Well, because I’d met my partner before it in February of that year and I started spending loads of time with him, so I, sort of, cut my friends off in a way. So, I didn’t really rely on them, I wasn’t really relying on them at that time.  So, when it did happen after I didn’t really think, “Oh, I need my friends here to talk to about it,” because I had my family there and they obviously knew me better than my friends did.  So, I understand in a way how people could want their friends to be there because loads of people might not be in the same position as me, they might not have family to look after them when they come out, do you know what I mean? |  |
| CH | Are you really close to your family? |  |
| 14 | Yes, very close, yes. |  |
| CH | So, you’ve got your mum. Who else is there? |  |
| 14 | My dad and my sister. |  |
| CH | Okay, and they’re all around? |  |
| 14 | Yes, my dad’s very – he, sort of, doesn’t really talk about things a lot. I’m not saying he’s not emotional but he doesn’t really – he, sort of, mentions something and then it’s, sort of like, “[participant’s name], are you okay?” It is just like, “Yeah,” and that’s it, we won't talk it through.  My mum’s very – like worries and talks about things a lot. My sisters quite – she wants to face things but she, sort of, has a threshold that she reaches. Like if she keeps telling you something and you don’t do it, then that’s it, sort of thing. |  |
| CH | Is your sister older than you? |  |
| 14 | Yes, 39. |  |
| CH | Okay. Do you think that the illness made any difference to the sort of people that you want to be around or is the sort of people that you might make friends with the same as its always been? |  |
| 14 | It’s a tricky question actually. I’m not sure because I was one even before, sort of, when I was younger I was very – I didn’t – I was really, like everyone liked me and I liked everyone, I got on with everyone but I wasn’t – I could never really rely on my friends, if you know what I mean.  So, I wasn’t really searching for that perfect friend that I could tell everything. Then obviously friends in my life, kind of, people that have come along that I’ve really been able to like tell everything to and connect with but I don’t think its changed my way of thinking on how - sort of, be careful who your friends are because you like, you never know.  You can know everyone really well but – because somebody.. Looking at what happened and those friends that then just stopped talking to me because I thought they were really close friends, but obviously they weren’t. |  |
| CH | Yes, so that’s quite disappointing in a way? |  |
| 14 | Yes, there was one, a guy I didn’t – when I met him out he didn’t really want to talk to me and he was like, “Oh, I think we should wait a good amount of time until you get better.” So, I waited a couple of months and then he was like, “After a couple of months you can come and we can go out for a coffee, we can talk about it,” so that’s what I did.  He was very like, sort of, was scared of what happened and how to deal with it. He was really understanding and then slowly, slowly we, sort of, a couple of months passed and we went for a drink and then went for a meal.  Just slowly, slowly started building up again with the guy and now we’re really, sort of, close again and we see each other every couple of weeks. The girl she’s never talked to me again, sort of thing, I’ve spoken to her a couple of times on the phone and I’ve seen her once but she totally just cut me off. |  |
| CH | How did you make sense of that? What do you think is going… |  |
| 14 | I think she’s very like family orientated and she, sort of, looks at how everyone, sort of, sees her. She has to look – she has to like represent, if you know what I mean. It’s an [Nationality] thing, like if something goes wrong, like if someone brings shame on you and you’re sort of like, “Oh my god. What is it going to look like.” |  |
| CH | Okay, and she perceived it as you brought shame on her family when you… |  |
| 14 | Yes, and he was more – obviously also, sort of, gay. So, the majority of gay people they don’t really care what people think of them. Really that’s my perception on it because they do their own thing and they just want to be who they want to be and not, sort of, conforming to society, if you know what I mean.  He was more - I think he was more understanding and he’s, sort of, detached from [Country of Origin] a bit more than she did. |  |
| CH | Almost more like open minded, do you think? |  |
| 14 | Yes, more open minded. I think that’s why I, sort of, then I understood more and wanted to build my friendship up again because we were really close. |  |
| CH | Do you think that when you see friends now, you do the same sort of stuff that you always did or – I mean you mentioned like smoking before; do you still do the same sort of stuff or is it different? |  |
| 14 | Smoking I cut off. I do, yes I still drink and stuff with friends and go dancing. Yes, I mean nothings really changed obviously apart from the, sort of, manic stuff that I used to do when I was - during an episode. Like crazy stuff. |  |
| CH | How about the sort of role that you play in friendships? People that I’ve spoken to say they’ve seemed to be a particular person in their friendships.  Like with some people they’re the joker or maybe like the one who listens to all everyone’s problems, you know, all different, sort of, roles. I just wondered if that’s changed since you’ve been unwell and become something different? I don’t know what sort of roles you think you play with your friends? |  |
| 14 | Is this just friends or family as well? |  |
| CH | Friends. |  |
| 14 | No, I’ve always been someone to, sort of, listen to people but then I’ve also, I’ve always, sort of, the people that I’ve listened to have always listened to me as well, if you know what I mean, friend wise.  So, I’ve always found people that can, sort of, give back what I give, if you know what I mean. If it’s someone that I talk – that I listen to a lot and then when I’m talking they don’t, just don’t, they’re not really going to be my friend at the end of the day.  No, it hasn’t changed. I always joke around people and that hasn’t changed either. |  |
| CH | Okay, and is talking to your friends quite openly and being able to share stuff quite important to you; that, kind of, openness? |  |
| 14 | Yes, maybe not all my friends, there are specific ones who I tell everything to and then there are obviously some things that I don’t tell anyone. Maybe just things for me - like if I’m feeling a bit manic, obviously I’d tell someone that but other stuff maybe, feelings towards someone.  Maybe I might not tell that person that thing because it might, sort of, affect them. Yes, I say what I think as well, if I think it’s wrong. |  |
| CH | Yes, and is that ever affected by your paranoia, for example, saying what you think? |  |
| 14 | What do you mean? |  |
| CH | Well, some people have said that if they’ve experienced feeling paranoid about people, they found it more difficult to talk openly with people because they worry that although they are not feeling paranoid anymore, that there's still, kind of, a slight bit of mistrust or concern about… |  |
| 14 | I used to do that during an episode. |  |
| CH | Yes, anyone really who – I don’t know about your symptoms, I haven’t read any of your file, so I don’t know whether you were feeling paranoid about, sort of, specific people or specific places but sometimes if people have been paranoid about specific people they found it difficult to relate to them when they felt well again. Do you see what I mean? |  |
| 14 | Right, I understand. See I, sort of, it wasn’t a, sort of, target paranoia, I’ve got more general. So, there's maybe a few crumbs along the road as well, like it wasn’t brand new thing.  I didn’t think, “Oh, that persons talking about me.” Then I think if that would have happened, it would have been - I would have – like the girl one that, sort of, obviously after it happened I obviously thought she is probably better off without me, like around as a friend, because then some of her friends that I knew didn’t want to talk to me anymore either.  Then I just basically cut her off, not that I didn’t care but I just thought, “Well, sod off,” sort of thing. So, I wouldn’t have been that much affected by it, if you know what I mean. It wouldn’t have affected me that much but I understand that some people it might affect. |  |
| CH | Okay, that’s fair enough. Thinking about now when you’re at home, is there anything that you think that’s missing in terms of your friends or your social side? |  |
| 14 | I miss going - like I miss going out and just talking to loads of people and knowing I’d be walking past them and then, “How are you?” sort of like knowing them like that because I haven’t done that for ages. |  |
| CH | Why is that? |  |
| 14 | I don’t know, I just started thinking maybe I shouldn’t be that known to everyone because I’m quite a – I was, sort of, quite – I was sleeping around a lot as well. I just thought maybe everyone knowing you isn’t, sort of, a good thing actually.  They might be – so yes, it’s maybe the knowing – like the gay scene as well, everyone knows what you do and you sleep with someone and they know, and the person you know slept with them. So I, sort of, felt that maybe I should not, sort of, do that anymore. |  |
| CH | So, you decided to, kind of, get a bit of a lower profile really? |  |
| 14 | Yes. Obviously it’s a bit of a confidence thing, like everyone knows you, it’s like, “Oh-o,” but then obviously that can't go on forever. I miss also, sort of, getting drunk and not having to think about anything or think about your actions, which is obviously what got me here but do you know what I mean? |  |
| CH | That, sort of, carefree kind of thing? |  |
| 14 | Yes, and, sort of, not having to, sort of, walk down the road maybe, be careful, like, sort of, like want to walk in – like say there's a group of boys walking towards you and, sort of, having to cross the road because I never used to be like that, I was very - like I didn’t care, sort of thing. |  |
| CH | Why do you think that changed? |  |
| 14 | Well, I got mugged, so that’s probably caused it as well. I don’t know, maybe having to – realising that I can be that way. I can be carefree but then I have to be, sort of, be quite careful what I do as well. So, that it doesn’t just lead me off the path of where I want to go. So, just to get a direction in life, it’s difficult to explain. |  |
| CH | Yes, I know what you mean, it’s, kind of, all a bit… |  |
| 14 | Grown up, sort of thing. |  |
| CH | Yes, and having something which makes you have to, kind of, medication and think about things. It does make you grow up quickly and then they have to – they lose the carefree teenager, 20s bit because all of a sudden things are a lot more… |  |
| 14 | Yes, because that’s the thing, because I had to take medication at the beginning of 2008, I had HIV. So, then I started in 2008, February, so since then I was like ten months before my episode.  So, I started, sort of, I already had that, sort of, like take every day my medications, I needed routine in my life, I can't, sort of – yes, I can't just not care – I can't just be carefree anymore, I have to, sort of, care for myself. Then in the end I got with a guy and just went a bit off the rails. |  |
| CH | Okay, last couple of things. If you’re looking for friends and people to be in your life at the moment, what would be the most important things about them? What sort of qualities would you – do you want in the people that are around you? |  |
| 14 | I wouldn’t like all my friends to be the same, I would like – obviously I would like positive people but then I would also, sort of, like people that are quite, sort of, negative, sort of like a balance.  So I still know that you can be happy but then you can also have your problems and be sad. I like having friends that can tell me their problems and I can, sort of – what's the word, not guide… |  |
| CH | Support them. |  |
| 14 | Yes, advise them of what to do but then not to a point where I’m doing too much, where they’re relying on me a lot which what was happening before I went in. My family was always listening to problems, like family problems and I was like, “Yeah, yeah, yeah. Maybe you should do this and do that.” Then I was, sort of, keeping everything inside.  So, I would like to have friends that but then also that I can talk to about – that listen to me as well and advise me as well, maybe not tell me exactly what to do but, “Oh, I think you should do that,” or, “I think you could do things a different way.”  Think things through and then yes, friends that I can also go out with for like a meal or a drink. Yes, I need to stay away from friends that go out all the time, every weekend they get drunk and pass out. |  |
| CH | Do you think that’s a result of being unwell or do you think it’s what you probably would have done anyway, changed as you’ve got older? |  |
| 14 | I don’t - it’s quite, it’s a, sort of, gay thing. It’s like you go out and you start going out when you’re 16 and you start drinking and then you start sleeping around and you have to look good all the time. I think it was what I was entered into, if you know what I mean.  This, sort of, trying to separate yourself from everyone else. Nowadays I think it’s very much more – everyone is getting integrated with each other and obviously back when I started going out it was, sort of, quite integrated but it wasn’t as much as it is now I think.  You’ve still got the kids that, sort of, want to - they go out on the gay scene and they want to fit in, in a way. So, yes, I think yes, it would have happened anyway. |  |
| CH | Maybe it is something about like finding your identify and getting used to who you are. Who you are has become much more than just the gay thing, I don’t know. |  |
| 14 | Yes. |  |
| CH | Did you ever come to anything here at the centre, I mean the groups or the social stuff? |  |
| 14 | Yes, I did one of them, I think it was like – they had like snacks and stuff, when I was sectioned. No, I haven’t been to any of the – okay, I don’t want to, sort of, like discriminate against mental health but I don’t want to be labelled as like someone that has – do you know what I mean?  I don’t want to be like labelled as a mentally ill person, so I don’t – I really don’t like all this social, sort of, going out with people that have gone through what you’ve gone through. I don’t know, it’s a bit weird, not being horrible but do you know what I mean? |  |
| CH | Yes, it’s been really interesting because it’s been like two groups in a way. So a group who’ve said similar to what you said, actually I don’t really want to be reminded of what I’ve gone through.  Then there's another group of people who’ve I really wanted to talk to somebody who has been through the same thing that I have. |  |
| 14 | I think it’s also like, maybe some people that have gone through it they come out with an understanding of why it happened and they’ve been able to talk to people and they’ve been able to find out why it happened.  Obviously on the other side there are people that haven’t been able to do that and they don’t know why it happened to them and they are still, sort of like, “Why did this happen to me? It has ruined my life,” do you know what I mean.  I think those are the people that maybe want to integrate more with people that it has happened to because they want to find out what they went through to get to that stage as well. I’m always trying to look at the positive things that happened, even if they are bad stuff.  I’m always trying to find the positive in them. Yes, in a way I, sort of, move on. I don’t want to be around people that remind me of what happened, do you know what I mean? |  |
| CH | Yes, that’s fair enough. Do you ever talk to [care coordinator’s name] about social stuff, friends and stuff like that, or does that not really ever come up? |  |
| 14 | She’s always going, “Do you want to do this group and that group,” and I’m like, “No, not really.” She does tell me like, “Do you go out, are you going out with friends and what do you do with the friends and stuff,” yes… |  |
| CH | She checks? |  |
| 14 | Yes, she checks on me, sort of, If I’m going out, like if I am going out and stuff, like if I’m actually meeting a lot of people. I think I would tell her as well if I wasn’t feeling - do you know what I mean? |  |
| CH | So, if you were starting to feel a bit deflated or less confident you’d feel able to say something to her? |  |
| 14 | Yes |  |
| CH | Okay, do you think there's anything the service here could have done differently to help with anything friendship wise when you were recovering? |  |
| 14 | I would have liked maybe like an hour, so when I was, sort of, getting back into home life and stuff, maybe someone – I know this is probably hard because there's not much research into it but someone who had gone through what I had and maybe, sort of, on the sidelines more.  Sort of, if that person understanding what had happened, maybe, sort of, mentoring, that would be good. Come round once a week and, sort of, just talk to me, not, sort of, ask – do you know, just, sort of, maybe helped me in a way but not like, “Do you feel like this?” like a doctor would or a social worker. Someone that could just, sort of, buddy you and then maybe get things out of you that questions and answers couldn’t.  Like obviously [care coordinator] is really good and everyone else is really good, all the doctors, but they’re very like, “This is what happened and this is what we have to do to make it not happen again.” It would be good to, sort of, find someone that had gone through it and just maybe talk to them about it.  Ask them more things because I understand that they’ve seen loads of people who’ve gone through it because they’ve helped people but when it’s happened to you, you don’t know until its happened to you what it really means, if you know what I mean. You can study and study and study forever. Yes, a buddy I think. |  |
| CH | Yes, I think that’s a really good idea. Some of the people that I’ve spoken to while I’ve been doing this, have wanted similar things but haven’t put it as eloquently as you’ve just put it but I think touching on the same sort of ideas. |  |
| 14 | It’s obviously very hard. You’ve got like people who just want to forget it and don’t want to ever be in touch with the mental health services again because maybe, I don’t know, they’ve had a bad experience or something and don’t want to remember it.  I think there's obviously not that many people that could do that because there's so many people coming out of hospital and stuff, it’s hard to get one person to do everything.  Yes, someone that could do that, even one person that could maybe visit two, three people a day, different people a day, Monday to Friday or something. So, they have their like 12 people a week that they work with, pay them really well. |  |
| CH | Loads of money! |  |
| 14 | Yes! I would - if that ever comes into action I would be happy to do that, if they ever asked me when I’ve, sort of, sorted myself out. I wouldn’t mind like helping out. |  |
| CH | Okay, is there anything else about friendship or social stuff that I haven’t asked you about that you thinks been important in the last couple of years for you? We’ve covered quite a lot. |  |
| 14 | Just maybe – yes, no not really. |  |
| CH | Okay, thank you. |  |
