# Supplementary material for: Experiences of friendships of young people with first-episode psychosis: A qualitative study
Source: PLoS One. 2021 Jul 30;16(7):e0255469. doi: 10.1371/journal.pone.0255469 (PMC8323937; doi:10.1371/journal.pone.0255469)
Supplement: S1 File — (ZIP) [file pone.0255469.s001.zip › Transcripts of interviews/Participant 2.doc]

| CH | I’m interested to hear about people in your life who you see socially, people you consider to be friends. Have you got a specific friend in your life at the moment? |  |
| --- | --- | --- |
| 2 | To tell you the truth, when it comes to friends my parents always told me growing up that you keep your enemies closer than your friends, so I don’t really have that much friends. I’ll say that my mum is one of my nearest and dearest, and I’ve got a two year old son so I’m working on our relationship so we have a good, just as good a friendship as a relationship. I’ll say one of my best friends are my mum. |  |
| CH | Ok, has that always been the case? |  |
| 2 | Yes, definitely. I think since I had a son I’ve wanted to have more of a bond with my son, create more of a relationship so he can talk to me as if I was his friend. I would like the same relationship that I’ve got with my mum right now, to have with my son. I look up to my mum and if I have any problems I always go to have a chat with my mum. I speak to my mum very regular and often daily, so I’ll say my mum is one of my nearest and dearest friends right now. |  |
| CH | So the important thing about your mum in terms of being your friend is that you talk to her regularly. What else is important, what makes that such a good relationship? |  |
| 2 | If I have any problems like personal problems, or questions, sexually or anything, abusively, not abusively; anything like I’d like to ask questions about or that I have concerns about I always go to mum, because my mum like she’s in her forties and she’s pretty experienced, so I can look up to her and ask her a lot of things and I can get a good reply, a good response. |  |
| CH | So she gives you some pretty good advice? |  |
| 2 | Yes, lots of support. And she phones me pretty regularly so I know that I’m on her mind and she cares for me a lot, my mum. |  |
| CH | Is there anybody outside of your family that you would spend time with? |  |
| 2 | Socially, I have an auntie, and my son is in local authority care right now, and that’s.. If my son was with me right now, I’d definitely be saying that that’s my best friend, even though he’s a two year old. He’s like a teddy bear, something that you can cuddle and squeeze, you know what I mean? Someone you can teach a lot you know, who can look up to you, because I’m teaching my son a lot right now, he speaks two different languages and he’s only two and a half. He looks up to me. But also my auntie who lives in [area], my mum’s half sister, and she’s a very close friend. Other than that I’m in a relationship with my baby father, and he’s more a friend to me more than anything, so he can count as a good friend. But after I left school I lost contact with lots of my friends. If I do speak to any of my old school friends it’ll be on facebook, that’s it. |  |
| CH | Why do you think you lost touch with those friends? |  |
| 2 | Because I moved far out from the area, a good couple of miles, so I lost contact with all of my friends unless I were to go and visit my grandparents, [then] I would occasionally see them. Its something that.. It’s a good thing that you’ve just brought up to me actually, because friends is something that I’m actually, I’m pretty interested by this whole case of friends because friends is something that comes and goes. Its something that if you want a good relationship or friendship, shall I say, its something that you’ve got to see, you’ve got to take it in your own interests to get to know that person, because if they don’t know then you can miss that opportunity. Friends is something that comes and goes but not very regular. |  |
| CH | So if you see a chance you’ve got to grab it? |  |
| 2 | Yeah, it’s very hard to come by a friend, because there’s a big difference. There’s someone you can call your friend but you don’t really know what they’re a good friend or not, so it’s something that you don’t really get, don’t come by that often. You don’t really meet someone and you’re like ‘oh you’re my friend’, it takes a lot of tests to pass before you can say that person is a really good friend. |  |
| CH | What sort of things would those tests be? What would you be looking for in a friend? |  |
| 2 | Someone very smart, someone intelligent, someone that’s going to put you in the right positive direction and not negative. Someone that is good. It’s good to have friends that are you’re age group, not older, not younger, but in the same age group. Someone that you can relate to and if you do want a break, someone you can always go and meet and have a chat with, go out for lunch, someone to talk to. But friends is something that is hard to come by, say like if you are my age, a lot of my friends are younger than me so if I was to invite my old friends round.. I was an offender a couple of years back, so a lot of my friends are offenders and thieves and weed smokers. I choose to stay away from those people because if they was to come to my house I’m a lot more older than them, a lot more responsible than them, so its not the type of people that I choose to stay in contact with because they probably thief from me. They’re not such a good friend as that, that’s why I don’t have them kind of friends anymore. |  |
| CH | What happened to stop you being friends with them? What changed? |  |
| 2 | I was put on an ASBO, and I was kicked off my estate. Me and my mum had to move out, because I had a big ASBO for graffiti and robbery etc. I was known by the police station. The whole estate had basically written a letter and people signed it to say that all of my friends and me are making the estate a not very pleasant place. There’s graffiti everywhere, we been smoking on the blocks at night, there’s people being robbed, so we basically were voted off the estate, and I had an ASBO. Not long after that I was put on a curfew for six months then afterwards I had a 16 months supervisional order and I was given an advocate, and that was like a probation order, that I was sent back to court in 16 weeks to see how I performed, and if I didn’t have progress I would have been sent to prison, to a young offenders, because I was uncontrollable. I never had no positive friends that I could rely on, so those people I had to break away because there was no way I was going to be this age and still be on the outside. I would be in jail by now if I was with them same bad people. |  |
| CH | You made the decision for your own future that you were going to stay away from those people? |  |
| 2 | Yes, and not long after I gave birth to my son. I was like, getting in trouble with the police and all that five years ago, so I’ve made a big difference since then. |  |
| CH | Yes, big changes |  |
| 2 | Big, big changes. When I see them people now they can’t even remember me so it wouldn’t make a difference. If I was to see them they can’t even remember me, I look so different. |  |
| CH | Thinking back, to before you used the services here, do you think that there are any difference in your friendships? |  |
| 2 | Its brought me and my boyfriend a lot more closer, because he had to come and see me up in the hospital and stuff. It brought me and my boyfriend to have more of a better.. Just as.. Well as a better friendship, as a better relationship. |  |
| CH | What was it that he did or you did that meant that relationship survived? |  |
| 2 | He showed me that he was there for me, because he used to come and see me up the hospital twice a day and stuff. It really showed me who is my real friends, because not even my mother, that I said that’s my close friend, she never made it up the hospital not once to see me. My boyfriend used to come up there every day. When you go through stuff like that and you end up in hospital and stuff, and no one comes to see you, you realize who are your real friends, who actually come out of their way, they come to see you. |  |
| CH | Do you think, you said before that the thing that you look for in a friend, do you think that they’re different now because of all your experiences, to what you might have looked for before? |  |
| 2 | I don’t know because, it’s hard to say when you’re looking for a friend. If I was looking for a friend I’d just be looking for the normal things, like as long as you’re not getting yourself in trouble with the law, and you’re doing things in a legit way, then I’ll have an interest in you. If you’re a bad person and you’re like getting yourself in trouble with the law, I have a child so it’s not really in my interests to be associating with that sort of person. I’m not really on them sort of wave lengths. I’m more on a positive vibe, so to have a friend and they’re saying ‘lets go and rob that man round the corner’ I’d be like ‘nah mate, don’t want to know’. I’ve got a two year old son that’s looking up to me and I don’t want him putting me down. He’s in local authority care so it’s even worse. |  |
| CH | So at the moment there’s a lot of pressure on you to keep being positive? |  |
| 2 | Yeah, positive. |  |
| CH | The friends that you have around you already, is it important to you to be able to talk about the symptoms that brought you here, to talk about your experiences or do you prefer to keep that inside? |  |
| 2 | I keep that to myself actually. I have a friend that’s 26, she’s [friend 1], she’s a few years older than me, and I find that’s somebody that.. Even though she is older she looks up to me because she lives in a hostel and stuff and I had my child before her. Me and her can relate in a lot of things because I can look up to her in working. She’s in college at the moment and she works, so if she ever wanted to come to me about children, or speak to me about, I can always tell her advice about children and she can always give me advice about work. |  |
| CH | It’s important in your relationship there then is to be able to help each other? |  |
| 2 | It’s good to have friends in different age groups, someone that’s older and someone that’s your age group so you can always see things from a different point of view. |  |
| CH | What sort of things do you like to do with [friend 1], or your partner? |  |
| 2 | [friend 1], a lot of the time, I don’t see her that often but the majority of the time we go for walks because she’s got 3 dogs, so majority of time I’ll come over and I’ll walk one of the dogs while she walks 2 because she’s got 3 dogs. A lot of the time we go for walks to walk the dogs and stuff; she’s got 3 so I can help to keep them under control. |  |
| CH | So when you’re out you can have a nice walk, some exercise but you can also talk? |  |
| 2 | Yeah. Used to be we were on the same estate, but things are a lot more distant between us now. |  |
| CH | Ok. She was somebody that you’d known for quite a while? |  |
| 2 | Yes. I used to be pretty close with her cousin but because I moved off the estate we don’t see each other as much anymore so it’s a lot of phone contact. And if we do make phone contact then we might meet up on that day or something but it used to be that we lived on the same estate, but things have changed. |  |
| CH | You said that you’d lost contact with a lot of people off of the estate, but [friend 1] is somebody that you managed to stay in touch with? |  |
| 2 | Yes, because she’s not like that. She’s always been college and university and stuff. Very positive girl, African girl. |  |
| CH | Because she’s focused and working hard.. |  |
| 2 | I always choose to stay in contact with her. I did lose contact with her when I was pregnant as well but I got back in contact with her after I had the baby. |  |
| CH | And during the time that you spent in hospital, did you lose contact with anybody then? |  |
| 2 | I didn’t really intend to tell anybody because it was my own personal business. I don’t really tend to.. A lot of people knew what was happening but my mum’s sister, aunties, cousins.. I have a very big family, but people like [friend 1] they didn’t notice that I was in hospital. |  |
| CH | So as far as they’re concerned, they don’t know about that at all? You kept that separate? |  |
| 2 | Yeah. |  |
| CH | Did you find it hard to start friendships up again when you came out or was it, did it work quite well? |  |
| 2 | Yeah. I even made a friend inside the hospital actually that was going through the same. A [Asian Nationality] boy called [FRIEND 2] that I’ve stayed in contact with since last year when I was first in hospital that I’d met. I met him in hospital and I’ve been in contact with him ever since. |  |
| CH | What is it about him that has worked out? |  |
| 2 | When I got discharged, he was still left in there for a good 6 months. We stayed in contact and when I was back home I used to phone him at the hospital and stuff and used to go down there and bring him lunch and stuff, just like how my boyfriend used to look out for me. I used to make sure that.. You know it’s not nice when you’re in that hospital and no one comes to see you. So I used to go up there and bring him food and stuff. |  |
| CH | What did that do for you? I can see that he got a visitor and somebody to see.. |  |
| 2 | It made me feel proud of myself for caring, because when I was up in the hospital I used to think that my mum might come up there to see me. I was there for a good two months and I didn’t get to see my son not once, in two months I didn’t get to see my son. And that was really, really bad. |  |
| CH | That must have been really difficult. So you were thinking that you knew what it was like to be waiting there and no one comes, so you’ve been doing that for [FRIEND 2]? |  |
| 2 | Yeah. |  |
| CH | Is he still in hospital or is he out now? |  |
| 2 | No. |  |
| CH | Have you stayed in touch? |  |
| 2 | Yes. I’ve known him for more than a year now. |  |
| CH | And what sort of things do you two do when you see each other? |  |
| 2 | I’ve never met him on the outside; we just make phone contact since he’s gotten out of the hospital. He went to [Asian Country] because he’s [Asian Nationality] and he’s been in [Asian Country] for about a month now so I haven’t had contact with him for a couple of weeks now. He’s on holiday. |  |
| CH | Yeah. |  |
| 2 | He only came out of hospital early January so he was in there for just under a year. He was sectioned. |  |
| CH | Ok. So we’ve heard about your mum, and your aunt and your partner, and [friend 1] as well, and [FRIEND 2], is there anyone else who’s really important to you? |  |
| 2 | My son. |  |
| CH | Of course, yeah, sorry. |  |
| 2 | Yeah, my son. When you have children it’s more, it’s a different relationship. You can ask a lot of parents who’s your best friend and they’re going to say my son or my daughter. Its hard to say because my son’s only 2, so right now like, everybody, my mum, my dad, ask everybody, my son is my best friend, because he’s someone who that, out of all the people in the world right now there’s one person that I can say really needs me, and that’s my son, and he’s in local authority care, and the carer always tells me that ‘he always asks for you’. I’d say that’s my best friend right there, I’m trying really hard to get him back. |  |
| CH | And it sounds like your friendships with [FRIEND 2] and also with your son it’s important for you to be needed and to be able to give something to those people, is that right? |  |
| 2 | Yeah. I love my son very much and because he’s 2 I’m finding it very hard right now to explain to him what’s going on. I see him at this contact centre for 3 hours a week, 2.5 hours twice a week, and when it’s time to say goodbye he starts crying, and it’s pretty sad because he always leaves me in tears as well but I don’t know how to explain it to him. Because he’s only two he’s learning words and stuff but I sometimes sit down and speak to him and say son don’t cry, mummy’s going to be back on Thursday, today it’s Tuesday and its an hour and a half but I’ve got to go. I’m finding it very.. I’m getting very caught up because since I’ve got out of hospital my son’s.. I haven’t had my son and I’ve got the house and everything, and my son’s on the tenancy agreement and sometimes I feel like what’s the point in me being here if my son ain’t here? Very bored. I feel like my parenthood has been taken away from me. I’ve had a very bad two years, back and forth to court and social services. |  |
| CH | Have you felt supported in that time? Have you felt that there’s people around you? |  |
| 2 | Yeah, I guess so. |  |
| CH | Is there anything else anybody could have done to make it less stressful for you? |  |
| 2 | Not really, no. |  |
| CH | We’ve talked quite a lot now about different friendships, I’m just going to check that there’s nothing we’ve missed.  We’ve talked about friendships that you’ve got at the moment and what you’re enjoying from them, talking to each other, being able to give something to other people and feeling that support. Do you think before you were in hospital there was anything you enjoyed about relationships that you’re not so bothered about now? |  |
| 2 | Not really because I’m more.. I don’t really have time for friends because I used to have my little boy with me all the time. It’s not like I was unhappy, because I never had time with friends because I was stuck with my son all the time, it’s that that was what I chose to do. To be a full time parent, not to be careless and to be running around the streets and drinking and going out Saturday and Friday. I chose to be a full time parent. Now that my son is not around I’m looking for more friends, more of a friendship now because I have so much time on my hands. |  |
| CH | And would you want to be doing those sorts of things again? Going out? |  |
| 2 | Not really, it doesn’t really interest me. I made plans to be a full time parent and I’ve had that taken away from me because my son’s in local authority care and now I don’t really think of friends really. I just think of friends when I don’t have nothing to do. It’s not because I want to be with my friends because my son’s not around, it’s because I’m bored. |  |
| CH | Ok. And is there a gap in what you want from friends at the moment? Is there something missing? |  |
| 2 | No, it’s not really in my interests. I’m just trying to do my best to get my son back. |  |
| CH | So you’re attention is on that, you’re not noticing the other things apart from when you’re bored? |  |
| 2 | My baby father’s around a lot of the time so he works in the day time so by 5 or 6 I’m alright. |  |
| CH | It’s the day times when you’re by yourself. Do you think having those people around affected how quickly you got better? Or, do you think it made no difference? |  |
| 2 | It didn’t make no difference. My privacy, me and my son is personal like. Sometimes my baby father might think I’m being a bit soft but my baby father is not on my tenancy agreement. He doesn’t help me with no bills, I don’t ask. My son’s on the tenancy agreement, he’s not on my tenancy agreement, he doesn’t live with me. I think sometimes I could say that I’m being a bit selfish, but if my son.. I don’t know, I keep myself to myself really. Everything’s like me and my son really. And because my son’s not here right now, my baby father’s maybe pushed himself onto me a bit more because he knows that [son’s name] not around so he gets a bit more attention off of me. Before I was a 24/7 mother to my son and I didn’t really make plans to have more children. |  |
| CH | So before there wasn’t anything missing in your life because it was revolving around looking after your little boy, and now your life is still about that but then there’s this extra time until it’s resolved? |  |
| 2 | It’s not just that, I’m like.. My son.. The reason why I say my son’s my best friend is because I had such a shit childhood. I’ve never been taken on holiday by my parents, so I planned on giving my son everything I haven’t had. Right now I’ve been decorating his room for when he comes back. I’ve got like a car bed and everything and new plasma and everything; he’s only 2 now. I plan on traveling with my son, taking him to Paris, Disneyworld Paris and stuff, that’s why I reckon I can relate to a child a lot because I’m very young acting and I like to have fun. I reckon me and my son can relate; I can be really close to him. I can make my son really close. I can become really close to my son; he’s only 2 now and we’re already close. I reckon that as he gets older he’ll be able to come to me and speak to me about anything ‘cos we’ll have so much of a bond. It’s not only that I plan on making time for my son; I plan on going places, taking him abroad and stuff. |  |
| CH | So you’re putting a lot of time into thinking how that’s going to be at the moment? |  |
| 2 | I want him to say that my mum.. I had a very fun childhood. I never had a fun childhood, I witnessed a lot of stuff when I was younger with my parents; violence, parents split up at a young age. I don’t want that for my son. I want to be best buddies with my son. |  |
| CH | Just thinking if there’s anything anybody here could have done? It sounds like the time you were in hospital didn’t have too much impact on the people around you because you managed to keep it private and when you came out you could start your friendships up again quite easily? |  |
| 2 | Well the first time I was ill I was there for three months and the last time I was there for a month. I used to sleep a lot, the time used to fly by in there. I got discharged. I got sent home for the weekend and I went back on Monday and I was discharged, and I haven’t been back there since. That’s how I lost my son in the first place, because I was in hospital. I never want to go back to that place because it doesn’t help. |  |
| CH | Do you think there’s anything that friends around you can do to help that? |  |
| 2 | Nah. My friends are a lot younger than me so they’ve never paid a bill in their life so I can’t really relate to any of my friends. They don’t have a clue. They live with their mums, all of them. All of them. Even [friend 1] still lives with her mum and she’s 26, so I’m a lot more mature. A lot of my friends come to me with problems, because I’m the one with the 2 year old. I’ve lived by myself since I was 14 so I’ve gone through a lot. I could relate to other people who’ve gone for counseling. I’ve been through so much. |  |
| CH | All these people come to you for support and you give a lot to all these people. Where do you get something from? |  |
| 2 | My baby father. And I get a lot of cuddles form my son, and my son shocks me everyday. He comes and tells me ‘I love you’, and he’s only 2 years old. I know that no matter what happens.. My son.. I’ve got a son out there and he loves me very much, and he doesn’t have a clue what’s going on around him. I’ve got so much to make up to him, so much to make up to him. I might end up going to temporary mothers and baby unit so I can be watched over, and I’m willing to do whatever it takes right now, because me and my son have never been separated before and it’s really hard for a woman to go through that, when your child’s been taken away from you. I believe that I’m doing pretty well for someone that’s supposed to be mental. Know what I mean? |  |
| CH | Who told you that you’re mental? |  |
| 2 | Well I’m on this medication called olanzapine, and I think I’m doing pretty well, for someone who’s supposed to have all these mental reactions and stuff, I think I’m doing pretty well. I can’t see the mental in me at all. |  |
| CH | From what you said, it sounds like you’re somebody who from an early age became used to looking after yourself. You’re kind of independent, so you don’t rely on friends or people around you much. You deal with things yourself. Have I got that right? |  |
| 2 | Yeah. That’s why it’s killing me right now. My son’s with somebody else and he keeps on getting flu and stuff, and ok if you foster a child, but do you love my son like I love my son? Are you going to be checking his temperature every 2 minutes? I’m just a bit worried right now that even though he is learning, this person that’s caring for him, he’s not getting the full attention a 2 year old needs from his mother. |  |
|  | So you’re finding it hard to rely on someone else to do something for you? |  |
| 2 | Yes. |  |
| CH | With your baby father, can you rely him? |  |
| 2 | I can rely on him, I trust him, but its just the thing that he’s in neither of our care now. He’s in local authority care right now. Neither of us have a say. No matter, social services deal with everything. I can’t make any decisions. I asked them if my son can come and stay the weekend with me, they told me no, because he hasn’t got a bed and stuff, so that’s why I’m spending a grand on decorating his room right now so when he comes back to my house everything’s decorated for him, his bed .. |  |
| CH | Does it seem like hard work to be the one who has to give? |  |
| 2 | It’ll probably seem like hard work for me because every single time that I seem to be making my way up I get put 5, 7 steps back behind. Like I’ll just start to decorate and the police come to my house and the nurses and I get dragged back to the mental health hospital and then after I lost my son, afterwards I’ve been put steps back, and I’m having to go to court and before he was in local authority care for a month, and now he’s in local authority care with a complete stranger. So you can say that its going to be hard for me, I’m not finding it hard with taking care of him, I’m finding it hard that he’s not with me. And that if it wasn’t for my son I wouldn’t have that house. I would be living in a woman’s refuge. When I got pregnant with my son I got that house, and now I’m living there my son’s not even living there with me, and he’s named on the tenancy and everything. So I’m finding it.. I’m getting all vouchers for healthy start, for vegetables and stuff and I’m buying all this fruit but my son’s not here to eat the fruit with me so I’m finding it a bit lonely at the moment. I don’t really feel like a mum. To be quite honest with you it feels like I’ve had my motherhood taken away from me. I see people in my street and they’re like ‘where’s your son?’ I don’t feel like its their business for me to go ‘oh, he’s in care right now’, so I don’t say nothing. None of my friends don’t really know because it’s pretty serious. It’s not pretty, it is serious. I don’t sleep at night sometimes, because I don’t have my son. I feel like I’ve had my motherhood taken away from me. I was happy up to 6 months ago, I’m not happy no more. |  |
| CH | This is something that you find hard to trust anyone with, to tell anyone about if you don’t trust other people to know about this. Would you like to be able to trust anyone with this? |  |
| 2 | I speak to my mother, I speak to my social worker, and I speak to my solicitor and stuff. I do speak to people, but there’s only so much help that I can get. It’s all down to the judge really. |  |
| CH | So people around you, you don’t want to trust them into your life? |  |
| 2 | I do trust them into my life, its just that there’s only so much that they can do. It’s in my interests to either get involved with these people or if I just sit and wait for them to phone me, it’ll go quiet. If I don’t show them these people that I want to get my son back, what are you going to do to help me? Then I won’t get my son back. |  |
| CH | So you’re taking a very proactive approach.  We’ve talked about a lot of things, and you’ve given me some useful things to think about. |  |
| 2 | I’ve got to be home soon, I think it’s a bit late now. |  |
