# Supplementary material for: Experiences of friendships of young people with first-episode psychosis: A qualitative study
Source: PLoS One. 2021 Jul 30;16(7):e0255469. doi: 10.1371/journal.pone.0255469 (PMC8323937; doi:10.1371/journal.pone.0255469)
Supplement: S1 File — (ZIP) [file pone.0255469.s001.zip › Transcripts of interviews/Participant 3.doc]

| CH | I’m interested in hearing about people in your life that you see socially, thinking about your friends at the moment. Have you got a particular friend in your life at the moment? |  |
| --- | --- | --- |
| 3 | Yes, but he’s at college. He’s the kind of guy who goes to college and all that. |  |
| CH | Ok. Has he been a friend for a long time? |  |
| 3 | Yeah, I used to go to college with him. I went college with him for about four years. |  |
| CH | Ok, so you’ve known him for a good amount of time? |  |
| 3 | Yeah. |  |
| CH | What sort of things do you like to do together? |  |
| 3 | We sort of go out like, occasionally, maybe down to Oxford Street or somewhere like that.. |  |
| CH | Do some shopping? |  |
| 3 | Yeah |  |
| CH | Can you think of a recent time that you’ve done that? |  |
| 3 | It’s been a few months you know.. |  |
| CH | And would you phone him or who would organize it? |  |
| 3 | We organize it out on the street, sort of thing. |  |
| CH | What’s the good thing about this guy, in terms of being your friend? What do you like about him? |  |
| 3 | He’s an honest guy really. |  |
| CH | So he’s somebody who says what he thinks? |  |
| 3 | Yeah. |  |
| CH | Is there anything else about him? |  |
| 3 | No, not really. |  |
| CH | What sort of role do you think you play in the friendship? What do you think it is that he likes about you? |  |
| 3 | I don’t know, just.. I’m just a normal guy really. Natural, that sort of stuff. |  |
| CH | So in the same way you like it in him that he’s quite honest and he might like the same things about you? |  |
| 3 | Yeah. |  |
| CH | Are there any other friends in your life at the moment? |  |
| 3 | No, not really. |  |
| CH | Just this guy. |  |
| 3 | Yeah. |  |
| CH | What sort of qualities do you look for in friends? What’s the important thing about friends for you? |  |
| 3 | Just for who they are really. Being honest, a natural instinct about them I’d say. |  |
| CH | Anything else? |  |
| 3 | Not really. |  |
| CH | And if this person, what’s his name? |  |
| 3 | [friend 1] |  |
| CH | [friend 1], would you consider him somebody who you could talk about anything to? Or would you keep, tend to not talk about personal stuff? |  |
| 3 | Not really, no. |  |
| CH | You wouldn’t? |  |
| 3 | No. |  |
| CH | Would he know much about the symptoms that brought you to this service? |  |
| 3 | No. |  |
| CH | You wouldn’t have talked to him about that? |  |
| 3 | No. |  |
| CH | Is there anybody you would talk to about that stuff? |  |
| 3 | My cousin, my mum and dad and my cousin, the rest of my family. |  |
| CH | So in terms of that sort of thing you’d rely more on your family? |  |
| 3 | Yeah. |  |
| CH | Is your cousin someone who you’d do social stuff with? |  |
| 3 | Yeah. |  |
| CH | What sort of things would you do? |  |
| 3 | Just go out, go to the shops like, go shopping, that sort of stuff, we’d go out for a drink or something. |  |
| CH | And your cousin, presumably you’ve known him a long time if he’s in your family? |  |
| 3 | Yeah. |  |
| CH | Is he in the same age group as you? |  |
| 3 | Yes, but he’s eight years older than me. |  |
| CH | Can you tell me about any friendships you had before you started coming to the EIS? Or before the symptoms that brought you here began? |  |
| 3 | Yeah, I had a friend when I was in high school, his name was [friend 2]. I used to hang around with him. |  |
| CH | What happened to that friendship? |  |
| 3 | We just stopped contact sort of thing. I dunno why. |  |
| CH | Did he come to college with you? |  |
| 3 | Nah he went somewhere else. |  |
| CH | Ok, so you went off and did different things after school? |  |
| 3 | Yeah. |  |
| CH | What sort of things did you like to do with him? |  |
| 3 | We used to go out and all that sort of stuff. All the same stuff really. |  |
| CH | Give me an example. |  |
| 3 | Just going out and socializing and all that sort of stuff. |  |
| CH | Ok. Do you think there have been any friendships that you’ve lost because of your illness, the symptoms that have brought you here? |  |
| 3 | No not really. |  |
| CH | So your friendships have stayed the same? |  |
| 3 | Yes |  |
| CH | You said that [friend 1] didn’t know that you’ve not been well. How have you managed to keep that from him? |  |
| 3 | I dunno. I haven’t seen him for a while, that’s the thing. He goes to college and all that. He’s probably on his holidays and all that. I see him at work and all that stuff. He works in the same place as me, [Place of employment]. |  |
| CH | So you see him there? |  |
| 3 | Yeah. |  |
| CH | Do you have any other friends at work? |  |
| 3 | Yes there’s some that I see at college as well. |  |
| CH | Did you used to go to college? |  |
| 3 | Yeah. |  |
| CH | What led to you stopping? |  |
| 3 | It was the end of the year. I was supposed to go to university but I didn’t. I didn’t get enough grades so I had to go back to college again for one more or two more years. If I can get the grades up then it’ll be university. |  |
| CH | I see. Is [friend 1] doing the same thing? |  |
| 3 | Yeah. |  |
| CH | I’m just thinking about the symptoms that brought you here and the effect on friendships and possibly college? |  |
| 3 | No, none. |  |
| CH | And it didn’t stop you working? |  |
| 3 | No. |  |
| CH | That’s good, keeping those things up. Your friends, the people at work, the people you know from college wouldn’t be aware of what you’ve been through? |  |
| 3 | No. |  |
| CH | Ok. So who would you, if you wanted to talk to someone about any of this stuff, who would it be that you would talk to? |  |
| 3 | My cousin really. |  |
| CH | Your cousin first. Your parents? |  |
| 3 | I’d sort of tell my parents. I’d tell my cousin as well. |  |
| CH | Ok. Do you think your experiences, having the symptoms and not being well, have changed the way friendships are for you? |  |
| 3 | Not really. |  |
| CH | They’re still the same? |  |
| 3 | Yeah. |  |
| CH | Has the way that you would, what you’d look for in friends changed? |  |
| 3 | Not really, the same stuff. |  |
| CH | Much the same? |  |
| 3 | Yeah. |  |
| CH | Do you do any different social activities now? Is there anything that you used to do that you don’t do now, or vice versa? |  |
| 3 | I wouldn’t like go out socializing as much like with a person. Wouldn’t as much. |  |
| CH | Why is that, do you think? |  |
| 3 | We’re just doing other things, that sort of thing. |  |
| CH | What sort of things are you not doing? |  |
| 3 | Like socializing. I’m quite busy sort of thing. |  |
| CH | So it’s more because you’re quite busy or the fact that you’ve not been well? |  |
| 3 | No, just been busy like. |  |
| CH | When you’ve been busy, what have you been doing? |  |
| 3 | Finding work, looking for work and all that sort of stuff. Finding a permanent job. |  |
| CH | Do you think friendships in your life, or the people around you have impacted on how well you’ve coped with your illness? |  |
| 3 | Yeah, definitely yeah. |  |
| CH | Who would you say has made the biggest difference? |  |
| 3 | The biggest.. |  |
| CH | Yeah, who’s helped you to cope with your illness..? |  |
| 3 | My mum and dad mainly, and my cousin as well. |  |
| CH | Do you think that having them around helped or did it not make any difference? |  |
| 3 | Yeah it helped a little bit. I don’t know really. I remember that time. I was on my own really. |  |
| CH | On your own from friends. Why was that? |  |
| 3 | I don’t know really. It was just a weird time, how I got ill. And then, I don’t know. |  |
| CH | Do you think it would have been different if you hadn’t been on your own? |  |
| 3 | Yeah could have been. |  |
| CH | Do you still feel like that now, that you’re on your own, or does that feel different now? |  |
| 3 | What do you mean by.. |  |
| CH | You said that when you got ill you felt on your own. I’m wondering if you still feel like that now? |  |
| 3 | No, not really now. |  |
| CH | What’s happened so that you don’t feel like that now? |  |
| 3 | Trying to be positive, blocking it out of my mind. |  |
| CH | Do you think there are more people around now than there were then? |  |
| 3 | Yeah |  |
| CH | And they would be your mum, your dad, your cousin, and [friend 1]? |  |
| 3 | Yeah. |  |
| CH | Do you think that anything could have helped with friendships, any extra support you could have had that would have helped keep people around, keep socializing and stuff like that? |  |
| 3 | No, that’s it really. |  |
| CH | So you didn’t feel there was anything anybody could have done to help with that stuff? |  |
| 3 | No. |  |
| CH | Anything about your friendships at the moment you’d like to change about them? |  |
| 3 | No not really. |  |
| CH | Do you think there’s anything missing in your life in terms of friendships or relationships? |  |
| 3 | No. |  |
| CH | You feel that things are as you want them? |  |
| 3 | I’d like to be more closer to [friend 1], but we sort of, we don’t see each other as much as before. |  |
| CH | So if you were to change something you’d like to be closer to a friend that you already have? |  |
| 3 | Yeah. |  |
| CH | What is it that stops you guys from doing that? |  |
| 3 | I don’t know. He’s quite busy at college and all that sort of stuff. |  |
| CH | So you find it hard to catch up with each other? |  |
| 3 | Yeah. |  |
| CH | Does anybody here ever talk to you about friends? Your care coordinator or any of the other staff? |  |
| 3 | Yeah, [care coordinator] does sometimes. |  |
| CH | What sort of things do you and she talk about? |  |
| 3 | Just about am I socializing as well as I used to and all that sort of stuff. |  |
| CH | And what would you say to that when she asks you? |  |
| 3 | I would say that I do see my friends but not as much, sort of thing. |  |
| CH | Which you feel is because everyone’s busy rather than because of your illness or what happened. Is that right? |  |
| 3 | Yeah. |  |
| CH | If life were perfect and you were completely satisfied with everything, would there be more friends or the same amount? |  |
| 3 | Same amount. |  |
| CH | Have you ever been anybody who’s had a big crowd of friends or have you tended to be a small group of closer friends? |  |
| 3 | Small group really. |  |
| CH | That’s always been the case since primary school? |  |
| 3 | Yeah. |  |
| CH | Has there been anybody who has helped you to keep up with friendships and stuff? |  |
| 3 | Not really. |  |
| CH | You’ve done it yourself. And in terms of you and [friend 1], you’re both really busy but you’re still friends. What is it about that friendship which has meant it’s kept going? |  |
| 3 | We’re sort of close. We’re on the same wavelength as each other and all that sort of stuff. |  |
| CH | So you think the same way? Are you interested in the same things? |  |
| 3 | Yeah. |  |
| CH | I think that’s everything. |  |
