# Supplementary material for: Experiences of friendships of young people with first-episode psychosis: A qualitative study
Source: PLoS One. 2021 Jul 30;16(7):e0255469. doi: 10.1371/journal.pone.0255469 (PMC8323937; doi:10.1371/journal.pone.0255469)
Supplement: S1 File — (ZIP) [file pone.0255469.s001.zip › Transcripts of interviews/Participant 4.doc]

| CH | I’m interested in hearing about people in your life who you see socially, people who you consider to be friends. Do you have anyone like that you can think of? |  |
| --- | --- | --- |
| 4 | I have a few people that I can go around and see, but when.. I think a few of my friends haven’t really changed. They’re still the same people from when.. I’d say 7 or 8 years ago, you know? I found that, the few friends that I do have, I don’t really go out and see them that often. I’m normally alone. I don’t normally go out and see my friends as much as I would like to. |  |
| CH | What sort of things would you do with those friends if you did see them? |  |
| 4 | I would perhaps have a few beers, maybe a couple of joints. Listen to some music, maybe watch some television. Have a talk about anything. I haven’t really told any of my old friends about my problems. They knew I was in hospital but they never understood why I was in hospital. They didn’t realise that most of it was through alcohol. Being at home for certain amount of time. To tell the truth I don’t get out of the house that much, but yesterday I went to [coastal town] for the whole day with this group, the EIS group. I found that I can make a few friends off this EIS as well, people that I know and meet. If you consider them as friends, then yeah, there’s some friends there. |  |
| CH | What sort of things would you look for when you’re making friends? |  |
| 4 | I would like, what I look for in friends.. Maybe just.. I like people to be really down to earth, and just be their self, be polite, be nice, and that’s about it that I look for in friends. Friends that are not full of it you know? Not arrogance. |  |
| CH | Do you think what you look for in friends is different since you had a period of being unwell? |  |
| 4 | Yeah, I think so. I look at people.. I used to look at people like maybe they’re objects, not human you know? But now I’ve got to realise that human people, what we are, we’re just.. My attitude.. I don’t know really. I’m a bit puzzled. |  |
| CH | That’s ok. You feel that there is something different? |  |
| 4 | Maybe I sort of think that the other person is special, not cos they’ve got mental problems, just that they carry themselves well, they like to be treated how they would treat someone else. You would like to be treated that way too. |  |
| CH | So you’re sort of looking for respectful? |  |
| 4 | Yeah, respect for each other. |  |
| CH | Since you’ve had that time of not being well, do you think it would be easier now to make friends with people, like from the EIS, than it is elsewhere? |  |
| 4 | Yeah. I can make friends outside, but they’re.. Some of them might have mental problems themselves, or they might have alcohol problems. They have problems. All of my friends that I have grown up with, most of them did have problems. I grew up in a tough environment or estate, so it was all boxed in and everybody knew everyone. It was like a community, but everyone had their own problems. You could see it. Alcohol problems, smoking problems, aggressive problems.. Those sorts of problems. |  |
| CH | And with that in mind, why did you decide not to tell people about being in hospital? |  |
| 4 | I didn’t want to explain. I didn’t want to explain why I was in, what I was in for.. It was all.. All my problems in my past.. I had an argument with one of my best friends when I was younger. We had a little fight and stuff, and I never really spoke to him after that. So that’s one of my best friends that I don’t really see no more. He was my best friend anyway. I do miss seeing my friends, a few of them, but then I wouldn’t.. I don’t think I would have anything to talk about. It would be just really silent and nothing to say or to talk about, nothing. I don’t want to talk about my problems because it comes across as maybe a bit selfish, talking about me all the time. That’s how I feel with friends now. |  |
| CH | Is that what would stop you phoning someone up? |  |
| 4 | Not really, no. I haven’t really thought of getting any phone numbers off my friends because I feel like I’m stuck at the moment with life. I have my flat but I haven’t moved in there yet, like actually moved into the apartment. Once I start doing that I’m sure I can start getting out a bit more, making new friends, socialising a bit more. |  |
| CH | So you think that once you get into your new place it will seem easier? |  |
| 4 | I think it will, yeah. I think I’ll become more independent and more, I think I’ll feel more happier you know, in my self, with my self. I don’t need to say I live at home with my parents - I have my own apartment, would you like to come round and watch a DVD or.. In the past all that I really used to do with my friends was get high or get stoned, one of the two. I wouldn’t do nothing else with them. |  |
| CH | Being at home with your mum and dad is limiting what you can do with friends? |  |
| 4 | That’s right, yeah. |  |
| CH | Do you think there’s any friends that you lost touch with or have fallen out with because you weren’t well? |  |
| 4 | Maybe just one. |  |
| CH | What happened there? |  |
| 4 | He was judging my weight, calling me fat, saying I looked pregnant when I was ill. And it really.. It made me paranoid around other people in case they judged me how I look - my size, my weight.. I don’t like him no more. I wouldn’t.. I haven’t got no anger towards him for what he said, but I really found it was out of order. We grew up together, he was a good friend and he was judging my weight. I’ve never done that to no other friends. Never judged their weight or their personality or the way they dress or the way they look. I find it a bit heartbreaking you know? I didn’t like it, so one friend I don’t really see. |  |
| CH | It sounds like that was your decision not to see him anymore, because you felt judged? |  |
| 4 | Yeah that’s right. He was disrespectful. |  |
| CH | The friends that have been around, what do you think it is about them that has meant your friendships have carried on? |  |
| 4 | Because my past, my background, having a good teenage years, growing up, had a good life you know, enjoying life, partying, getting out everyday, hanging out maybe 5 hours a day with a group of friends.. |  |
| CH | A shared history? |  |
| 4 | Yeah, we all have that close bond. Like you would with a brother or sister, that sort of bond. That’s why I would find it difficult to talk about any of my own problems to them – I don’t they would understand what I went through. |  |
| CH | In your friendships that are going still, that you see from time to time, what sort of role do you think you play? |  |
| 4 | I think I play like.. I feel like it’s my friends used to call me big Bal. I used to like it you know. I felt, not powerful.. Powerful is not the word. I felt strong. I felt that I played a strong role. Physically, mentally, I’m there to listen to them, to understand them. What I’ve realised by making a few new friends off of this EIS group is I ask them for help or they ask me about their problems; I can talk to them more easier than old friends about my problems. They’re on the same sort of medication that I’m on. I can understand them, they can understand me. I find it easier to talk to someone who has mental problems. Mental health problems easier than talking to someone who hasn’t had them or hasn’t, won’t get them. |  |
| CH | So before you had quite a strong role, would you still play that same sort of role now if you saw those mates? |  |
| 4 | Maybe, yeah, I would. |  |
| CH | And I might have got this wrong, but it sounds as if you talk to them about your problems that would make it seem less like you were strong? |  |
| 4 | Yeah, that’s right. It would make me feel really different. |  |
| CH | We’ll come to friends from here, from the group, in a minute. But these friends you’ve known for a long time, what sort of thing do you like from them? |  |
| 4 | They value my friendship, my presence.. |  |
| CH | What do you like about them? |  |
| 4 | They make me feel good about myself. That’s why when one of them said I’d put on weight and I looked pregnant it did hurt me a little bit, because I’ve never been judged like that. I thought surely he can’t be talking about me but he was. I feel like if I was to see him again, the same guy who disrespected me, I wouldn’t even look at him, no eye contact. I wouldn’t bother. I couldn’t be bothered to talk to him about anything because he was disrespectful. With some friends that I had, they’re really close, like family, known them for years and years and grown up together. What I do miss sometimes is going to see some of these old friends and maybe just driving about together, maybe going out on the town for the night you know? Enjoy [city] a little bit more. I’ve noticed now I’m older that [city] can be a really lonely place if you’re by yourself. That’s what I’ve found out as I’ve got older. |  |
| CH | What do you think stops you from going out on the town and doing that stuff? |  |
| 4 | The way I look. I look in the mirror sometimes and I feel overweight. One day I could look in the mirror and I might look good and feel good. I suppose it happens with everyone really. You can look good one day and bad the next day but I suppose I feel like, if I was with my friends all the time it would bring out.. It could bring out the animal in me? If I’m with a crowd of my friends and that I feel I might have a big ego, have a few friends. |  |
| CH | And that stops you from wanting to do stuff? |  |
| 4 | Yeah. Mostly not doing things with them because I’m not really happy with my physical appearance. I find that, if I did liked a lady or a girl and she would look, and if I did like her and she wouldn’t like me because of my size, I feel like I get problems like that sometimes. |  |
| CH | So you’re not feeling very confident about yourself. Do you think that’s since you were unwell or have you always felt like that? |  |
| 4 | No, since I was unwell I think. When I was ill I was really slow. When I came out of the hospital the first thing I done was get some drink, which I shouldn’t have done, and went and sat up in my friends house, one of my best friends called [friend 1]. And after that I haven’t really seen them that much because I don’t want to go up there and start drinking around them again. We got violent towards each other after a few drinks. We wanted to have a fight with each other and he thought I was flirting with his girlfriend or whatever but I wasn’t, and I hold a grudge - if you want to call it that - with my friend. I didn’t want to fall out with him; I thought he was a genuine guy. I thought he was nice and just good company. I enjoyed his company, he liked me company. The same friend whose company that you enjoyed, it turned to aggressiveness and anger, we don’t enjoy each others company anymore, so we didn’t really… I miss looking, going to see friends, I really do miss it, then I think but I can make new friends, as I’m older now you know? |  |
| CH | It sounds like since you were unwell, some of your old friends wouldn’t have that helpful to keep you well? |  |
| 4 | Yeah, that’s exactly how I felt as well. |  |
| CH | You’ve talked about some violence but also a lot of drinking, which you were perhaps trying to avoid? |  |
| 4 | Yeah that’s right. I didn’t like to always go out and drink and smoke and get high all the time with my friend. It just got a bit boring after a while, doing the same thing over and over again, that’s what it seemed like, going round and round and round and carrying on and on and on. |  |
| CH | The friends, or the people that you’ve come to meet through the EIS, what sort of role do you think you play with those friends? |  |
| 4 | Sometimes I feel like, I’m the dad role, the role model, someone to look up to. Someone to see that everybody has their problems but not everybody’s problems are the same, so if I was to talk to someone about their problems I’d be saying to them about my problems. I think there’s more of a bond than I did have with my old friends, or a different sort of bond than I had with my friends than I do with the people from the EIS. I find that the people at the EIS, I feel like I could be the dad model because I’m the oldest if you want to call it that. They’re all a bit younger than me, early twenties. I’m almost 30 now. More responsibility on my shoulders. Not to look after them, just to lend a hand, you know? If they need anything or how do you feel.. I feel like a doctor or like a relationship a doctor has with his patient. I feel like that with these people from the EIS. I feel like that sort of ‘bondship’. |  |
| CH | Protective? |  |
| 4 | Yeah, protective and stuff, that sort of thing. I feel like I protect them, my friends. I’ve always had that sort of side to me. |  |
| CH | Is that a role you enjoy? |  |
| 4 | I do in a way, I really do. I know that - without talking about violence - I know that I can look after myself and handle myself well. If anyone wanted to hurt me or harm me I could really look after myself, but some of these people from the EIS group can’t do that, can’t look after themselves, so I find that if I was to walk down the road with them, I would be like a bodyguard. I feel that role sometimes. Only with friends from this EIS though. |  |
| CH | So they might seem a bit vulnerable? |  |
| 4 | Yeah, I’d protect them. |  |
| CH | It sounds like, to not the same extent perhaps, but to your old friends you were the strong one, and that’s important to you? |  |
| 4 | Yeah, my character, full of life, enjoying everything, that’s how I used to be with my old friends. There’s nothing much that I miss about them, except maybe their company, you know? Not what they’ve got to say or what they’ve got to talk about, just their company in general, from all those years that we grew up together.. See what they are now. I don’t see them, I very rarely see them. That’s what I miss, just their company, or maybe just to see them, but then I wouldn’t like to have any comments again because that was really downgrading and I didn’t like it. It made me walk down the road and think everybody was judging me or looking at me and thinking I’m over weight or getting big and fat. |  |
| CH | Is there anything that could happen that would mean you would want to get back and see them or be out and about? |  |
| 4 | If I was to join the gym, which I have done, but I don’t really go. I should really its just round the corner from where I live, but I should use the gym and I would start feeling better in myself, and then if I do see them again, the guy who said that I put on weight would say that I’ve lost weight and then I would feel a bit more better, you know? I’m still up to here in my brain with him telling me that I look pregnant and stuff. It’s not right to talk to people like that, not in this day and age. |  |
| CH | So if you raised your confidence a bit.. |  |
| 4 | Yeah, getting out and seeing people. |  |
| CH | Is there anything anyone could do to help you with that do you think? |  |
| 4 | Myself really, just myself. A bit of willpower. I’m really surprised how many people do suffer from mental problems. In [local area], or [local area] especially, or [city] in general I suppose, who suffer from mental health problems. I would feel better if I could slim up a little bit, lose a few pounds, and just.. I think I don’t want to turn into a big muscle man, I just want to be a bit more slimmer so my clothes fit better, hang better. I carry my weight a bit more instead of thinking old friends disrespecting me. I would feel more better if I could start using a gym. Even if I went for one hour a day, one and a half hours. One hour would do I suppose, just go for one hour a day to tone up my body and I suppose in a few months time the first thing I would do, I would knock on a few old friends doors and show them my new look, my new body, that’s what I would want to do. I don’t think I could do that now. I think they might still be judging me or, you know.. A few of my friends used to say that I had a crazy side to me when I was growing up. I would never harm no one, maybe crazy but nothing to hurt anybody. |  |
| CH | Is there anybody, apart from in the EIS group, that you have talked about your mental health problems with? |  |
| 4 | Not really, no. |  |
| CH | Do you talk with your family about that stuff? |  |
| 4 | No, I don’t. I don’t really talk to them about my problems and stuff. I’ve done a path with [support worker], of my past, my future and present and its got pictures underneath of all my problems and stuff, and she’s really good. I don’t think my parents are interested, you know? My dad has diabetes, he’s been diagnosed with diabetes and I suppose they’re looking after theirselves a bit more, they’re getting old now. Fifties, not old but older than what I am. |  |
| CH | So you use the professional people here to talk to instead? |  |
| 4 | Yeah, but when I want to talk, I would talk about my problems but then I think anybody who drinks quite a lot of alcohol or smokes a lot of weed in their day its going to affect their body, how they talk to people, how they feel. They get really tired or whatever and they don’t really want to talk about their problems. It just numbs your pain, you know? Alcohol and smoking, just get rid of everything. Just keep high and stoned and don’t care about anything. That’s what I do sometimes anyway. I know I shouldn’t. I should not be really drinking. I don’t drink on top of my medication but it don’t.. I don’t think the two mix together, alcohol with my medication. All the problems I had in the past when the police used to come round my house and get me of the streets drinking a can of beer and bring me home or whatever, it got, after a while, I think, they always used to see me on my own, so they thought that I didn’t have a bunch of friends around me, so that might be why they stepped in and helped me and put me in hospital because they seen that no one was around to help me and they saw that I was in a right state mentally, my head was all over the place, felt really bad, depressed. Didn’t want to.. They looked after me like they were friends at the time. They were friends to me, the police, helping me seeing that I had problems. The other people, my old friends, they didn’t really used to ask why I was in hospital or what I was in there for. It don’t really interest them. I suppose if I was to see a few of my old friends again I wouldn’t mention my mental health problems. I would say I was in hospital and that was it. I wouldn’t mention that - how I used to feel and what I used to do to make myself feel better. |  |
| CH | The time that you were in hospital, your friends weren’t around were they? Do you think things could have been different for you if they were? Would it have changed you getting better? |  |
| 4 | I felt like, at the end of my hospital, when I was there for 7 weeks, say about the 5^th^ week, I really wanted to go and see a friend. I needed to see a friend. But no friends were around at that time. The only friends that I could make were the ones in the hospital. The ones, the other patients or whatever that were on the ward with me for the same sort of reasons. |  |
| CH | What sort of people were they? Did you stay in contact? |  |
| 4 | No, I never stayed in touch with them, but when I do see them and I have seen a few of them on the streets they’re really happy and cheery, say its nice to see me, and happy that I’m ok. Maybe have a drink with one of them, go home after, or talk to them, how are my problems now, am I still on medication and stuff like that. They give me advice, and I appreciate that advice. They understand what mental problems are about, they understand them I suppose. When I was up at the hospital I would listen to them all instead of just hanging about not listening. I really listened and understand what they was talking about, and telling me that everything will be ok. It seemed like when I was first in hospital, it seemed like one big dream, and that’s when I really needed a friend but no one was around. No friends of mine saw me when I was in hospital. It would have helped me I think. |  |
| CH | Which way would it have helped you? |  |
| 4 | I think it would have helped me mentally, with my mind, just not thinking about problems, thinking about the bad all the time - negative, negative, negative. Instead of thinking about the positive things in life, the good things, the simple things in life. That’s the only thing that I’m missing at the moment. I would like to get everything like that back again, like having a girlfriend, got my own flat, get a car, get a job. Just a simple life, that’s all I’m missing at the moment. Once I get that I think I’ll feel more better in myself. I’ll keep it, I wouldn’t feel like everyone’s watching me or judging me or telling me I’m overweight or whatever. Simple, it’s just around the corner you know. I think my life will be a lot more better. I’m at a point in my life now where I don’t really need no friends. I feel, how can I explain it? I feel that I don’t need support from any of my friends. I feel that I am my own adult now. Fully grown adult, a young man if you want to say that. I’m 28. I’m going to be.. I don’t want to get old before my age, I want these next two years to be most probably the best years for me for getting back, getting off medication, mixing around people. That’s the only thing that used to get me down, being around all the time, no one to talk to, no friends, just being alone. I couldn’t understand my self so I couldn’t be around other people if I couldn’t understand myself. So I was not talking, most probably one of my friends would keep going ‘what’s the matter, what’s the matter’ and that. I suppose then it would get a bit annoying, and then see you later, and then won’t see them for another 2 or 3 months. My best friend I haven’t seen for about 9 months. It would be good to see his face again but I don’t really have anything to talk to him about. That’s why I was talking about joining the gym because I’d feel a lot more better, I’d feel more toned up, ‘how you doing’. Even a job, having a job. When I was growing up I had quite a lot of work. I was earning up to £100/week at 16, so that was really good. Saving money, going out all the time, having some drinks, enjoying life, enjoying having a girlfriend, enjoying life, just normal in general. That’s the only thing I miss now. So when it comes to friends, friends are maybe second or third or fourth or fifth on my list. First on my list is trying to get a job, second is getting my flat sorted out, third is start being a bit more sociable, cos I know that when I was at Brighton yesterday I thought wow look at all these people, but none of them were looking at me like, I felt at ease with myself. No one gave me bad eye contact, just normal. That’s what I really miss; I miss getting out. |  |
| CH | It sounds like its important to be independent, to know that you don’t need those people but to have them around, socialising. It’s a want rather than a need? |  |
| 4 | Yeah. |  |
| CH | So in terms of the sort of stuff you would like to do to socialise now, it sounds like that is different to the sort of things you would have chosen to do before? |  |
| 4 | Yeah, a lot of them are different, for the better though I suppose. There’s nothing really good about my old friends. It’s just that we were all from the same area, just it was really nice. I enjoyed it all, growing up and just, not worrying about stuff. When my mental problems started happening, I stopped being sociable with my friends. That’s when I used to lock myself away and used to have bad dreams and bad nightmares, and my friends would be in some of my dreams and nightmares. They are, still is, which is pretty strange. |  |
| CH | If life was good, you talked about having a job, a flat, some money coming in and stuff. What would your friendships be like in an ideal, this perfect situation? |  |
| 4 | Seeing them twice a week, going to a bar, have a couple of beers. Maybe bring them back to my flat, watch a DVD, then say ‘see ya later’, go to bed, have a rest. Or just do the normal thing which would be the best – get a mobile phone, and if you don’t want to talk to them, switch your mobile off. Simple really. That’s still the best way to have contact with people – through a mobile phone. There’s nothing easier or simpler than using a mobile to keep in contact with people. That’s what I would do, I would get down more phone numbers, more people that I meet or see in the bar. Speak to them, mix with them. I would like to meet new people. Not people who have mental health problems or old friends, just people in general. Reality people, like I would call it. People who would be in reality. People who are just real to theirselves and real to life. Have the respect for life, just get along. That’s all I miss at this moment, which could be better, considering my old problems in the past. Not mixing with anyone at all. Even if I might be indoors at home I’m still mixing with my parents. There are people there in the house so I’m not completely alone. |  |
| CH | Do you not use a mobile at the moment? |  |
| 4 | I do but I don’t have no credit. I’ve got to buy some credit first. I’ve got a mobile back home, yeah. I’ll get some numbers down. I got some from the EIS group. I got some numbers down to talk to and stay in touch with. |  |
| CH | Is there anything you think the service here could do to help you with any of this – friendships and socialising? |  |
| 4 | Not really. It’s myself I think. I’ve had all this helping me, going badminton, museums, cinemas, playing pool, going bowling, doing everything, I’ve done all that and I’ve really enjoyed it, but I’ve realised that now’s the time to get on with my life. My mental health problems are getting better - with each day that goes I feel a lot more better. I really do, I’m not just talking and saying it for the sake of it. I do feel better in myself. I can be around people now without thinking that they’re thinking look at that, you know? I think it’s really bad to judge someone who’s had mental problems. I think you should just leave them and let them get on with their life. And if they are having problems, come and talk to the people at the EIS. If you’re having problems with your family, talk to them. I’ve done it before; I’ve talked to the people here at the EIS about loads of things, all my problems. Whenever I have talked to them about my problems, they’re good listeners. They know how to listen. I think it’s really arrogant not to listen, use your ears, you know, that’s what they’re there for. |  |
| CH | Is there anything you want to add that I haven’t asked you yet about friendships? |  |
| 4 | No, not really. We’ve covered a lot, done really well. |  |
