# Supplementary material for: Experiences of friendships of young people with first-episode psychosis: A qualitative study
Source: PLoS One. 2021 Jul 30;16(7):e0255469. doi: 10.1371/journal.pone.0255469 (PMC8323937; doi:10.1371/journal.pone.0255469)
Supplement: S1 File — (ZIP) [file pone.0255469.s001.zip › Transcripts of interviews/Participant 5.doc]

| CH | So what we’re interested in hearing about is people you see socially. Can you think of any particular friends that you have in your life at the moment? |  |
| --- | --- | --- |
| 5 | Yeah. |  |
| CH | Can you tell me about somebody? |  |
| 5 | Well just about them or..? |  |
| CH | Who have you got in mind? Are you somebody with a lot of friends? |  |
| 5 | Yeah I’ve got a fair amount of friends. I wouldn’t say a lot. I tend to have friends from university and also friends I have made since I’ve moved to [City], which was about 10 years or so ago. |  |
| CH | Who would you say you see the most often? |  |
| 5 | I kind of see somebody once or twice a week and it’ll always be different ones, but then sometimes I don’t and like I’ve got friends I see once a month or something or once every two months. Most of them now have children and it’s a bit more difficult to see them regularly so I can’t see them. You know, in the evenings they’re putting the baby to sleep or whatever and they are too tied as to when you can see them, you know? |  |
| CH | Okay, have you seen any friends in the past week? |  |
| 5 | In the past week? No I haven’t actually, mainly because I’ve been busy at work. Yeah, and also I’ve had lots of weddings going on. I saw friends last weekend and the weekend before as well. It was another wedding so its been very busy so I have found myself kind of working and then going home and eating food and then going to bed. I am seeing friends tonight actually, friends who I know from work. We’re going wine-tasting on [local street].  I’ve known them since starting work which must have been about five years ago? They just got back to the UK because they were working in [African country] and they got back recently so it’s been good seeing them more than I would have done because of them being away. |  |
| CH | What sort of things do you do with these friends then? Wine-tasting is unusual. What sort of stuff do you do normally? |  |
| 5 | I usually meet them for lunch because we work so it’s kind of easier to meet for lunch but in the past we used to go to the pub. Yeah, with these two its been mainly lunch and going to the pub, and then wine-tasting I do once a month or so. There’s a place I used to go to in [local area]. Its just really nice - you meet and its free, quite a nice place. |  |
| CH | Is the sort of things you do with those friends different to what you do with your university friends or new friends? |  |
| 5 | I think so. I think my friends from university, I tend to go round to them and their house because they’ve now got babies and its difficult for them to travel so I go round to their place, have dinner, just chat, sit around maybe go out in the evening for a walk before dinner or something but yeah if it’s the weekend it might be a picnic in the park or something. Yeah, so I think it’s a bit different. |  |
| CH | People play different roles in friendships - what sort of role do you think you play in friendships generally? |  |
| 5 | I don’t know really. I think.. I don’t know. Some of my friends come to me for advice. When they’ve something they just want to talk about they come to me and talk to me. I think work friends is more about having fun or its gossiping about people from work or people we know mutually. I don’t know really in terms of what role I have. |  |
| CH | It varies perhaps. |  |
| 5 | Yeah. |  |
| CH | Okay and instead of what you do for them, what are the important bits for you in those people? |  |
| 5 | Yeah I think people that.. I can talk to them about things and also just have a laugh and be a bit silly and not really have to worry about what I say or how I say it or what I do. Friends like [friend 1] - he’s just really funny and makes jokes and laughs most of the time. |  |
| CH | Okay, so quite light-hearted? |  |
| 5 | Yeah, but then on the other hand he can be serious if he needs to be and talk about serious things if you’re not feeling right or needing help. |  |
| CH | Do you think that the things that you look for in friends now is different to what you looked for perhaps before you had symptoms that bought you to the service? |  |
| 5 | A little bit, but not really. I mean the friends that I have now are pretty much the same as the ones I had before I had my episode. I think there’s one friend who I think the relationship changed a little bit because of the way she reacted when she first heard about what happened, but she’s still my friend. I think it was just difficult communicating with her at the time and I.. Recently, you know, I still think things were better but she’s only talked a bit more to me lately than before. A bit more openly, but I think its just affected her I suppose, in that she didn’t realise what had happened. And yeah, I mean in terms of what I look for in a friend nothing’s changed that much but I am, I think, a bit more.. I guess more aware about mental health issues so things that people might say now - whereas before I ignored them - I might challenge them and its probably made my friendship stronger because people are like ‘oh right’ and kind of understand, and I think maybe if its not my friend it might make me form an opinion about them in a different way you know? If I hear them talking about crazies or crazy people and that kind of thing and it might make me more reluctant to want to be friends with them. I try to challenge it and just go watch me.. |  |
| CH | With the friends rather than those people that you don’t know, would you talk to them quite openly about what had happened to you? |  |
| 5 | Yes. I try to. I mean I try to be as open as I can with people at work. Yeah, but with my friends, I think all my friends know what happened. They might not know every detail but they know I was in hospital. I think it helps me because they know I was on medication and I had a little celebration when I stopped taking medication and things like that. |  |
| CH | Was there anybody during that time when you were unwell that was particularly around? |  |
| 5 | Yeah. Especially after. When I was in hospital there weren’t really friends it was more my family and my boyfriend who, my fiancé I should say – sorry – like when I was in hospital it was mainly them who were around. I’ve got one friend from work who is now in [abroad]. She was around a little bit at the time. She knew what happened, she was one of the first people I phoned and I was in a proper state just before I went into hospital and yeah, I mean I don’t see her because she’s in [abroad] but I speak to her regularly now. |  |
| CH | And do you think having her at the end of the phone as it were, made a difference to how the episode progressed or do you think it was the same as it would have been? It’s an impossible question I know, but in your opinion? |  |
| 5 | I’m not sure what I think. I think she did help and I think she was the one person that I trusted while I was having paranoid thoughts about everybody else. There weren’t that many paranoid thoughts but I don’t remember any thoughts about her. Whereas people from work I had paranoia about them so speaking to her helped and she tried to calm me down and tried to understand what was going on, but it was so confusing that I don’t think she really knew. Yeah, so I think it helped because she’s got, she used to be a solicitors clerk in a firm that specialised in mental health cases so incredibly helpful afterwards. She kind of understood a little bit of what I’d been through. |  |
| CH | Okay. So as you say, perhaps her and your family whilst you were in hospital, and when you came out of hospital did friends play much of a part then? |  |
| 5 | Yeah. I mean, I’ve got one friend she’s coming round for dinner tomorrow actually, she was pregnant at the time and an ex flatmate. Because she was pregnant and not working when I came out of hospital I spent quite a lot of time with her and she only lives down the round from me so we used to do things in the day, go swimming and go to exhibitions and just chat, hang out and listen to music, bake, so yeah I’d see her twice a week, two- three times a week and then it gradually became less because I got to go back to work so… |  |
| CH | What do you think it was about that time that was helpful? |  |
| 5 | Just being with someone. Being on my own was really hard. My boyfriend works during the day so he was out and at the beginning my mum stayed.. Sorry its getting a bit hard for me to… [ptp tearful] |  |
| CH | Its ok, it’s remembering a difficult time isn’t it? |  |
| 5 | Yeah, my mum, sorry.. |  |
| CH | It s ok, that’s ok. I’m sorry I’m not sure we’ve got any tissues in here. |  |
| 5 | I’ve probably got some. My mum, she’s retired, so she’s spent quite a lot of time with me when I first came out of hospital, but then I kind of asked her to just go home really. I wanted things to kind of go back to normal but then when I was on my own it was really hard so it was good to have someone who wasn’t my mum and who wasn’t my sister and who wasn’t related to me and was just my friend. |  |
| CH | Why do you think that was that it was better - for someone to have a bit more distance - do you think? |  |
| 5 | I think you always feel like - not so much now - but my parents and my sister were always trying to tell me what to do and I think, you know, when I came out of hospital it was a lot of very well meaning advice. You know, ‘you should do this, you should do that, you should think about talking to this person, you should think about doing that’ and my friend was like ‘do what you want!’ |  |
| CH | A bit of company but without crowding you with advice? |  |
| 5 | Yeah. |  |
| CH | Okay. Was there anybody when you were in hospital or afterwards - I think you mentioned one friend actually already - that didn’t understand perhaps or found it difficult to be around to talk about things? What stage did that kick in? |  |
| 5 | Well I think that I went into hospital really suddenly and then I was in the hospital for the full 28 day section, although at the end I was coming home on day leave and things like that, overnight leave and at the end I was practically at home. But the first time that I talked to this friend after I came out of hospital, I hadn’t seen her for a good month and it had been my birthday just after I went into hospital and she said to me ‘oh how are things’ and I said ‘oh its okay I’m getting used to being back at home and not having to go to hospital’ and she was like ‘oh yeah’ and I said ‘I have been in hospital and for quite some time which is why I haven’t returned any of your phone calls’ and she said ‘oh I thought there was something wrong when you didn’t return my phone calls’, so it really hurt to hear that because I was like well, she seemed to be more worried about the fact that I hadn’t returned her phone calls not about me. Then when I did see her and tried to talk to her about it she didn’t really.. I could tell she wasn’t comfortable. She didn’t want to talk about it and she didn’t ask me any questions, she kind of just let me talk and I kind of felt she didn’t really want to listen and so I just left it at that and then I just felt a bit kind of like doesn’t she care? Now I don’t think it is that she doesn’t care, she just didn’t know what to say, or it was too difficult to talk about. So that was kind of the one. Everyone else had asked questions had a laugh about it, and things that happened were funny in some ways, in retrospect. You know a lot of my friends were there for my boyfriend as well. I mean, they were phoning him but she didn’t and maybe she didn’t feel comfortable doing that. You know, she knows him as well - I’ve been with him 10 years so you know all my friends know him well. Yeah, I think she.. I think it was difficult for her but at the same time it made me angry because if it’s difficult for you to talk about it you know what’s it like for me? |  |
| CH | Thinking back then you had a huge, or a good number of friends who were there or were supportive, wanted to talk about it and supporting your partner as well, and then this one friend who didn’t. I am wondering if there is some characteristic or quality in your friends that were around that helped them to be there, for the friendship to survive if you like? Do you think there’s anything about them particularly or about the friendship? |  |
| 5 | I think they’re much more open and I think.. Yeah, they are much more open and about things in general. If I think about the one friend, since she got married and has now got a little boy, when she is with her husband its very difficult to talk to her about anything. On her own it’s much easier but even then its sometimes like.. You know, it’s very difficult to talk to her and I’m asking all the questions. I just kind of think well maybe she doesn’t want to tell me and that’s fine you know, to a certain extent. For example, we went for dinner with her and her husband and there was me and my boyfriend, and I hadn’t seen her for a couple of weeks but suddenly I found out that she kind of set up this whole website and its been running for like the past 9 months or something, and its kind of her website that she runs and she gets other people to make comments on it and things like that and I think its been running for 9 months you know? That means that she’s done research, that she’s done this that and the other and she just didn’t say anything to me about it. Whereas all my other friends even if I don’t see them that often or talk to them, you know, every day or whatever I feel that I know most of the things that go on in their lives you know? Its not in detail but I have some idea what’s going on in their lives. |  |
| CH | Do you think that friend has always been a little bit more detached or is that since you were unwell? |  |
| 5 | No. I think since she got married. |  |
| CH | Ok. |  |
| 5 | It was before I went into hospital, a couple of years before I went into hospital, so it’s been six or seven years she’s been married. |  |
| CH | So you’d relate it more to that than when you were unwell? |  |
| 5 | Yeah. |  |
| CH | Do you think that the way that she was when you came out of hospital had any impact on your route to recovery? |  |
| 5 | Not really. She was one, compared to a lot. |  |
| CH | Okay. Can you tell me if there are any friends you’ve made since you were unwell? More recent friends? |  |
| 5 | People at work, people at work. I mean even though they’re not friends as such, we socialise out of work, drinks after work or lunch, that kind of thing. I see them every day and you kind of feel like they’re friends even if you might not see them on a social level. |  |
| CH | That’s interesting. You said they were from work but they weren’t really friends and I wondered what would make them move over to the friendship box? |  |
| 5 | Yeah I guess it’s if I start seeing them socially outside of work, at the weekend. The people that I see every day at work, I see them and talk about the weekend or things with them, have a ten minute chat in the corridor or whatever but you know, I wouldn’t call them friends as such apart from a couple of people who I would call friends. My landlady actually works in the same department as me now and she, I would say she is a good friend even though I live in her house so it’s a slightly different relationship. |  |
| CH | Is there anything about that sort of acquaintance/friendship that’s different from people you’ve known for a long time? |  |
| 5 | Yeah, I guess people at work don’t know me as well and to a certain extent I think maybe you put on a bit of a façade at work, in terms of how much you want people to know and you know, you want to be professional and all that kind of thing. I guess there are some things that you might not ever talk about in detail with some of the people at work but whereas with my friends you know, you go into detail and things like that. Again, with people at work I try and be open about what happened in terms of my illness and kind of on a need to know basis, because my line manager she knows that I need to leave early for medical appointments, well at least once a month, like kind of early to come here but then also she knows that I’ve got an occupational health appointment sometimes with the occupational health at work to check I’m coping with work and then there are days that I was off sick, and when I first came back to work my performance wasn’t very good you know? She knows the history. I don’t think she knows exactly why I wasn’t at work but she’s never asked and it’s never been an issue. |  |
| CH | So when you were unwell you were off sick; did you go back to the same job afterwards? |  |
| 5 | I did but then I changed jobs shortly after because the job that I was doing before was just far too stressful and I think that was part of the reason why I got unwell, so I moved into a different department. Well, it’s the same kind of group but it’s a different team and its completely different kind of work and a lot of the people kind of move on quite regularly because you have two years in a job then you move to another job so a lot of the people who were there at the time I went sick are no longer there, well they’re there you know but about to leave, that kind of thing. |  |
| CH | In that first sort of period of going back to work and being around those people again do you think the way you were around them was different to how it was before? |  |
| 5 | Yeah, I think when I first went back to work I wasn’t as communicative. I found it very difficult to talk to people and engage in conversation. It wasn’t that I wouldn’t talk to people at all, I would say hello and kind of make small talk but then I found that I couldn’t engage in conversation in a way that I could before or that I can now, in terms of making the links. It was very much they talked and I couldn’t think of how to progress the conversation, the conversation kind of stagnated whereas now it’s proper dialogue. |  |
| CH | Sort of back to normal conversation? And how do you think you’ve got from there to here? What’s been the important bit for you do you think? |  |
| 5 | So many things. I mean accepting what happened and trying to understand what happened and why it happened, and how to avoid it happening again, and learning more about myself in terms of my limits and you know what to do if you think its too much, and even just recognising it is too much and then just perseverance really, getting up in the morning and putting yourself in that situation. The days that are bad, going ‘ok, it’s a bad day, just get on with it’; scream and shout and cry and it’ll be better tomorrow. |  |
| CH | A lot of people say that actually; that afterwards its difficult to be around people socially but kind of keep putting themselves in that situation makes it start to feel normal again, starts to kind of be ok, almost like getting back on the horse..  Is there anything about your friendships and the people in your social networks at the moment that you’d like to change? |  |
| 5 | I kind of wish that I could bring them altogether because I have friends from university, friends from work, friends of friends, friends of my boyfriend and I kind of see them individually and I might see them with their group of friends or that kind of thing. But I kind of would quite like them to be all friends together in just one big group. And for birthdays and things like that I think ‘are they all getting along’ because they don’t really know each other apart from through me and things, so yeah I guess if there was one thing I could change it would be that they had all been friends as long as I’ve been friends with them. |  |
| CH | Is there anything that you think is missing or that you would need from friends that’s not always there or that’s difficult to come by? |  |
| 5 | Sometimes I think.. You know, I think.. I feel especially now they’ve all got children and their priorities have changed, I kind of think that maybe you need to come to me for once and why do I have to travel just because you’ve got a baby. So maybe that’s kind of for me to accept that their priorities have changed but I don’t know. |  |
| CH | You would like your needs to be put equal to their needs? |  |
| 5 | Yes. |  |
| CH | I am wondering if there’s anything the service could have done or if the service here did anything to help you socially; did you come to the groups or anything like that? |  |
| 5 | Yeah before I went back to work I came bowling once.. Was that it? There was also a funny thing that they organised. I wasn’t quite sure why I was there, it was like a client meeting or a service user meeting or something where you were supposed to give in your views about the service and somebody who works here came along and there were crisps and fizzy drinks and apples and they were asking us what we thought about the service and it was quite a long time ago, and I was under the impression that there were going to be lots of other people using the service there but there was just me and another guy who when I talked to him afterwards said that it was all lies, that he didn’t have psychosis and that it was all lies. There was another guy who came in and stole some crisps and walked out again. I only came to the bowling once; all the other times it clashed with something else I had on. Yeah it was nice, the bowling; it was good. |  |
| CH | I was wondering if there was anything in particular you got by coming here and meeting people through the bowling that you wouldn’t get from your friends in your normal situation? |  |
| 5 | Yeah. I think it was difficult because I only came once. I did kind of want to talk about the experience that I’ve had to somebody who might understand what it was all about. When I first came out of hospital I spent a lot of time surfing the internet and mental health forums looking about what other people where posting about their experiences. I spent a lot of time doing that when I should have been working and when I first went back to work as well. There was something about hearing about other people’s experiences, even if it was just on a computer through forums and things like that and finding out about all these organisations that were out there to help, and various leaflets and things they’d published to kind of help even if I knew the information it was good to get some reinforcement. I guess the bowling was kind of, there wasn’t really a chance to mingle even though we were all bowling and the group.. There were mainly men in the group. I was the only woman there. It wasn’t bad but it made the dynamics kind of very focused on the bowling and winning the bowling and then afterwards we had a bit of a chat, again it was just small talk. It wasn’t sharing experiences and things. |  |
| CH | Would this have been something you would have been more interested in? |  |
| 5 | Yeah I think it would have been good and actually my friend’s sister, she had a psychotic episode a few years ago, about seven years ago now and I only found out through my friend when she found out about me she was like ‘oh my sister a couple of years ago went through this’ and I was in email contact with her for a while because my friend was like ‘you should email her’. So I was being in email contact with her for a while which really I kind of enjoyed and I liked talking to her through email. But I guess if I’d had more of that it might have filled a need in me, I wouldn’t have surfed the internet at that time. |  |
| CH | Has anybody here talked to you about friendships or quizzed you about your social support or anything? |  |
| 5 | When I come to see [care coordinator] she always asks ‘how are things going?’ and ‘how’s your family?’. She doesn’t really ask about friends as such but she always kind of asks what am I up to, and I go to yoga as well and she always asks how’s the yoga going, are you going regularly but nothing ever in detail and its always kind of quite general, and as long as I’m healthy I think she’s quite happy. |  |
| CH | And how about you? Would you like to be talking more about that or are you quite happy that that’s meeting your need? |  |
| 5 | Yeah, I think its okay. I now think sometimes I come here, and I wonder why I come here because I’m like ‘oh I’m just going to have my small talk’ with [care coordinator] but then you know, I think it’s important to check in. But then I’m well, I’m working fulltime and at the beginning coming here because, I guess at the beginning when I was first referred here, coming here twice a week and that gave me structure because when I wasn’t working it was very tempting just to lie around the house in my duvet and so it kind of helped and gave me structure. |  |
| CH | Is there anything else about friendships or social stuff that you think is important over the last couple of years? |  |
| 5 | I think my friends, my friends who are really aware of mental health issues, like I’ve got a friend who used to work for MIND, and I think the people who are more aware about things it was much easier to go to them for help if I needed to talk about something more. If I just wasn’t feeling right it was much easier to go to them and say ‘oh can you come and help me’ or ‘can you spend the day with me’ that sort of thing, but yeah, so at the same time the friends who weren’t necessarily aware about mental health issues, its not essential but it helps. Even at work, because where I work we look after British people who live or work or travel overseas and there’s a huge issue with British travellers who go overseas and have an episode or get mentally ill and get stuck in some foreign country in a mental hospital and we have to try and get them back to the UK where its safe and things so within the department there are some social work advisers who advise specifically on mental health issues so the people at work have got quite an awareness about mental health and I think that has really helped because you know its easier to, it makes it easier to talk about things. You know somebody says this guy is in a mental hospital, it makes it easier to talk about my own experience and say ‘oh well you know when I was in hospital..’ and people are like, ‘yeah?’ and because we’re talking about it all the time it kind of helps. |  |
|  | Okay that’s helpful, thank you. |  |
