# Supplementary material for: Experiences of friendships of young people with first-episode psychosis: A qualitative study
Source: PLoS One. 2021 Jul 30;16(7):e0255469. doi: 10.1371/journal.pone.0255469 (PMC8323937; doi:10.1371/journal.pone.0255469)
Supplement: S1 File — (ZIP) [file pone.0255469.s001.zip › Transcripts of interviews/Participant 6.doc]

| CH | So what I’m interested is hearing about people in your life who you consider friends or who you might spend time with socially. Is there anyone in your life like that at the moment? |  |
| --- | --- | --- |
| 6 | Yes there’s a few people, yeah. So its not about families? |  |
| CH | I’m interested more in the friends side really than the family but we might come onto family a little bit later as well. So lets think about an example then of somebody who you’ve see socially recently in the last week, someone like that? |  |
| 6 | Yeah I went out with three people from school yesterday. We just went for a drink but I haven’t seen.. That’s the first time for a few months really that I’ve seen them and I think since I’ve had like serious mental health problems I think it has made it a bit more difficult. I think I’ve lost a bit of motivation when I call people up and, yeah, I used to sort of drink quite heavily when I went out and I think there’s a part of it that I’ve tried to cut down on drinking a bit so.. Cos I found that drinking was good, like it helped me to socialise and so maybe cutting down on drinking has made it a bit more difficult. It’s a combination of things I think. |  |
| CH | So do you think drinking helps you socialise because it was something you guys could do together, to go and have a drink with friends or was it more like it gave you a bit of confidence? |  |
| 6 | Yeah. I think it was more that it gave me confidence but it was also like when I see friends it often tends to be to go to the pub with them. Yes, it’s something to do with that as well. |  |
| CH | So thinking about these friends you’ve known for quite a while then from school, how long would that be? |  |
| 6 | Well, I’m 26 now and I’ve known them since I was about 12 or 13. |  |
| CH | Okay then, for quite a long time and you haven’t seen them for a little while before you saw them this week? What was it that prompted you to meet up this week? |  |
| 6 | Well I seem to, you know, to have spoken or texted people saying do you want to meet up and for whatever reason it just hasn’t happened but then eventually we managed to organise a date. |  |
| CH | It was just everyone finding a suitable time? |  |
| 6 | Yeah I think so, cos its like at least one of them definitely knows about the problems I’ve had. Sometimes I feel like, maybe a bit of a burden, sometimes I feel like friends sort of looking after me more than sort of.. Socialising.. Sort of.. |  |
| CH | So do you think your role in your friendships has become different now? |  |
| 6 | Well it’s weird, because some people.. I think there’s only two or three people who know about the problems I’ve had but other people - I’m not sure if they’ve been told but they may not have. I think.. I feel like people are sort of, you know, looking out for me a bit, particularly people that know about the problems that I’ve had. |  |
| CH | What was it about the people you have told that meant they were the ones you told, if you see what I mean? |  |
| 6 | Well some of them.. Because my sister - when I first went into hospital she sort of chose the people that she thought I was closest to and sort of informed them of what was going on and so I think I may have told two people myself and I then she told like a few others but I don’t really talk about it very much to people so I don’t know if they know and how much they know. |  |
| CH | What makes that your decision not to talk about it? |  |
| 6 | I think I’m a bit embarrassed about it and also, I don’t know, it just feels easier to.. You know… Talk about normal things, yeah. And also, I mean I spend most of my time.. Because I’m still living at home with my parents I spend most of my time with my family so like I sort of talk about it with them and then whereas your friends, yeah you.. Sort of.. It just doesn’t really come up. Like nobody really asks about it and I don’t volunteer to talk about it. |  |
| CH | You said before you might go out drinking and now you might still go to the pub but drink less heavily. What other sorts of things do you like to do with friends? Is there anything in particular? |  |
| 6 | I play football quite often and particularly in summer I’ve got one friend who lives near [Local area], and we go to the park in the evenings and there are loads of people playing games there so we just join in. And then I play football every Saturday during football season and we have training once a week so that’s quite good to have a routine, so I see those people quite regularly, but we don’t really socialise that much outside the football. Normally we sort of go for a drink after a game but only for like half an hour or so. Then also I like going to the cinema but I find it quite hard to just sit and have a chat with people without any sort of.. Anything to focus on and I find it quite hard to make small talk and if there’s not an activity then I would normally go to the pub or.. I mean I’ve got one friend who visited me when I was in hospital, I can sort of talk with him about my personal problems but generally I don’t like sort of.. Go out for a coffee with people or stuff like that. |  |
| CH | Has that always been the case or is that recent? |  |
| 6 | I think it has always been the case. |  |
| CH | I think that’s quite common.  I was just thinking about the friend who visited you in hospital and is around a bit more if you do want to talk about that stuff. What is it about that friendship, that’s kind of been exposed to everything, and survived through? |  |
| 6 | Well.. Partly he’s quite different to me. He’s very.. I think he’s quite mature and also he’s quite a strict Muslim so he doesn’t drink and he doesn’t have sex with women also he’s… I think he’s just finished his training as a doctor so he’s like.. He knows about the area and yeah, also, I don’t know.. He’s sort of separate in a way from my other friends. He doesn’t know them that well; he’s met them but he doesn’t really know them so it just seems quite natural to talk.. And also he made quite an effort to come and see me in hospital and it wasn’t like… And basically in hospital I didn’t like having visitors really and he sort of didn’t force his way in but he like.. You know, didn’t give me much choice in the matter which I think in a way is quite good because it meant that he spoke to me while I was in the middle of my problems and yeah.. I think he’s the only person outside my family who visited me. Other people sort of offered to but I think, without saying ‘don’t come’, I…. |  |
| CH | Discouraged him? |  |
| 6 | Yeah. |  |
| CH | Having this guy around.. What was his name? |  |
| 6 | [friend 1]. |  |
| CH | Having [friend 1] around, do you think that made a difference to when you came out of hospital and your recovery since, or do you think it was more your family and other people? |  |
| 6 | I think it did make a bit of difference but it’s really weird actually. I sort of saw him much more during the period while I was in hospital than I do normally but I think it helped. Yeah, it just, you know, having someone.. Like knowing that somebody sort of cared about what was going on, and that he made quite a big effort to come and see me. I guess sometimes you feel like.. Family you know, they’re obliged to come whereas friends are not really. |  |
| CH | Was there anybody during that time who you lost touch with or who’s no longer a friend? |  |
| 6 | I wouldn’t say like, it was because of being in hospital; I think it was more of a gradual thing. Like I seem to have lost contact with people where it has happened over quite a few years I think, rather than since being in hospital. |  |
| CH | When you came out of hospital and were starting to get back to normal everyday life, some people have said that those first few times of seeing friends after being in hospital were difficult but then they kind of fell back into a pattern. Was that the same for you or was it different for you? |  |
| 6 | I think so. Particularly this.. I think they sort of knew what was going on but again I didn’t really talk to them about it so yeah, it was quite hard at the beginning but I think I sort of had to force myself to meet up and.. |  |
| CH | And how did that get easier? What happened to make it easier? |  |
| 6 | I think partly I just started to feel more normal again and also I think since.. I’ve been in hospital twice, the first time was much worse than the second time because I didn’t know. It was completely new to me and it was much more unpleasant the first time and I think since that time I’ve sort of like got used to dealing with the symptoms that I’ve got and how.. They’re sort of integrated into my everyday life now so I’m not really.. I’m not held back as much as I was, I think. |  |
| CH | Thinking about making new friends; Have there been any new friends since your symptoms? |  |
| 6 | Yeah, particularly recently. The second time.. The first time I went into hospital I was really scared of the place and the people. The second time I met someone in the hospital who I then saw for like three or four months quite regularly, but I lost contact with him because his phone suddenly stopped working, but it was quite good to have someone in the hospital who I could be friends with. And then there’s.. I come here once a week and I have met a few people here who seem really nice but I haven’t seen them outside of the activities they do here like film club and they’ve got a football club as well but I’ve seen like a few people through the EIS. And then there’s a housing project, called [housing charity]. They’re now running a football group with [premier league club] so they train every Wednesday and Friday and I’m actually going after this. I’ve met like a few people there like one person in particular who I think I’m seeing either tonight or later in the week so that’s quite good, and I think that its quite good meeting people who have similar experiences. I think that a lot of the services that are offered for people with mental health problems help me to meet people like that. |  |
| CH | What is it that’s good about spending time with people who’ve had similar problems? |  |
| 6 | Well I think partly you’ve got things in common with them and they’re all kind of, trying to get past their problems and I think it’s just nice to know there are other people who are experiencing the same things as you. I haven’t really talked about symptoms and things like that with them. You just sort of know that they’ve had problems as well, so I think that in itself helps. |  |
| CH | I was wondering with those people if you still feel that role of other people looking after you that happens sometimes with your other friends? |  |
| 6 | Yeah. Yes it’s different definitely. |  |
| CH | What sort of person do you think you are with people from here or the other activities at [housing charity]? |  |
| 6 | Well I’d say.. I think people from these places sort of don’t judge you like other people do. I think.. I’d say I feel more comfortable around people at those kind of groups than I do with say my school friends because.. I don’t know. I think I just feel like I’ve got less in common with my old friends now and its good to meet people who I’ve got things in common with. |  |
| CH | And if you’re making friends at the moment, what sort of things would you be looking for in new friends? |  |
| 6 | Well I’m not sure. I think I’d probably want them to be.. Well you know I think I’d probably want to have things in common with them and I’d like them to.. Maybe it would to nice to have somebody that I can sort of look out for as much as they’d look out for me because I think that, my old friends, I think some of them at least are probably thinking like.. They’re probably looking out for me, in a way looking after me and sometimes I don’t particularly like that because it makes you feel.. I don’t know… Fragile, and a bit.. I don’t know it just makes you feel.. Sometimes I’m a bit of a failure. |  |
| CH | So redressing that power balance would be important to you in new friendships? |  |
| 6 | Yeah, that would be nice, yeah. But I mean I don’t really like.. I’m not meeting that many new people so I think that I sort of.. You know, decide what I want when the situation arises. |  |
| CH | Is that something that you would like to be doing more of? Do you think there’s something missing in terms of friendships at the moment? |  |
| 6 | I think I often.. I feel quite lonely quite often. But I’m thinking about applying to university to start next September so I think if all goes well then that’ll be a good opportunity to meet new people. I’ve started part time work and a bit of voluntary work but I haven’t really met people there that I would be seeing outside of work, but it’s still nice to be able to be around new people. |  |
| CH | And if you were able to build up those friendships a bit more or maybe new people when you start uni, what sort of stuff would you want to be doing with them? What’s the gap that needs to be filled? |  |
| 6 | Well I.. Thoughts of like having a girlfriend have pretty much gone out of the window over the last couple of years since being in hospital, so that’s something I definitely think about. I haven’t really got any.. I’m quite close to my sister but apart from that I haven’t got any female friends, so that could be good to meet some girls. |  |
| CH | Has that always been like that or has that been different recently? |  |
| 6 | I think it has been different, but I think again I had female friends at school but pretty much since leaving school really I’ve gradually lost touch with them, so I don’t think it’s to do with being in hospital. I think it would be nice, like university would be good because it’s nice to have people doing the same thing that you’re doing so that you’ve got things in common with them. My school friends.. I feel that I’ve got less in common with them now than I had a few years ago. |  |
| CH | Everybody’s sort of grown, doing different things? |  |
| 6 | Maybe I’ve become a bit cut off from them because of what’s been going on. |  |
| CH | And do you think if you hadn’t had that time in hospital things would have gone a similar way for you as it has for them? Or do you think you are different sorts of people and you would have done different things anyway? That’s kind of a hard question to answer isn’t it? |  |
| 6 | I think I would have been more similar to them if I hadn’t been in hospital and I feel that hospital was like the big hurdle that kind of got in the way of things. I feel like I’ve become closer to my family since being in hospital but I feel I’ve become further away from my friends. |  |
| CH | And if you had to think why that is what would you say? |  |
| 6 | Well partly I feel that I’m spending more time with my family and also they saw first hand what went on so I think, in a way, like in times of crisis that sort of brings people together I think. I mean, I’ve definitely seen my friends less since being in hospital but I think it’s a number of reasons why. I don’t think it’s just because of going into hospital, and if I’m seeing them less it means that I’m seeing my family more. |  |
| CH | We talked a little bit about what you might look for in a friend, how its difficult until you’ve got people in front of you, or are in that that situation, but do you think that process for you now of looking for friends or what you want in a friend is different to before you went into hospital or do you think it would be the same sort of thing? |  |
| 6 | I think its similar things like having things in common. I’m quite into football so its always nice to talk to people about that and I don’t know.. I’ve never really been that kind of fussy about people that I hang round with and I’ve had friends who are quite different to each other really, so yeah I don’t normally.. If I’m with someone, I wouldn’t normally say or think that I don’t like this person because of what they’ve just said or stuff like that. I don’t really think like that. |  |
| CH | So you’re an easy going sort of person? |  |
| 6 | Yeah, I suppose so. |  |
| CH | Okay and lastly thinking about the service here; I know you’ve come to some of the activities, is there anything else that they could do or could have done or could do in the future for other people to support friendships when people are getting better? |  |
| 6 | What, the friendships they’ve already got? |  |
| CH | Yeah. |  |
| 6 | I don’t know really cos its.. I don’t know if people would really want them interfering in their friendships. |  |
| CH | Is there anything they could have done to help, to sort of support you from the background, if you see what I mean? |  |
| 6 | Well, I mean I had some CBT which was quite helpful but we looked at my drinking because I used to drink and I do still drink quite heavily but less often, but that was helpful. I think I don’t need it as much now to socialise so that was good. I dunno. I’ve been really happy with the help that I’ve received and I think really they could offer to get friends in to inform them but I personally I really wouldn’t have wanted that, but maybe some people would want close friends to come.. |  |
| CH | To come in here with you and hear about what the service is? |  |
| 6 | Yeah. |  |
| CH | I think that’s everything actually. Was there anything that you wanted to add or that you think about friendships that we haven’t touched on? |  |
| 6 | Not that I can think of. |  |
| CH | No? Okay, thank you. |  |
