# Supplementary material for: Experiences of friendships of young people with first-episode psychosis: A qualitative study
Source: PLoS One. 2021 Jul 30;16(7):e0255469. doi: 10.1371/journal.pone.0255469 (PMC8323937; doi:10.1371/journal.pone.0255469)
Supplement: S1 File — (ZIP) [file pone.0255469.s001.zip › Transcripts of interviews/Participant 7.doc]

| CH | So what I’m interested in hearing about is people in your life who you see socially and who you consider to be friends. Can you think of anyone like that at the moment? |  |
| --- | --- | --- |
| 7 | Yeah a few people. They’ve all gone off to uni, but there’s a few people in my life. |  |
| CH | What sort of things did you used to do with those people? |  |
| 7 | Just like go out with them to clubs and maybe go to their house every so often, just like sort of talk about anything, like random stuff, girls and stuff. |  |
| CH | What were the bits that you liked best about those friendships? |  |
| 7 | Just that I could go to their house at any time, we’d just be able to talk about anything, and laugh about anything, that sort of thing. |  |
| CH | Did you talk to them about the symptoms that brought you here? |  |
| 7 | No, I haven’t talked to any of my friends about that yet, because some people don’t even know about any of that. One person that knows about it, but I haven’t really talked to anyone about it. |  |
| CH | Why’s that? |  |
| 7 | Just because it’s not something I really want to share with my friends because it was just such a bad time. I don’t really want to share it with my friends. I’d rather let it pass over and then hopefully it’ll get back to the way it was. |  |
| CH | Okay. And in your friendships what sort of role do you think you play? Like someone’s a joker maybe, someone’s the planner.. |  |
| 7 | I’d say I was the joker and people say that I’m funny, I’d play the joker, just you know, the person that cracks jokes all the time. Yeah, yeah, doesn’t take life too seriously. |  |
| CH | And in terms of your friends, what sorts of role do they play? What sort of people are they? |  |
| 7 | Yeah you’ve got one that’s.. I’ve got a friend that’s good with girls, and then I’ve got another one that he’s sort of like goes to church, he’s a Christian guy but he wasn’t like that before but now he is so yeah, he’s a Christian guy, and there’s another one that, how should I put it, I think that’s it, there’s others but I can’t really think.. |  |
| CH | And do you go to friends for different things? Like is there one friend you could go to if you want talk about one thing, somebody else.. |  |
| 7 | Yeah I think so. Yeah I probably go to my friend who’s good with girls if I wanted to talk about girls, if you’ve got their number or something and I’ve got another friend if I wanted to talk about mainly more serious stuff and then I’d go to another friend if I wanted to talk about work, maybe doing an assignment or something like that. |  |
| CH | And what about your friends coming to talk to you - what sort of things would they want to talk to you about? |  |
| 7 | Yeah, well really the same thing. Not anything specific. Its probably the same thing about girls or work or something like that. |  |
| CH | And these are friends that you’ve had for a long time? |  |
| 7 | Yeah. I’ve had like these different sets of friends: university friends and my friends in [City]. The ones in [City] I’ve had for a long time, and then the ones in uni I’d had for seven months by the time I left. |  |
| CH | And the friends from uni, are you staying in touch with them now that you’re not there? |  |
| 7 | Yeah they phoned me on a few occasions, but now its becoming likely that I won’t go back again I don’t really see.. It’s a shame but I don’t see the point in keeping in touch, because if I’m going to another uni then I’ll probably never see them again. It’s a shame but that’s the way I’m thinking right now. |  |
| CH | And is that a direct result of the symptoms that brought you here? |  |
| 7 | Yeah, yeah. Because what happened was that what triggered the psychosis was when I was smoking cannabis in university and then when I came off the cannabis I went home before the term like ended - the second to last term - I went home before term ended just to get off it and then went I went back I had some stuff to sort out, where I was living next year, and my friend came round, and he had some cannabis on him and so I thought to myself ‘right one joint, one toke’ or whatever. I sort of had it but it was stronger than usual so I sort of went off it. I said ‘no, no its too strong’ and then so I went back home, cos there was two weeks left of the holidays so I might as well go home until it’s finished, and then when I went back that’s when it all sort of changed and then like little things started to happen and then, yeah, that’s when I went into the home, the mental home. That’s what sort of triggered it so.. I’ve forgotten the question…. |  |
| CH | I was asking you, is that what led to you not talking to your uni friends? |  |
| 7 | Exactly, yeah. I’ll probably try and visit them this year or next year, but because I’m going to a different uni cos for some reason I feel that I can’t go back, it’ll be like there’s like a tag on me, the tag of the guy who went sort of crazy, so that’s why I feel I can’t really go back there its sort of.. I mean it’s a shame because I liked it. I did like it there, there were people that I was close to, even though it was for a short time. I don’t know about if I can go back there because of that tag. Fresh start at another uni. I’m still not too sure what I’m doing so I’m still like back and forth, yeah, yeah. |  |
| CH | And your friends here, when you came back from uni and the stuff started to happen, you started to not feel quite right, were you seeing your friends from home then when you came back? |  |
| 7 | Yeah, yeah I was. When I came back. No, no actually when I came back the second time I don’t think I was. It was my family I think. |  |
| CH | Are you close to your family? |  |
| 7 | Yeah, yeah, yeah. |  |
| CH | So then when you went into hospital was there anyone who visited you in hospital? |  |
| 7 | No it was family. They were the one’s who knew I was in hospital. Yeah, it was my family. |  |
| CH | And was there anyone in hospital who you made friends with? |  |
| 7 | Yeah, there was this guy called Stuart. He was a good friend. He got me through it basically. He’d been there for a long time so he’d seen people go in and out, so he was a good friend. |  |
| CH | What was it he did that helped? |  |
| 7 | Well just because it was like.. He was only two years older than me so that he was just like.. I talked to him like about anything, like any of my old friends. I just talked to him about anything. The same things, girls, or life in general. We was on the same wavelength more or less, because he had smoked cannabis as well so he was on it for longer than me so we was able to talk about that and different stuff. So yeah, he was a good friend. |  |
| CH | So it helped because he knew what you were…. |  |
| 7 | Yeah, yeah, yeah. |  |
| CH | And when you left there did you stay in touch with Stuart? |  |
| 7 | No I didn’t, cos near the end we sort of drifted apart a bit but I think he lived in my estate. If anything probably he would knock for me or something. |  |
| CH | And when you came out of hospital did you start seeing your friends around here straight away or…? |  |
| 7 | No because I wanted to be by myself for a bit. I turned off my phone. I didn’t talk to any of my friends for like nearly two weeks after I came home and then I turned my phone on eventually and, yeah I think one time my friends rung me to go to a club so I thought I’d just go out. But when I went to the club I didn’t feel myself for some reason, not myself. I wasn’t really joking as much as I would do. I wasn’t enjoying myself. So yeah, I left quite early in the evening, said I’m going home now. That shattered my confidence a bit, I thought ‘why wasn’t I enjoying myself?’, so yeah so at the end of the day after, I started to not really pick up the phone to my friends. I didn’t want to talk to them because I knew it would all be about going back to uni and I didn’t want to talk about it cos it was like I didn’t really… If it was talking about uni it would remind me of all the good times that I’m missing out on. If they wanted me to go out I was worried that I wouldn’t be the same person, I wouldn’t be like the joker any more. I mean like even now it’s the same thing that I’m not really answering my phone to my friends. Maybe my uni friends like talking ‘yeah, I might be coming back’ just so that they know I’m alright, because I didn’t have any touch with anyone for about 3 months, so sometimes I answer my phone to my uni friends to say ‘yeah I’m alright’. But my friends in [City], they want to go out, but I think there’s no point cos I probably won’t be the same person, like joking for some reason so yeah. |  |
| CH | So now you’re seeing friends a lot less than you were before? |  |
| 7 | Yeah, a lot less, yeah. |  |
| CH | It sounds like you were pretty sociable? |  |
| 7 | Yeah, yeah, I was. Yeah I was. |  |
| CH | What do you think needs to happen for you to be able to start doing that again? |  |
| 7 | I think I need to get my confidence back I think, but without having to be in contact with my friends. It’s like, maybe getting a job; my brother’s telling me like door-to-door salesman job. He done it before and he was telling me it increased his confidence. It got him.. It increased his confidence because you’ve got to go door to door and you can’t really be stuck or get an attitude, or.. Can’t take a no to heart, you’ve got to carry on door to door… |  |
| CH | You get quite a thick skin? |  |
| 7 | Yeah, yeah, so I think something like that would just get my confidence back. Just something to get my confidence back to get in contact with my friends. I can’t really tell them why I don’t want to go out or anything because it’s not really the sort of thing I talk to them about. It’s only my family really I can talk about that with. |  |
| CH | So you could talk to your family about that? |  |
| 7 | Yeah. Actually before I wasn’t really saying anything about it, cos I was.. Cos before I wouldn’t really talk to my family about anything. It was sort of keeping everything bottled in and I didn’t want to say anything. But like now because I came back the first time and admit to my family that I had been smoking the cannabis and trying to come off it that’s when I first had a chance to talk to my family probably about anything. About how I feel, the emotion and everything. I’m not the sort of guy to talk about emotion but I had to. |  |
| CH | So do you think your family and you are closer now than you were before? |  |
| 7 | Yeah. That’s a good thing that’s come out of it; my family are closer now. The only bad thing that’s come out of it is that I sort of reached a stage where its like.. Yeah, I’m sort of low now innit? Sort of borderline depressed, just borderline. That’s the only bad thing that’s come out of it, yeah. I mean, I wanted to say I regret it. I probably would say that actually. If I had known this would be the outcome I would never have touched it. |  |
| CH | It takes a little while to get back on your feet. |  |
| 7 | Yeah, yeah it takes a while, a long time so… |  |
| CH | Thinking about if you were going to make new friends now, if you were in a situation where you were going to meet new people, would you still want to look for the same sort of things as your old friends or would you look for something different? |  |
| 7 | Yeah that’s the thing about meeting new people. I would say that, especially my friends at uni, we were all on the same wavelength, were just like funny guys, make each other laugh and all that, so I would say that’s the one thing that makes me want to go back to my old uni so I’m thinking to myself I’m meeting the same people that I met when I was in my old uni. I think meeting new people like, you never know what you’re going to see in that new person, so its like I would go for the same qualities, but then if there was something else, I wouldn’t mind that either. I don’t mind meeting new people, as long as they’re alright, they’re not sort of, they’re not.. I can’t really explain it, as long as they’re not idiots or fools or anything. They’re just like normal, on my wavelength, then yeah, they’re alright. |  |
| CH | If you were going to make a wish list of the sort of things you want in someone, it sounds like having a good sense of humour, being a laugh would be a big thing. Is there anything else you would put down as really important in friends? |  |
| 7 | Yeah just being a joker, joking about, not taking things too seriously, just stuff like that. I don’t like people that take things too seriously, maybe judge other people too quickly, I don’t really like that. I prefer not to take things too seriously just.. You need to have a laugh at any time really, but yeah, that would be the only thing. |  |
| CH | Thinking about the friends that you’ve got, you mentioned how you’re not so much in touch with them as you were before. Is there anybody you’ve stayed in touch with completely throughout, the same as before? |  |
| 7 | No I don’t think so. |  |
| CH | What do you think would have to be different about any of those people for you to still be talking to them lots? |  |
| 7 | What do you mean? |  |
| CH | Well if you’d had a friend who you were still in contact with them, I wonder what they would have needed to be like? |  |
| 7 | I think probably just, be like someone I could talk to about anything, maybe someone like my brother or something or someone who was not really necessarily a joker. That’s a hard question I think, I can’t explain it, what the person would have to be. Probably like someone who… |  |
| CH | You’re doing well. You said someone who’s a bit like your brother and I’m wondering whether your brother is someone who you’ve got a lot of trust for? |  |
| 7 | Yeah, my brother, cos sometimes I have to tell him some stuff, because I’m not used to talking about some stuff like that with him, we usually talk about girls and stuff, or maybe stuff that’s not important, that’s the crucial thing though, yeah, somebody who wouldn’t maybe, who wouldn’t see me in a different way maybe. If I told my friends all this stuff that I’m talking about now, they’d see me in a different light so maybe someone who wouldn’t see me in a different way, they’d maybe say ‘is it’ and then ‘yeah, yeah’. |  |
| CH | So one of the reasons you haven’t talked to your friends is that you don’t want them to see you differently? |  |
| 7 | Yeah. I want them to see me how I was before, just a joker rather than all this stuff, cos it’s long, it’s serious. |  |
| CH | It’s too much? |  |
| 7 | Yeah, it’s too much, yeah. |  |
| CH | Okay, since you started coming here have you been to any of the groups or the social stuff here? |  |
| 7 | Yeah. I went to the football thing once. I haven’t been to any of the stuff up here cos I find that when I come here its like.. Cos there’s people that.. Coming here is like a reminder of what happened before, right? Psychosis and that. Coming here for the social thing reminds me of all the bad stuff that’s happened. I’d rather just come here and talk to [care coordinator] or whatever, and then get out than socialise. It sounds kind of bad, but it’s just because.. The only good thing I could say is that everyone here has problems here that are similar to me, but I’d rather just come here and talk to [care coordinator] and then go, and do something else like go to the gym. It might change, but for now that’s how I’m thinking. |  |
| CH | So in terms of how you spend your spare time at the moment if you want to do some kind of socialising or be out the house, would you go to the gym? |  |
| 7 | Yeah, even then though sometimes going to the gym.. I wouldn’t go before because I might see my friends. The gym I go to is kind of like local so some of my friends, a lot of my friends go there, so sometimes I find myself not going to the gym because I might see someone there and would have to explain why I’m not in uni or why this or stuff like this or stuff like that. I tend to go there and have a quick workout and then go in case I see someone I know and have to talk to them about uni or something like that and then I’d have to make up a reason why I’m taking a break, when really I did want to go back to uni but I couldn’t cos of what happened. So yeah, that’s what sort of happens when I go to the gym. |  |
| CH | Is there anything else that you would do? |  |
| 7 | No not really. The gym’s the main thing, yeah. When I was smoking cannabis I got kind of skinny, and so then when I went back I was kind of skinny so when I went into the home I sort of put the weight back on, so that the gym would be the perfect way to lose the weight again. That’s the only thing really. |  |
| CH | But then you haven’t been able to go there so much as you might have wanted? |  |
| 7 | Yes that’s the thing, that’s sort of annoying. |  |
| CH | Do you think if you had had someone around, a friend throughout you could have talked to, do you think it would have made a difference to how you’re feeling now? |  |
| 7 | Yeah, definitely. If I had a friend I could talk to about this, then it would definitely be different. Then at least there would be at least one person that’s around me that knows everything but because there’s no one to talk to about it, then it just makes it seem, like it makes you regret it more, what happened, because right now it feels like I’m a shadow of myself, like, who I was before. It makes you think why did I do it in the first place, why did I smoke my first spliff? But if I was in uni everything would just be normal, instead I’m.. If there was at least one person it probably would be different. |  |
| CH | Why would talking to somebody like that be different to talking to your family? |  |
| 7 | Well, because like with your family you sort of have to, you have to be open about stuff, you can’t really keep things to yourself, then they keep asking you what’s wrong and then you have to be open because there’s no escape from family, but when its your friends you have the choice to keep it or not. If I could then I probably would share it with one of my friends but cos I can’t share it… does that answer the question? |  |
| CH | Yes. Do you think there is anything anyone could have done to help you stay in touch with your friends better or any help you could have got? |  |
| 7 | No I don’t, cos the way I was feeling I just wanted to be by myself sort of thing, even from my family. Sometimes they’ll say you should - they’ll tell me to phone my friends or something, but if you don’t want to do it then you won’t do it, so I don’t think so. |  |
| CH | If you were thinking about people in your life at the moment, the way your life is at the moment what’s the biggest thing do you think that’s missing for you in terms of friends? What’s the thing you miss the most? |  |
| 7 | Just a laugh, cracking jokes like before, not having to think about all this heavy stuff. Just, I’ve.. My life’s become too serious now, before it was just about jokes and maybe getting some girls. Life wasn’t serious, so that’s the thing I miss, just not to take life too seriously. |  |
| CH | Let me just check if there is anything else I need to ask you.  If you were going to start seeing friends again now would you do the same things as you did before, clubs or would you want to do something different? |  |
| 7 | I’d probably wouldn’t want to go to a club. Maybe just go to someone’s house or something like that. Maybe in the future when I’m in a better mood, feeling a bit better about myself, then maybe I would go back to clubs and all that, so yeah, yeah. |  |
| CH | So your aim is to get back to how things were before? |  |
| 7 | Yeah. |  |
| CH | Okay, let me just check here. I think that’s everything. Is there anything else you wanted to say about friends or how things have worked out? |  |
| 7 | Yeah, no I think that’s everything. |  |
