# Supplementary material for: Experiences of friendships of young people with first-episode psychosis: A qualitative study
Source: PLoS One. 2021 Jul 30;16(7):e0255469. doi: 10.1371/journal.pone.0255469 (PMC8323937; doi:10.1371/journal.pone.0255469)
Supplement: S1 File — (ZIP) [file pone.0255469.s001.zip › Transcripts of interviews/Participant 8.doc]

| CH |  |  |
| --- | --- | --- |
| 8 | My name is [Participant Name] and I am here of my freewill to give information about the research that Catherine is doing and I don’t wish to sign anything because I don’t wish to be legally bound to this I am just here as a natural person sharing the knowledge that I have and its nothing to do for me with the law. I’m here of my freewill and I would like to share the knowledge that I have with Catherine about the research that she is doing so any information I am very happy for her to use for research purposes and to play this anywhere else that she wants and to make this public as well its absolutely fine with me as long as there’s no damage to me, thank you. |  |
| CH | Thank you. I am interested to hear about people in your life who you see socially, people that you consider to be friends. Are there friends in your life at the moment, anybody that you can think of? |  |
| 8 | Yeah, yeah, there are, yeah, definitely. Its really interesting because since the time that I had found myself being really upset, in terms of having so much pressure from, you know, losing my job and basically ending up on my own without parents or anything like that, it kind of brought me to a kind of position where you feel totally unsafe in the world because there isn’t that much help out there and everybody needs to be in contact with someone who’s looking after them. No-one can really do, like on their own. So the moment that I kind of went to my GP, I told him that I was, you know, unhappy, you know, straight away I had anti-depressants and I really found those just kind of knocking me out from my normal state of mind. I could feel that straight away and to be honest with you I was just crying because I was unhappy about my mum and you know I was unhappy about the fact that I lost my job at [Previous Employer]. I had worked for about 4 years since uni and it sent me really crazy, and I was asking questions about what life is and why my life because I’m a very.. I need meaning, and my meaning is not in shopping. It is definitely not in reading any books, you know, that any person has written out there. I know this sounds really crazy but because.. I’m more of a spiritual person. I just, you know, everything that I read I kind of like know it anyway? And it all comes from my spiritual knowledge of being me but that in me was dead before… Like before I basically lost my job it was all dead because I was busy with other things and success and running around and you know, trying to negotiate a better work opportunity, and you know running around doing things so I was very busy. I was shopping as well you know, but I didn’t find any meaning in it. So suddenly I really started asking why, what is the point? Even though I had some money to kind of go shopping I wouldn’t really want to shop any more, so it was a real kind of becoming more of a life inside, more dead to the outside. I don’t see any point in anything I saw before, so when of course you go on the inside its very interesting because its like subconsciously.. You see there’s such a thing as.. There exists something that affects you subconsciously. Yes, the information we collect from life from the point of birth and death but also there is something that drives that information in us. Like something that chooses for us what we will think about you know? We choose, but at the end of the day we don’t really have a choice to chose what to think about. We automatically just have our thoughts in our minds and they just come and we just think about them. We can change our thinking process by doing things, but anyway what I’m trying to explain is that when all these things like my job, like I was saying, and everything, all the upsetting stuff and other stuff that started to happen you know, like I said, made me not really want anything anymore because I thought well what’s the point? I’ve worked for, like, many things so hard and at the end of the day they mean nothing because no-one really cares about your career, and at any time of the day they could stab you in the back and you would be out, you know, and then you’ll have to be searching for another job and my friends are struggling so much. One of my friends has got a job in like Barclays and, you know, she’s saying how hard it is because people are not nice, you know? They’re just not nice in the corporate world, full-stop. That’s all it is and so it really affected me, and I like to work with people who are nice not people who are there for money, you know? It really affects me, so when I got here I was in a state of mind where nothing mattered. I didn’t care about anything, to be honest, that was how it was. As I said, I started asking really deep questions and it made me be.. You know, suddenly something decided for me. This decision deep inside to really find out what life was about, you know? And I was fed up with religion and all those things, you know? I know there is a true meaning, and if anything we think that there is something there, you know, kind of related to God, you know? When you make that assumption it becomes really difficult cos you’re like, what is this God about? All this religion and stuff like that, its really like, there’s a lot of hate towards it, and I have the hate too, and I still have the hate towards religion but anyway sometimes you want to take control, and you’re like, this is what I think. And I lost everything, and I stopped caring, and so I decided to just take a grip of something that I thought was the last thing that would actually give me meaning. So I decided to put right all the crap that was going on in church because I never went to church before, right, but it was my last meaning, so I decided to like, pull it all apart you know? And prove how ridiculous religion is. Just because it was my last meaning. If I couldn’t do that I wouldn’t have any more meaning at all. So that’s what I did. I went to church and I.. Because I always thought that it was ridiculous what people were saying about God. The reason what I wanted to know about God is because that was my last meaning because I lost everything. Okay? So I went, and do you know what? I found my own meaning and it’s so deep that it plays with your subconscious to a point where it makes you so different and it makes you so much better. It’s unbelievable. It is like the strongest drug or pill that you can ever take and on the inside and through the inside and onto the outside your mind will start literally to alter and just you will start thinking in a different way and seeing things than you’ve never seen before, and through that it will give you meaning. It gives meaning of why you have lost your job or why things are going on in families, you know? My mum, for example, or why there are wars, why there are children dying in the news and everything. It’s all to do with politics. Oh my god, it’s so deep. It’s all the spiritual world. It’s 100% related. In fact, it is the absolute of the physical world, and what brought me here, like I said, is this experience of realising that corporate world is a big con. It is not success. It is fake success. It’s the success that eats your soul and this was my realisation. This thing is so strong and so absolute that there is no way you can stop it. Like once you let it in, it completely shows you stuff about the world. Unbelievable things and it changes you and.. But before, of course, because you’re a human being, a human mind, and you’ve collected so much information since the day you were born, you know, it needs to be put right so you become pretty much emotional, you know? You become really emotional and, you know, you start to think about stuff that’s happened, you know, so I’m letting it all out, you know? And so I was letting things out, you know? I mean, came here before I was 18 and I was living on my own. I wasn’t even 18. I was already living on my own and my mum and dad they weren’t there, they left me. So I was always on my own and, you know, it’s difficult because at the end of the day friends say - this is in relation to the question to what you were asking me - friends are people just like you. They have problems just like you. They don’t know any better. All they can do is just spend their time with you and you know, just watch videos and just hang out, but they don’t know any better than what you know because, I mean, you can know your life and they can know their life. No-one else can know your life, no-one. They can just love you and spend time with you but of course when you’re emotional and miserable its very hard for them to do that, you know? Because where you are, in fact, you don’t actually want to let anyone in, why would you? You’re not well so its like I’m ill, you can’t actually come and look after me; you can in the flesh you can come and give me food and bring me things, but in my mind you can’t really just walk in because that’s my house you know – a psychologist tried to walk in and yes they did help but you know there is a house, it’s a spiritual house so unless psychology knows about the spiritual house.. I mean, it tries to walk in through drugs and, you know, chemicals, but really what they are doing is blindly walking into a house that is spiritual and if they don’t understand what spirit it is, they will do things backwards, you know? Through chemicals and stuff like that, and that is quite a challenging road to go down, because you know you just don’t know. You really just don’t know, unless you have the other eyes, you just don’t know what you’re doing, but that’s okay, it’s an extensive field that they’re researching. But on my.. What I would say about friendships is that when I came to this place, I don’t really want to say what this place is because I don’t want to go public, yeah, but when I started my counselling, I basically was, you know, I was really.. Lets say.. Out of my mind, you know? I was really emotional. I didn’t know what was real any more, you know? I didn’t know what was real any more. How can you spend so many years like working so hard and then, you know, working hard for, you know, other people and then suddenly 500 people are being made redundant on the floor that you are working while one of the heads of the company is getting one million bonus. How is that possible? It’s a paradox, you know? It plays with your mind because a lot of people ignore this because they want the money too, and they believe that they will move up, you know, and get the bonus and stuff, that’s why people don’t say anything. But someone who doesn’t want money really, like me, I started to question what is all this about? The true conscious of a human being is to, at the same time as a transaction is going through when they’re in a store, you know, buying a £445 cream you know, I’m in make-up, I’m selling creams at the moment, a bottle of cream is £445. The true conscious is to know that at the same time that your transaction’s going through for that amount of money for your cream that you’re buying, a child is dying. At that very second, a child is dying. You can chose what to know, what to think about, but when you chose to know both things you will then.. It doesn’t mean you’re not going to buy the cream but you will ask the question: what is this about? What is the world about? What is the money about? What are these sorts of things about? They are about lives, living this way. And about a lot of pain and it keeps going round and round, and that’s why I was so upset, you know? Because all that anger through people that lives in people you know. |  |
| CH | These are big issues to deal with aren’t they? Is there anyone that you talk these things through with? |  |
| 8 | Yes. I do, I speak through these issues with my mum, but I think they’re global issues, you know? These are just like global issues and if anything, you know.. Yeah, I kind of.. That’s why it was that so many friends.. That’s a good point. People don’t want to hear this, they just want to go shopping. My friends just want to go shopping. They just want to enjoy this life, you know? I find it very false and I used to buy a lot – I never knew why but now I know - and I find things very pretentious. Unfortunately I’m just not living on the surface of life any more, I am living on truth. I’m standing on truth, walking every day and I am seeing things in truth, as a real human being, the true human being, the one who I was always supposed to be, the one who every true human was supposed to be to walk this earth, to want to know that right now someone is suffering to walk this earth and to be so good in heart that they would never want any one to suffer. No-one you know, no other soul suffer, that is the true human being, but we’re dead in our souls. We’re dead. That’s why we pay no recognition. We have no knowledge, and no we don’t recognise the truth in time and it’s an eternal truth because suffering always existed. That’s why we’re dead eternally a lot of the time. They said the knowledge I received from reading the twisted word of the son of god, because it got totally twisted, you know the people who wrote it probably weren’t even the people who are said to have written it. You know, who knows who wrote what was written, the truth about it is that truth, even if its turned upside down, hidden, re-written, twisted, dah-dah-dah, it still stands. It’s like being one of these dolls you know? If you push it that way, that way, that way, that way and upside down it still turns the other way up. It’s like that’s what truth is. It doesn’t matter what happens to it, you know? It can be written in a lie; it will still be truth because the true meaning is not the word but in the message behind the word, and so I read the word of the son of god. It is twisted, it is, because that’s why people think its foolish, but if you ask for the truth to be revealed to you and know that there is something there, it will be revealed. this is what happens when you read it, its like a pill. You know the film Matrix? You know when they swallow the pill? You have to actually swallow it, so an apple isn’t actually an apple, its a message. An apple is food, there are two types of food; one which is physical. The only way the truth can be told is in the context of this world. That is why apple was used but it’s not actually an apple, it’s a metaphor. The truth can only be told in a metaphor, so that the apple is actually a message - she ate an apple. She had a message; she had a life, so the reverse is the pill that is in the Matrix. It’s very hard to understand this, so what I’m saying is very deep and complicated but why I ended up here and why I became… I lost my friends, some of my friends, and started making new friends. Because the word of the son of god - although it is twisted - when you read it, and if you understand it and if you, you know… Or its like power that you need to plug in.. If you read it and plug it into the source that’s when it’ll light up. You can’t just hold a pill in your hand, you have to swallow it. You can’t just hold the appliance in your hand, you have to plug in to it and then it will light up. You’ve got to plug into the source. So if you have the son of god in your mind you’ve got to plug into god. That’s how it works and it will light up. That’s what happened to me. I did it. I read the word and no matter how twisted it is, I read it, and, you know, and its amazing. What happened after that I cannot, its not… It can’t be told in a human language. It can only be told in the language of heart because you start seeing things. You start seeing unbelievable things, and I guess psychologists can, you know, you know, when you talk to the staff and other people who have never gone through it they don’t hear it at all and so like I said they just end up wanting to go shopping and stuff like that. So in the beginning when you haven’t found people who have experienced what you are experiencing you literally kind of like, need someone to speak to, but at the same time, like with me I became so emotional because I was so upset.. I was on my own. It was scary, you know? Having lost my job, having no money.. I had money and then I ran out. I went shopping. I went crazy. I went shopping and yeah, it was like, it will be all fine, and then I kept going to interviews and I couldn’t get a job because I was seriously, seriously.. I couldn’t believe the way things are. I just really.. I just, to be honest with you, in [City], on my own, I met some people who weren’t really concerned with me they were just concerned with money and that made me alone and that made me unsafe, you know? And so yeah, I had really weird interviews with like, other companies, you know? They would ask me questions that literally knock you out. Like as an example, someone, you know, I was once asked - this is too much, but you know I’ll just tell you how, my experiences, you know, going through all this that I’m saying, people would - at one of the interviews I was asked if I could say no. If I would say no, right? It’s a trick question; it’s a psychological question, you know, but it totally… When you’re upset, when you’re emotional, you really get so upset about people asking you trick questions. Can you say no? Imagine going for an interview, you’ve just lost a £30,000 job, you’ve worked for it for 4 years, 2 years at college before that, you know? You were at high school working really hard, you know? Like your parents are telling you to come on, get on with it, you’ve done everything that you’ve been told to do, you know, you’ve achieved things, you know? You’ve got a job and suddenly, yeah, you’re like right, we don’t want you any more because you’re just an animal. You know, just labouring for us. You and many other people, goodbye now, you know, we don’t really care. We paid you money, and that’s all you were there for, to get money, yeah so… What was I talking about? |  |
| CH | You were saying about how all of that realisation led to you not spending time with the same people; realising that their interests were just about money and about shopping and you were thinking of something deeper.  Can you tell me about the people, the new friends that you’ve found as a result of your discovery? |  |
| 8 | Yes. So the new friends that I’ve found are, in particular, people who are into the truth movement, so this is different. I have found people who are.. Who haven’t had a similar experience to me, but my experience has opened my eyes on what is happening in the world. Like, the government control or how government controls people, how government has 100% or whatever, how government has connections to corporations, how corporations fund, you know, the campaigns for voting and stuff like that, you know? Its all inter-connected and so my new view of how people are controlled, like how I am controlled, how much pressure I’ve been feeling since I was made redundant or you know, being able to survive, how much pressure especially on my own. When you’re on your own you feel it much more. All that I have seen and knew from the inside, from my spiritual self, all those things I’ve seen. I now have caught onto certain events that are happening politically, like the UN and the globalisation of people, ‘depopulisation’ of people. I was ‘depopulised’. I was like… Literally, my Dad came here and I was put on a plane and came to this country, and went to boarding school just because my dad made me. He wanted good for me, that’s the thing, because (Country of Origin] was like, there was stuff going on there and then, but like my dad left and I was already really used to this country so every time I went back to [Country of Origin] I would be alien, like I would not be me. I would be an alien person there. I just, it’s like I came to a different world, you know? It’s a really difficult culture. Everything; mentality so different, so I was already English, you know? I was 10 when I came here so I basically, you know, I absorbed everything as a child, so for me to kind of be one and then absorb a new and then come back to the beginning was impossible, you know? Because there is a beginning and the end, and the beginning in [City of Origin], that was the beginning for me, and like the second step of my life was in England, that was kind of it. I couldn’t like, go back to the beginning, I was already English. So yeah, but what happened was that because of this immigration, because my father was allowed in this country and he brought me here and then he left, for whatever reason, I was alone. I was completely alone. I actually thought ‘whatever is happening?’ People who were working in the Home Office in immigration, they don’t know what’s happening, but the truth of it is immigration is managing population, because I’m now ‘depopulised’ and I’m fully dependent on the government. I don’t have a fathers house or a mothers house or anyone’s house to go to, you know, for help, for when the council decides that, you know, they can’t pay for me any more and I’m afraid that they will because you know the money that I’ve been earning for my under 16 hours a weeks work, you know 65% of that money, any extra money has got to go to them, and so I would be working for £5 for two days, so I actually owe them money. So I am afraid that very soon they’re going to demand this money. I’ve done this Excel table at home of what I owe them, and I’m afraid that they will demand this money and I will say that I don’t have that and the reason for that is that I’ve been buying food and all the money that I have ever had coming in from you and from the part-time work that I’ve been doing is in my bank account. I have.. I don’t use cash, so you can see that I’ve been buying food, you know, I am afraid that they are going to do that and that’s fine but great, so I’m depopulised. I’m pretty much… It’s so intense to try and job search, work part-time when you have to because you can’t be out of work, do all the bureaucratic policies that you must do, and it is bureaucratic - you have to fill out stacks of papers. You have to spend about a day in the housing office to hand these things in. You have to photocopy. I mean, I have panic attacks at home when I go through all the paperwork because I’ve got to have records of everything, because the moment they ask you.. They are so authoritative and so they use the authority of depopulation, like if you don’t do this you won’t have anywhere to live. It is panic. It’s panic that hits you like mad. It goes through the roof, and that is why I spoke already to a couple of people. No surprise that people get in their car or the shotgun and go and shoot people. Because when you’re crazy, you’re crazy, you know? When you receive a letter through the post saying that we’re going to take your house away and that’s what people have been receiving, you know, we’re going to take your house away, people shoot their family because they don’t see a point in anything else. That’s what happened to me. Thank god I saw the point. I saw the new point. Thank god - but these people they go and shoot. |  |
| CH | So can you tell me a little bit about these people who are also part of the movement and who share.. Who you’ve come across as a result of your discovery. |  |
| 8 | So yeah as I was saying basically.. My new view of the world that I have received is very similar to the people that I have connected with through the truth movement. Yeah. These people are all over the world, but I mean in particular I socialise through social networking and they are all over the world. The project out there, the truth movement really started from the moment of 9/11, right from.. Basically I thought that it was an inside job and stuff like that, so yeah, research about the new world government, about control. There are people out there who are doing research and I have connected with them and so these are my new friends, you know? It seems that once you get taken away from success, from wanting success, you know, because you’ve been shown that its actually a con, once you’ve been taken away from that, to be honest you just really want to work for one purpose and that’s to expose, to basically, to you know, participate in projects that are going on globally, you know? These are my new friends. I don’t really want to go shopping any more. I don’t particularly want to be sitting talking about fame or Lady Gaga or you know dah-dah-dah any more. I was never really interested in that. I am more of an intellectual kind of like, you know, I just love things that are like those things I’ve been talking about. I just find the meaning really in all that. |  |
| CH | Is there anyone from before that you have stayed friends with, friendships that have survived through this? |  |
| 8 | Yeah, so there’s those people, and I’m getting involved in some events now obviously that we’ve met, yeah. I just really go around now and I’m sure I am going to meet more and more people and get friends local and stuff, and that’s one part of my friends, and that’s really nice. |  |
| CH | Are friendships developing? |  |
| 8 | Actually yeah, it’s really supportive. It’s really developing, it’s nice. And the other part of the friends.. The friends who were friends before.. Let’s take my mum because she’s kind of always been my friend, but that’s tricky because it’s a family thing going on and I have always been upset with my mum for… Like every time I try to suppress it, we’re friends. In terms of like, normal friends, no I haven’t seen them for almost 2 years now, mainly. When I do see them it’s always really weird, really weird, but at the same time, I don’t know. At the same time, I am keeping in touch with them, but no, I have lost all my friends. I don’t really, you know, yeah.. Just people just… I don’t know, nothing connects you. That’s the thing: what’s friendship about? It’s about more or less a meeting mindset, you know? That’s what friendship is about, just about hanging out and its okay you can hang out and not talk, that’s more or less like a mate, or a husband, who you can just be with in silence, but anything else apart from that is you know.. You’ve got to talk. |  |
| CH | I wonder what part of you wants to stay in touch with them? Because it’s a bit of effort to maintain contact even if you’re not seeing them. I wonder why you do that? |  |
| 8 | It is an effort, it is effort and every time I get quite hurt, you know? Because they dig into my previous attempts at worldly success, and ‘oh well, why don’t you go and look for this job’ or ‘you were doing so well, why don’t you go and do this’ and ‘what you could do is go and do some courses, you know do some investment courses’. I’m like ‘no, I am not interested in you wanting me to get money’. I don’t care about money any more. How about I tell you about things that have nothing to do with money, you know? Lets just talk life as you’ve never known it before. But people don’t just know, they just want to go shopping. So it’s hard, it is difficult. What part of me wants to keep in touch with these, you know, my friends, from, well these particular friends, yeah is really.. I can’t think of anything. I just want to keep in touch with them because I think I can still socialise with them on their level, you know? I can still and although I haven’t been because I’m so busy part time working, doing all the stuff that I’m doing, I am so busy. I kind of want to, so now and then it’s really good. Like two weeks ago I met up with one of my friends from [Previous Employer], I used to work with her, she was my friend from [Previous Employer]. That was really difficult, really, like, challenging. She’s very tired all the time and I found it quite challenging because I’ve always been prompted about my worldly success when I’m not really interested in that any more, you know? I am successful. I am doing what I want to do, you know? I’m not homeless, you know? I’m fine.I’ve got food on my table. I actually deem that as a success but worldly friends like these friends always want to know what I’ve achieved, you know, and they always want to know what I’ve achieved. They always want to know, kind of like, ‘oh come on and tell me..’ There’s always that.. Like there’s always got to be something that’s more than just you. And sometimes it feels like fake stuff. |  |
| CH | Thinking about the new friends that you’re discovering from the development of that knowledge, you’ve got that group of people who you’re in touch with but you’re not getting a lot out of that relationship at the moment and that feels quite difficult when you see them. Is there any gap in your friendships? Is there anything that you would like to be there that’s not in terms of friends? |  |
| 8 | Yeah, I would like people to just consider what I’m saying. Just consider me, that’s all. ‘Cos they want to consider me in the way that they want to consider me. I want them to consider me in the way that I am. |  |
| CH | More openness to what you’re presenting? |  |
| 8 | Yeah, not wanting me to be something, but just kind of like, just not judging. I just really.. If anything, that I would like to be there that isn’t there in my friends it’s consideration. Just to consider what I am trying to tell them when I’m speaking. What I’m talking about, you know? My new me in my kind of research and in my findings that money doesn’t matter. I’ve done the job. I’ve worked for, you know, I’ve done everything that they have wanted to do and they’re still doing, and this stuff just seriously doesn’t matter. Doesn’t matter. The work, I’m just bored with everybody wanting that. Just consideration, that’s what’s not there in my friends, not accepting. Acceptance, consideration you know. |  |
| CH | Thank you, it’s good to hear some really interesting views. |  |
