# Supplementary material for: Experiences of friendships of young people with first-episode psychosis: A qualitative study
Source: PLoS One. 2021 Jul 30;16(7):e0255469. doi: 10.1371/journal.pone.0255469 (PMC8323937; doi:10.1371/journal.pone.0255469)
Supplement: S1 File — (ZIP) [file pone.0255469.s001.zip › Transcripts of interviews/Participant 9.doc]

| CH | So what I’m interested in hearing about is people in your life who you see socially, who you would consider to be friends. Have you got any people like that at the moment? |  |
| --- | --- | --- |
| 9 | Not many. It’s mainly more family members than friends. I’ve had more friends in the past but I don’t know if its because.. I also had depression for a long time before my psychosis. I mean, I think that I always felt like I couldn’t really speak to people, didn’t answer my phone and didn’t really want anyone to come and visit me. I was quite a recluse, kind of thing. I didn’t really want to go out and I only did the things that I absolutely had to do and with the psychosis, like, people are a bit reluctant to call you to invite you to things when you’re depressed anyway, and sometimes when they come and see you and you’re not overly talkative they’re like ‘there’s something wrong with you’. I mean, there probably is but not, something like, you know. I dunno. It’s something like: ‘what’s wrong with you, you’re not talking’ or I think I’m doing something wrong. I found that most people I was telling about my nervous breakdown kind of look at me.. Friends wise, I’ve got one really good friend who understands and is sympathetic and stuff like that but not in an annoying way, like, you know what I mean? In a helpful way. But I’ve had friends in the past take advantage of me because they know I’m a bit sensitive or… Do you know what I mean? |  |
| CH | What sort of thing, can you give me an example? |  |
| 9 | Yeah like..Just taking the piss out of me when I’m actually sitting there. Do you know what I mean? If I question them about it they get like, I’m paranoid, but you’re doing it right in front of me. I’m not. I’m not that paranoid. |  |
| CH | Showing up your weaknesses and trying to get to you? |  |
| 9 | Yeah, yeah. |  |
| CH | Has there been a friend that’s been around through the whole thing, that you have stayed friends with? |  |
| 9 | No. Well, yeah, one of them, but we hadn’t seen each other that regularly. She’s got her life and I’ve got mine. We’re not like… We’re best friends because of how long we’ve known each other and we still call even if we haven’t seen each other for like a whole year, so yeah.. But my other friends saw me as weak like, so that’s why friendships are distant. I feel like someone who might just tag along but not really be that sociable. |  |
| CH | The sort of things you want to do with friends now, is that the same as you would have done before the problems that brought you here? |  |
| 9 | No. My personality has changed a lot through the depression. Some of the things I might or would have liked to have done before I got sick was... Like if I said in the past, I just got shouted down. Then it just made me more withdrawn and quiet, it’s like a vicious circle. |  |
| CH | What sort of things was that, that you would havE thought of doing but wouldn’t have said about? |  |
| 9 | I dunno. Things like I thought was fun. |  |
| CH | And would you do those things more now? |  |
| 9 | I might, but I find that I’ve got to do.. I find that mainly.. I don’t know if its age as well, but I feel like I’ve got to do a lot of stuff of on my own. Not on my own, but no-one’s really interested in art galleries and the things that I am. My cousin has a new girlfriend who does actually, but that’s only recently that I’ve found out. I don’t know. My other friends wouldn’t have done dance with me but you know now I realised it’s because she feels conscious of herself but she would take the piss out of me. Now I realise these people have their own issues - like where they could take the piss out of me because I wasn’t strong enough to defend myself, kind of having a laugh. |  |
| CH | So your contact with those people has become less and less? Do you think that’s because you’ve been getting in touch with them less or do you think they’re getting in touch with you less? |  |
| 9 | Well I think if… I had to distance myself because when someone finds you.. When you become somebody to tag along or whatever, people can tend to use you so, yeah, when I was, like, when I was telling them the way I felt, they was like, there’s something wrong with me because I felt like that. Not with depression, even with the way they were acting towards me, kind of thing, so I distanced myself. |  |
| CH | So you made that decision yourself.  Is there anyone in your life at the moment who’s a newer friend or who has come about since or during your symptoms? |  |
| 9 | My friend [friend 1], I’ve known her like 3 years. I knew her through the depression. She lives at my Nan’s and when I had the breakdown I went to stay there, and I knew her well because she lives on my Nan’s estate. We’re friends but we’ve become much closer since the breakdown. I don’t know whether it’s because of my medication or what. I think it’s a lot to do with the medication; I’m not as fragile as I was and I can now see that I was in a vicious circle with those other people so I don’t need to miss them or anything like that, do you know what I mean? She, my friend [friend 1], she knows about everything what I’ve been through. Yeah, she’s a more supportive one, yeah. |  |
| CH | What sort of thing does she do for you? |  |
| 9 | I don’t know. She’s just there all the time either to talk or if I felt I didn’t want to stay on my own or something. No, she was like there for me but not in a way that was judgmental, or like if I was thinking of my old friends and the stuff I was going through mentally, I know they would just think this girl.. They wouldn’t think ‘oh my god, like maybe you need to see a doctor’, you know what I mean? They’re just different types of people. |  |
| CH | So she’s more of the sort of person that you want to be around now? |  |
| 9 | Yeah definitely. I trust her, which is a big thing for me. |  |
| CH | The sort of things you do with Angie, what sort of stuff would that be? |  |
| 9 | We’ve been raving together, we sit at home and drink tea, smoke cigarettes and chat, but you know just run of the mill stuff like go shopping or she comes up mine. |  |
| CH | Is there anyone else like that at the moment? |  |
| 9 | Yeah, I’ve got a cousin called [friend 2]. And she’s younger than me but I’m so happy that we’ve got closer than we was before because she’s actually about to go through what I went through, but me and her talk a lot about those types of things. Like I can share, like being depressed and how it felt with her And she’s happy that we’re experiencing similar things so she knows it’s not just her. Because you know when you’re really depressed sometimes you can feel you’re the only one in the world that feels like that? And you don’t really know how similar the symptoms are in other people, do you know what I mean? That’s another reason why I kind of kept things to myself a lot because I felt like there was something wrong and I didn’t know how to fix and it stopped me from socialising and being with men a lot, and my other friends always seem to have a boyfriend. I’ll go for a long spells without a boyfriend, and they act weird like as if I’m a lesbian, not that there’s anything wrong with being gay, don’t get me wrong I’m not a homophobic, but they think I must be a lesbian because I don’t have a boyfriend, do you know what I mean? Some people are really.. You just got to ignore that. |  |
| CH | So it sounds as if you were managing; you’ve got two ends of the scale: you’ve got [friend 1] and you talk with her about things, and she’s not being judgemental, and you help [friend 2] too. |  |
| 9 | What she saying, it makes me think of when I was 15. I went through such a period of self-loathing, you know, with this depression. Because I felt like what is wrong with me? And how do I like fix it? Do you know what I mean? How do I just become normal? I just wanted to be normal. I feel like I have missed out on a lot because of depression and… |  |
| CH | What sort of thing do you think you missed out on? |  |
| 9 | I mean I missed out on being a normal teenager. I was so hard on myself because I felt like me feeling shy and not being able to talk.. I thought it was shyness instead of depression, so I used to get angry at myself. I just never felt good enough. There’s something like.. I feel like I could have had a lot of friends. I could have learnt how to have friendships a bit better if I didn’t go through all of that bullshit at a time when I was changing anyway, do you know what I mean? |  |
| CH | It’s a really difficult time when you’re a teenager anyway isn’t it? |  |
| 9 | Trust me. Let alone with the stuff I’ve been through. |  |
| CH | So thinking with all of that in mind now, do you think there’s things that you’re having to do now and practice that other people might have already done when they were younger or do you think that you’re there? |  |
| 9 | No, not at all. I’m practicing meeting new people, practicing putting my barriers down, because even though my cousin[friend 3] and his girlfriend live round the corner I’m the type of person that… You and me are talking cool now, but if you suddenly want to take an interest in me, wanted to come and see me or whatever, I would… Because I’m just like, you know small talk is recently something that I can do but it’s so, it’s so hard for me. It’s hard for me to keep a conversation going. It’s also uncomfortable being in other houses. I feel like I don’t know how to entertain when I do – I grew up in such a sociable household. Even though my dad used to hit my mum and stuff like that, there was also a good side; we did also have fun. I don’t know, I’ve just been through so much changes and so much confusion, so much like.. Feeling at a loss for so long, like how to deal with things has kind of changed my personality. |  |
| CH | And when you first started making those efforts and started practicing the small talk and things like that when the symptoms were starting to get a little bit easier, how did that come about? |  |
| 9 | Well I just, I don’t know, I feel, yeah.. To give you a metaphor, sorry, its like.. I don’t know.. It’s like a tray with loads of glasses full of water and another tray on top of that and piling up and then you’ve got to keep like water in all of these trays but you’ve go to walk and run and stuff like that. To keep the trays balanced it’s like constant, something I’ve constantly got to be concentrating on. |  |
| CH | And that’s how it felt? |  |
| 9 | That’s how it feels now sometimes. Before I just wouldn’t try, I’d be just like I didn’t know… I never felt happy and spent a lot of time suicidal, a lot of the time wishing I weren’t here. Not in a morbid way or a dramatic way, it was just, in a way that that would be the best decision; that would be the best option. Do you know what I mean? |  |
| CH | So being around people, developing relationships takes a lot of concentration and effort from you all of the time? |  |
| 9 | Yeah loads, loads. |  |
| CH | So we’re hearing that [friend 1], she’s quite a different sort of person to your friends before? |  |
| 9 | Definitely, completely different. |  |
| CH | What sort of quality, if you were looking to make some more friends, what sort of things would you be looking for in those people? |  |
| 9 | I’m looking for a down to earth person who’s not judgmental and quite open-minded, and that’s the reason why with [friend 4], even if we don’t see each other we’re cool, because we’re both really open-minded. We don’t judge each other or anything. Like she lives quite a radical life, do you know what I mean? One thing that’s kept us close for this long time is that we can confide in each other without the other one judging. I forgot what you asked? |  |
| CH | About qualities in friends; that’s a good example. |  |
| 9 | Someone who’s got a good sense of humour, someone who’s not judgmental, someone who has a level of maturity as well and intelligent; not like a person has to be a member of Mensa or anything, but what I mean is so we can have a reasonable conversation with them if I do feel comfortable enough to converse. I do have a lot to say and I am… When I write stuff right, I notice that I am quite intelligent, do you know what I mean? What else.. I wouldn’t need them to be caring and loving straight away, because I don’t get that close to people quickly anyway. Just someone that you can talk to who’s not judgmental. I find that most people are, really. |  |
| CH | Do you come across that a lot? |  |
| 9 | Yeah. |  |
| CH | Do you talk about what you’ve been through to people quite early on when you’ve met them or do you wait until you know them quite well? |  |
| 9 | Do you know what, when I was really depressed sometimes I could talk about depression easier if people started talking about it – I was in a hostel with 10 girls, a couple of them girls I knew from primary school, one of them I went to secondary school with and one of them I was in a dance class with her when I was at primary school and we knew each other through that, so them two girls I knew from before. So when we started talking, I did go through a stage when I was just so lost I would just tell people easily what I was going through but I didn’t get.. I just wanted someone to.. I don’t know, I just wanted a way out of it, but it had the opposite effect, kind of thing. Like telling people things too quickly. I was unstable. |  |
| CH | In the old friendships that we talked about, I am thinking of what role you played in those friendships: it was kind of a quieter one in the background, somebody who the others pushed around a bit, it sounds like? What sort of role do you play in your new friendships? |  |
| 9 | I mean the thing is, as well, even though I was being pushed around I am quite a strong person and I can be very defensive and aggressive even, so that’s why it would always be in an underhand way and they would kind of try and deny it kind of thing, but so far as my friends now.. What did you ask me again? |  |
| CH | What sort of role would you play in those friendships? |  |
| 9 | Yeah. I dunno. I am a bit of a contradiction. I give a bit of everything, I think, when I’m ready to give, but then when I ain’t…. I like someone friendship-wise who.. I have learned to keep more things to myself though, that you don’t have to explain everything to everyone because they don’t understand you anyway. Do you know what I mean? I think I play different roles. She can confide in me, my two good friends, they can confide in me. I can be quite comical round [friend 1] as well. I can make people laugh but I dunno. People say I’m nice, I’m warm kind of , I don’t know. What you see and what people see? Because of what I’ve been through I wouldn’t judge them straight away or be rude or anything like that or try and make them feel uncomfortable or anything like that, so I dunno, maybe that’s what makes people sometimes like me, but I don’t know. I like keeping people at bay because of that. A varied role I think I play, like they confide in me; yeah, we can have a laugh . |  |
| CH | I’m thinking about the people that you’ve got in your life at the moment and what they give you, and if there’s anything that’s missing, a gap in what you would like in friendships? |  |
| 9 | Yeah, I have got a gap. I would like a man. But I don’t know. Men are just a different kettle of fish altogether, aren’t they? Do you know what I mean? |  |
| CH | So okay that’s something you’d like, like a partner of some type? |  |
| 9 | Yeah, you know like when you’re young, like a teenager in school and you have a best friend you know, that best friend like that, do you know what I mean? But as you get older it gets replaced with a man, kind of thing. I haven’t moved on to that bit yet, kind of thing. I’ve had relationships but they haven’t lasted that long and I’ve had big gaps in between relationships, do you know what I mean so.. |  |
| CH | So sort of that best friend, that companionship sort of role? And would that be somebody that you would want to talk about your symptoms, the things that have gone on? |  |
| 9 | Yes. See, someone I could trust to not judge me and to not think that I’m nuts or something because being mentally ill is not being mad, do you know what I mean? There’s loads of different types of mental illness. |  |
| CH | Is there anybody that, when you were going through the worst bits, that helped? |  |
| 9 | My mum. My mum and my brother - they never ever like.. I trust them 100%, like they’ll always be there for me no matter how much we argue or whatever, but yeah. |  |
| CH | What did they do that helped? |  |
| 9 | They believed me. When I was saying I was feeling like that, I don’t know, they didn’t treat me like ‘You sure you ain’t mad’ or like they didn’t back off from me because they thought I was the only one that didn’t know. My mum said to me you have to go into hospital, I’m taking you to the hospital, and I just felt like crying and that: ‘no I’m not mad, I don’t need to go to the hospital’ but.. |  |
| CH | Perhaps they showed you what you needed to do. |  |
| 9 | Yeah. |  |
| CH | Is there anyone outside of the family who was around throughout all of that stuff? |  |
| 9 | [friend 1]. Because after I stayed at my mum’s, because it happened at my mums house, and I was kind of scared to go out at first so I started staying at my Nan’s and then me and [friend 1] became closer through.. When I was going through that. And I would go and see her in the evenings, like. |  |
| CH | So if she hadn’t been around do you think things would have been different? |  |
| 9 | Probably. Probably would have felt alone still. I mean yeah, more alone than I did. You know what I mean. |  |
| CH | Is there anything that the service did here to help in terms of people, that social stuff? |  |
| 9 | With the social stuff, I went to one forum, like a group of us and we were all talking about how best to raise awareness and stuff like that of like mental illnesses, and then they were all saying the same things as me. Even though I talked to my cousin Theo when I was having those experiences, I know other people have days like that even when they don’t have depression, but listening to all these people saying they isolated themselves and stuff like that, it helped, kind of thing. |  |
| CH | Did you see any of them afterwards socially or just that one occasion? |  |
| 9 | Yeah, yeah, which is why it was really interesting to see how similar it is for other people.  Some of them had like.. The psychosis seemed to come out of nowhere, do you know what I mean? Yeah, it is frightening. I still feel like it actually happened, that’s the thing. The stuff that I experienced, it was.. I felt like it was actually happening and to think that it wasn’t is just nuts. It was like.. Hard to deal with. But I can’t believe it happened to me. |  |
| CH | Is there anything else you would have wanted the service here to do to help you with being around people, socialising or anything? |  |
| 9 | I have been referred to a behavioural specialist or something - I can’t remember what the names are but I should be hearing from them about that soon actually, because the last person that was here, she referred me. So far as the service here goes, I think it’s cool. [care coordinator] is really lovely, easy to talk to and the medications helping so much so yeah, I think they’re doing everything. |  |
| CH | Has the way in which you might make friends, is that different now? I was just thinking of how you would have met your old friends and how you might meet people now? |  |
| 9 | Well, people that are my old friends, one of them was my friend at primary school, one of them was a friend at secondary school - they are the two that I kind of distance myself from, that were no good. But the other two like, [friend 1] and me still talk so the way that I make friends would have to be different, you see. But I haven’t really made new friends, you know [friend 1] has been 3 years but recently we became much closer in more recent times but.. |  |
| CH | And then [friend 2]’s your cousin isn’t she? |  |
| 9 | Yeah. I’ve got a brother - his girlfriend, she’s cool. I can go and see my brother. I can go and see [friend 1], and other girls from that estate come up there and we chat, but I dunno. It takes a long time for me to make someone - like a friend’s friend, to become my friend. I dunno how I’d make friends now. It would definitely be different because I’m different but I haven’t really made any new friends. |  |
| CH | Do you have any ideas where you might go to meet new friends? |  |
| 9 | Do you know what? I am completely at a loss of how to make new friends. Definitely there’s girls that I met, like, in my first proper job in a call centre. We got on really well, but all I get is like, its hard for me to make friends. And if they are my friends they don’t become my friends until long after we met, do you know what I mean? |  |
| CH | Just to check, have you always been like that? Do you think that it’s taken you a long time to get to know people? |  |
| 9 | No, that’s more recent, yeah. I’m like.. Some people might think that I make friends easily but I dunno. I just seem to find myself in situations where people are taking the piss and talking about me behind my back or.. Do you know what I mean? Maybe I’m too sensitive, maybe that happens to everyone. |  |
| CH | Why do you think that people say you make friends easily? |  |
| 9 | Because speaking about it now.. When I moved into the hostel I met two girls that I used to know and we became close quite quickly, but then that ended badly. My friend [friend 3] - who I was friends with through secondary school - for the last two years anyway, because I changed secondary school and I left all my old friends and we fell out and that’s why I left but that happened. That changed the way I made friends a lot, because I became so defensive. I was not letting no one take the piss again like they took the piss in my old school. I mixed with, like, a harder lot of girls, do you know what I mean? They used to smoke weed and stuff like that. Because I became like that but I wasn’t taking nothing from no other girl. So yeah, even though I became like that I wasn’t quite like them, so I’d kind of be the weaker one. |  |
| CH | Were you smoking weed with them before? |  |
| 9 | I mean, I smoked it very, very occasionally at school but I mean not in the week or nothing, but her mum used to go out in the weekend and used to leave the kids in the house. So a lot of kids from the estate used to come there and I kind of used to go there because there were a lot of boys around, because I was a tomboy in my old school then going through what I went through made me so aware of myself that I started to become girly and started to do my hair and stuff like that, and I didn’t know that I could be attractive because I was always a tomboy. I used to put my hair back in a bun, a round bun and then that was me. I was always in trousers and trainers, but yeah, when I moved I suddenly become more girly and having attention from boys and that was really hard because if we were at my friend’s house at the weekend and her brother and all his friends came in I would be just like mute, because being around men as well was different in, like, the way they would take a type of interest in me. Do you know what I mean? |  |
| CH | Okay. Do you think the way friendships have been for you have impacted on how well you’ve been? |  |
| 9 | Yeah I do actually. |  |
| CH | I mean you mention a vicious cycle, which obviously wasn’t helping. Is there anything else that wasn’t helping? |  |
| 9 | Well yeah, somewhat I’d say.. But also because, I don’t know.. I think they kind of fed each other. Like friendships being.. I dunno, the trouble with the girls and friendships, might have been.. Because I was ill, if you know what I mean, it might be why I got the reaction that I did but I don’t know. Ask me the question again? |  |
| CH | It was about whether the friendships that are around you made things.. impacted on your symptoms, made them worse or better.. |  |
| 9 | Yeah, I think they did feed into each other. |  |
| CH | Is there anything else you wanted to add about your friendships and your experiences? |  |
| 9 | Just like, I don’t know. I can’t really make new friends but yeah I really want a guy that I can trust. |  |
| CH | Thank you. |  |
